# An electronic equilibrium strategy to drive the design of reversible fluorescent probes for sulfur dioxide and formaldehyde

**DOI:** 10.1039/d6sc03350d

**Published:** 2026-07-15

**Authors:** Jiangfeng Li, Jingrui Yang, Yu Wu, Jianuo Liu, Hongze He, Tony D. James, Weiying Lin

**Affiliations:** a Guangxi Key Laboratory of Special Biomedicine, School of Medicine, Institute of Optical Materials and Chemical Biology, Guangxi Key Laboratory of Petrochemical Resource Processing and Process Intensification Technology, Guangxi Key Laboratory of Electrochemical Energy Materials, School of Chemistry and Chemical Engineering, Guangxi University Nanning Guangxi 530004 P. R. China weiyinglin2013@163.com; b Department of Chemistry, University of Bath Bath BA2 7AY UK t.d.james@bath.ac.uk; c School of Chemistry and Chemical Engineering, Henan Normal University Xinxiang 453007 P. R. China

## Abstract

Probes based on Michael addition reactions remain underexploited due to a lack of understanding of the mechanisms governing the reversibility of the reaction, resulting in a lack of theoretical guidance for the targeted design of reversible probes for SO_2_ and formaldehyde (FA). Herein, we propose an electronic equilibrium strategy (EES) to guide the design of reversible fluorescent probes for SO_2_ and FA. To deepen this concept, we further introduce the “reaction electrostatic potential disequilibrium driving force (ESPD) Δ*D*_r_” descriptor to quantify the reaction driving force arising from electrostatic potential disequilibrium. From kinetic and thermodynamic perspectives, modulating the electronic equilibrium state of the probes (adjusting the Δ*D*_r_ value) enables a transition from nearly irreversible to moderately reversible and finally to fully reversible behavior. Moreover, as the electronic distribution of the RE probes progressively shifts toward equilibrium, the fluorescence recovery rates reach 10%, 56%, 91%, and 100%, respectively. Among these, RE-D was used for the reversible imaging of SO_2_ and FA in live cells and mice, as well as for detecting SO_2_ residues in Chinese herbal medicines and FA in cosmetic products. Importantly, encryption ink based on the irreversible nature of RE-A and the reversible characteristics of RE-D enables advanced anti-counterfeiting applications. Overall, this study not only establishes a pioneering design strategy for the rational design of reversible fluorescent probes, but also expands their applications in bioimaging, analytical detection, and smart materials.

## Abstract 2

An electronic equilibrium strategy using the ESPD descriptor Δ*D*_r_ is proposed for designing reversible SO_2_/FA probes. Modulating Δ*D*_r_ shifts reversibility from irreversible to reversible, achieving 10–100% fluorescence recovery.

## Graphical abstract

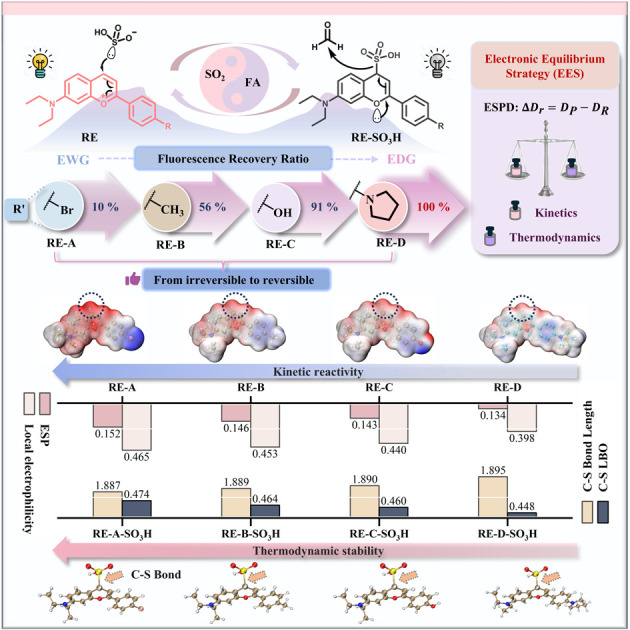

## Introduction

Sulfur dioxide (SO_2_) is a crucial endogenous gaseous signaling molecule in living organisms, involved in regulating key physiological processes such as vasodilation, redox homeostasis, and apoptosis.^1–7^ Additionally, SO_2_ derivatives, such as bisulfite (HSO_3_^−^) and sulfite (SO_3_^2−^), are widely used as preservatives and antioxidants in the food, beverage and pharmaceutical industries.^8,9^ However, research has shown that SO_2_ exposure may contribute to cardiovascular dysfunction and disease by inducing oxidative stress^10,11^ and inflammatory responses^12,13^ and affecting the bioavailability of NO^14,15^ (*e.g.*, promoting the reaction of NO with other free radicals to form harmful substances or reduce its effectiveness). Formaldehyde (FA) is an important endogenous small molecule metabolic intermediate in organisms.^16,17^ At physiological concentrations, FA participates in the methylation of nucleic acids and proteins and plays a key role in the regulation of cell proliferation and differentiation.^18^ Furthermore, due to its excellent preservative properties, FA is also widely used in cosmetics, building materials, and seafood preservation, resulting in these substances being common exogenous exposure sources.^19–21^ However, whether due to endogenous metabolic abnormalities or excessive exogenous exposure, FA exceeding physiological buffering capacity will produce definite cytotoxicity.^22–24^ Excessive FA may consume *S*-nitrosoglutathione through multiple pathways, thereby interfering with the nitric oxide signaling pathway.^16^ This process can have a significant impact on cellular function, especially on the maintenance of cardiovascular homeostasis.^25,26^ It is noteworthy that sulfur dioxide and formaldehyde have a subtle connection in complex signaling pathways. Both SO_2_ and FA can interfere with the nitric oxide signaling pathway, and although their mechanisms of action differ, both ultimately affect the homeostatic regulation of the cardiovascular system. Therefore, developing a tool capable of monitoring SO_2_ and FA is of significant scientific value for revealing their dynamic behavior in physiological and pathological processes.

Fluorescent probes are ideal tools for the real-time monitoring of the dynamic changes of SO_2_ and FA in complex biological systems due to their high selectivity, high sensitivity and excellent spatiotemporal resolution.^27–29^ Among them, probes based on the Michael addition reaction have received widespread attention due to their high selectivity and high sensitivity toward SO_2_.^30–34^ However, while these probes can achieve rapid response to SO_2_, they cannot achieve dynamic monitoring of SO_2_ and FA. To dynamically monitor SO_2_ and FA in biological environments, the development of reversible fluorescent probes has attracted sustained research interest in recent years. Interestingly, probes based on the Michael addition mechanism can exhibit drastically different reversible response behaviors: some enable a reversible response to both SO_2_ and FA,^35,36^ while others respond only to SO_2_.^37,38^ This divergence results in a high level of unpredictability in the design of reversible fluorescent probes for SO_2_ and FA. Therefore, there is an urgent need to develop a novel and effective design strategy for SO_2_ and FA reversible fluorescent probes to promote the targeted design of such SO_2_ and FA reversible fluorescent probes.

With this research, we report an electronic equilibrium strategy (EES) that guides the directional design of reversible fluorescent probes for SO_2_ and FA by modulating the electronic equilibrium state. Based on this strategy, we rationally designed and synthesized four novel RE probes. All probes utilize the α,β-unsaturated benzopyran unit as the SO_2_ recognition site and incorporate bromine atoms, methyl, hydroxyl, and pyrrolidinyl groups, respectively, to modulate the electronic equilibrium state. Quantum chemical calculations elucidated, from both kinetic and thermodynamic perspectives, how different electronic equilibrium states influence the reversibility of the probes. Furthermore, spectroscopic measurements and high-resolution mass spectrometry validated that the reversible response of the RE probes to SO_2_ and FA is progressively enhanced as the electronic state of the probes becomes more equilibrated. Moreover, due to the reversibility of the probe RE-D, it has not only been successfully used for the imaging of endogenous and exogenous SO_2_ and FA in cell and mouse models, but has also been extended to the determination of the content of SO_2_ in Chinese medicinal materials and FA in cosmetics. More importantly, an encrypted ink constructed based on the irreversible nature of RE-A and the reversible characteristics of RE-D enables controllable “writing-erasing-reproduction” of encrypted information and fluorescence verification functions, ultimately building a stable and durable multi-layered anti-counterfeiting and information encryption platform, providing an innovative solution for the development of a new generation of high-security functional materials.

## Results and discussion

### Design strategy and synthesis

**Scheme 1:**
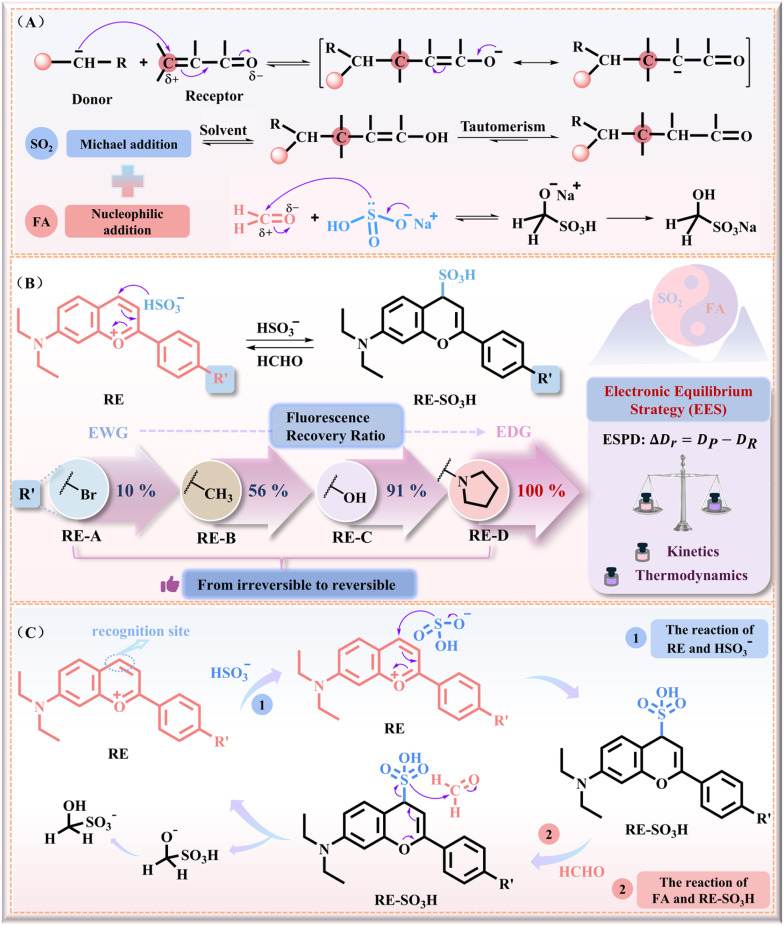
(A) Reported response strategies for SO_2_ and FA fluorescent probes, and (B) the electronic equilibrium strategy for generating a reversible response (this research). (C) Sensing mechanism of RE probes. (EWG: electron withdrawing group; EDG: electron donating group).

Given the nucleophilicity of bisulfite (HSO_3_^−^), this anion can not only undergo Michael addition with α,β-unsaturated bonds to form sulfonic acid adducts, but also engage in nucleophilic addition with FA (scheme 1A). Furthermore, the Michael addition reaction itself exhibits inherent reversibility—a key characteristic that lays the foundation for constructing reversible fluorescent probes targeting sulfur dioxide (SO_2_, primarily existing as HSO_3_^−^/SO_3_^2−^ in aqueous media) and FA. Literature reports indicate that benzopyrylium-based fluorophores bearing α,β-unsaturated moieties can readily undergo Michael addition with nucleophilic HSO_3_^−^ (derived from SO_2_).^39^ Importantly, the equilibrium established between HSO_3_^−^ and its corresponding Michael adduct can be readily disrupted by FA; this disruption imparts reversible behavior to the system. Notably, research has further revealed a divergence in the performance of such Michael addition-based fluorescent probes: some probes exhibit reversible responses toward SO_2_ and FA,^40,41^ while others only exhibit selective responses to SO_2_ and fail to achieve reversible detection of the two analytes.^39,42^ We hypothesize that the activity of the recognition site and the leaving ability of bisulfite are likely the two primary factors affecting the reversibility of these probes. Since the activity of the recognition site and the leaving ability of bisulfite are closely related to reaction kinetics and thermodynamics, the reversibility of the probe is fundamentally governed by whether the probe attains a state of electronic equilibrium. To verify this hypothesis, we performed quantum chemical calculations on the probes described in the two papers by Lin.^43,44^ As shown in Fig. S1, consistent with our speculation, probe NP in a relative electronic equilibrium state exhibited reversible responses to SO_2_ and FA, whereas the probe CP in a state of electronic disequilibrium responded only to SO_2_. To further verify and elucidate this phenomenon, we designed four novel fluorescent probes, RE-A, RE-B, RE-C and RE-D (Scheme 1B and C). All probes utilize the α,β-unsaturated benzopyran unit as the SO_2_ recognition site and incorporate bromine atoms and methyl, hydroxyl, and pyrrolidinyl groups, respectively, to modulate their electronic equilibrium states. We speculate that as the electronic distribution of the RE probes approaches a more balanced state, the reversible response to SO_2_ and FA is enhanced. The synthetic steps and characterization data of the RE probes are provided in the SI (Fig. S2–S19).

### Theoretical analysis of electronic effects

**Fig. 1:**
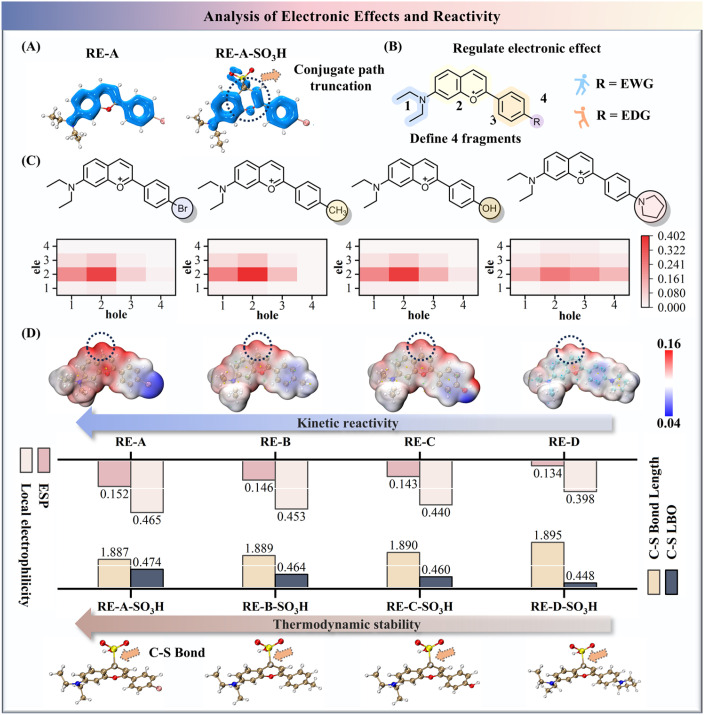
The theoretical calculation of the electronic effects for the RE platform at the B3LYP/def2-SVP level. (A) LOL-π isosurface map. (B) Definition of four fragments. (C) Inter-fragment charge transition density matrix heat map. (D) Parallel analysis of SO_2_ reactivity on the RE platform from the perspectives of dynamics and thermodynamics (molecular electrostatic potentials, Fukui function local electrophilicity, bond length and Laplacian bond order of the C–S bond).

To investigate the influence of the electronic effects of the RE framework on the reactivity of the SO_2_-FA reaction, we performed theoretical calculations using Density Functional Theory (DFT) and Time-Dependent Density Functional Theory (TD-DFT) on the RE platform using the Gaussian16 package.^45^ We first evaluated the molecular origin of the Michael addition of the benzopyrylium RE probe in response to SO_2_, as shown in Fig. 1A and S20.^46^ The π-electron delocalization of the RE platform before and after reaction with NaHSO_3_ clearly shows that the internal π–electron conjugation pathway of both is interrupted after binding with SO_2_. The fluorescence emission blue shift caused by the change in the conjugated structure is the basis for the probe's response to SO_2_. Furthermore, we evaluated the influence of the electronic effects of the RE platform on the responsiveness of the probe through structural fragmentation (Fig. 1B). Analysis of the hole–electron distribution^47^ of the S_0_ → S_1_ state (Fig. S21 and Table S1) indicates that the vertical excitation (S_0_ → S_1_) of the RE platform corresponds to hybrid local and charge transfer (HLCT) and local excitation (LE). For RE-A, the holes and electrons are mainly distributed in fragment 2 and fragment 1, respectively, and the bromine substituent (fragment 4) contributes little to electron transfer (Table S2). However, for RE-D, due to the strong electron-donating ability of the pyrrolidin-1-yl group, the hole distribution in fragments 3 and 4 increases significantly, and the net charge transferred from fragments 3 and 4 to fragment 2 is +0.25 *e* (Table S3). This result can also be intuitively observed in the inter-fragment charge transfer matrix heat map (Fig. 1C), indicating that the electronic effect of the substituent in fragment 4 significantly affects the electron distribution of the benzopyrylium framework. What is special is that RE-D exhibits a state of balanced distribution of holes and electrons.

To further explore the internal mechanism of the RE platform regulating SO_2_-FA reversibility, we carried out a systematic analysis from the perspective of reaction kinetics and thermodynamics (Fig. 1D). First, the molecular electrostatic potential analysis reveals the regulation of different substituents on the overall charge distribution of the molecules.^48,49^ The difference in electron-donating/electron-withdrawing ability of the substituents in fragment 4 directly affects the charge density of the pyran oxygen para-positioned carbon atom. With the transition of substituents from pyrrolidin-1-yl, hydroxyl (–OH), and methyl (–CH_3_) to the bromine atom (–Br), the positive charge of the pyran oxygen para-positioned carbon atom on the RE platform shows an increasing trend. Among them, the charge distribution of the ground state RE-A is extremely uneven, and the ESP of the region near the paracarbon atom of pyran oxygen is as high as 0.152 a.u. (95.22 kcal mol^−1^). From the kinetic point of view, this significant electronic disequilibrium may impart RE-A with a faster HSO_3_^−^ reaction rate, and the stronger electrostatic attraction between the two drives the nucleophilic reaction to proceed efficiently. To obtain direct kinetic parameters, we measured the fluorescence decay of four RE-X probes after adding NaHSO_3_ (500 µM) and fitted each curve using a single exponential function *I*(*t*) = *I*_0_ + *A* × *e*^−*k*_obs_·*t*^(Fig. S22). The resulting pseudo-first-order rate constants (*k*_obs_) are RE-A: 1.3558 s^−1^, RE-B: 0.4566 s^−1^, RE-C: 0.4228 s^−1^, and RE-D: 0.2219 s^−1^ (*R*^2^ > 0.96). The fastest (RE-A) is about six times faster than the slowest (RE-D), confirming a systematic substituent-dependent trend. The Fukui function, as the core parameter of conceptual density functional theory (CDFT), has been widely used to predict the active sites of nucleophilic reactions.^50^ With this research, the condensed local electrophilicity index (*ω*^A^) was used to verify the reactivity trend of the RE platform. The results are consistent with the conclusions from the molecular electrostatic potential analysis, which further confirmed the regulation of substituent electronic effects on the activity of the reaction sites. It is worth noting that the key to the construction of a reversible SO_2_-FA probe is the ability of HSO_3_^−^ to depart from the RE-X-SO_3_H adduct, and the feasibility of this process directly depends on the thermodynamic acceptability of the reaction. In order to evaluate the stability of the C–S bonds in adducts, the C–S bond lengths and Laplacian bond order (LBO)^51^ of different RE-X-SO_3_H adducts were compared and analyzed. Correlation between the C–S bond length of RE-X-SO_3_H and the Fukui function *ω*^A^ value of the RE parent indicates that there is a significant negative correlation between the two (Fig. 1D), and the correlation is matched with the electronic gradient of the substituents from pyrrolidin-1-yl, hydroxyl (–OH), and methyl (–CH_3_) to the bromine atom (–Br): as the electron-withdrawing ability of the substituent increases, the *ω*^A^ value of the RE parent increases, and the nucleophilic reaction activity gradually increases, but the corresponding RE-X-SO_3_H adduct C–S bond energy increases and the stability increases, resulting in a simultaneous increase in the difficulty of HSO_3_^−^ dissociation; in contrast, as the electron-donating ability of the substituent increases, the nucleophilic reactivity of the RE probes decreases, but the thermodynamic feasibility of HSO_3_^−^ leaving increases. This rule clearly shows that only when the RE probe achieves intramolecular electronic balance through the electronic effect of the substituent can it take into account the ideal nucleophilic reactivity (kinetic requirement) and efficient HSO_3_^−^ dissociation ability (thermodynamic requirement), which also verifies that the electronic balancing strategy is the key to the design of reversible SO_2_-FA fluorescent probes.

Based on this, we propose an electronic equilibrium strategy (EES). Its core assumption is that, for electrostatically dominated reaction systems, the degree of disequilibrium in the electrostatic potential (ESP) distribution determines the kinetic reactivity (the higher the imbalance, the greater the reaction driving force), while the degree of balance determines the thermodynamic stability (the higher the balance, the more stable the system). The reaction proceeds spontaneously from a state of high ESP disequilibrium (high driving force) to a state of low ESP disequilibrium (low driving force). Meanwhile, according to the Bell–Evans–Polanyi (BEP) principle,^52,53^ for a series of structurally similar reactions, the activation Gibbs free energy change Δ*G*_a_ is linearly related to the reaction Gibbs free energy change Δ*G*_r_ (eqn (1)):

1 Δ*G*_a_ = *α*Δ*G*_r_ + *C*

where *α* (0 < *α* < 1) is the transition-state position coefficient and *C* is the intrinsic barrier for the homologous series of reactions, representing the inherent kinetic barrier that must be overcome even when the thermodynamic driving force is zero. In short, the more thermodynamically favorable a reaction is, the lower its kinetic barrier. Inspired by this, we endeavoured to construct a descriptor that captures the reaction driving force arising from electrostatic potential disequilibrium (ESPD) and assumed that it exhibits a similar linear relationship with Δ*G*_r_, thereby providing, on the basis of the BEP principle, a rationale for the structural design and regulation of homologous reactions.

Hence, we define two quantitative descriptors for the electronic equilibrium strategy. The first is the system driving force of electrostatic potential disequilibrium (*D*). For the reactant or the forward reaction direction, the driving force is denoted as *D*_R_ or *D*_f_; for the product or the reverse reaction direction, it is denoted as *D*_P_ or *D*_b_. A larger *D* value indicates a greater driving force arising from the electrostatic potential disequilibrium of the system. The second descriptor is the reaction driving force of electrostatic potential disequilibrium, denoted as Δ*D*_r_, which is defined as the difference in the system driving force between the product and the reactant (Δ*D*_r_ = *D*_P_ − *D*_R_). A negative Δ*D*_r_ indicates that the reaction proceeds spontaneously from a state of high ESP disequilibrium (high driving force) to a state of low ESP disequilibrium (low driving force). *D* is a composite descriptor whose components can be obtained *via* molecular surface quantitative analysis and conceptual density functional theory analysis using the multifunctional wavefunction analysis software Multiwfn developed by Lu *et al.*^54,55^ (eqn (2)).2
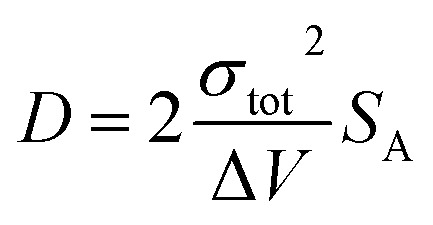
In the equation, *σ*_tot_^2^ is the total variance of the molecular surface electrostatic potential (ESP). Δ*V* is the ESP range (the difference between the maximum and minimum values). *S*_A_ is the relative electrophilicity index or relative nucleophilicity index of the reaction site, *i.e.*, atom A, of a substrate that undergoes a nucleophilic or electrophilic reaction: when the substrate undergoes a nucleophilic reaction, the relative electrophilicity index *s*_A_^+^/*s*_A_^−^ is taken; when the substrate undergoes an electrophilic reaction, the relative nucleophilicity index *s*_A_^+^/*s*_A_^−^ is taken.^56^

To validate the above strategy, we established two homologous reaction models based on the designed reversible probe RE for SO_2_ and formaldehyde and calculated their respective *D* and Δ*D*_r_ values (Tables 1 and 2, S4). The results indicate that for reaction 1, the Δ*D*_r_ values of the four probes are RE-A, −27.57 kcal mol; RE-B, −20.92 kcal mol; RE-C, −16.38 kcal mol; RE-D, −9.95 kcal mol^−1^. The more negative the Δ*D*_r_ value, the larger the observed rate constant *k*_obs_ for the addition reaction, indicating a clear negative correlation. Specifically, RE-A, with the most negative Δ*D*_r_, exhibits the fastest reaction rate, whereas RE-D, with the largest (least negative) Δ*D*_r_, reacts the most slowly (Fig. S23A). For reaction 2, the Δ*D*_r_ values of the four probes are RE-A, −6.74 kcal mol; RE-B, −10.20 kcal mol; RE-C, −11.29 kcal mol; RE-D, −14.44 kcal mol^−1^. The experimentally measured extent of fluorescence recovery follows the order RE-D > RE-C > RE-B > RE-A, which also shows a negative correlation with the Δ*D*_r_ values for reaction 2. That is, the more negative the Δ*D*_r_ value, the higher the formaldehyde-induced fluorescence recovery (Fig. S23B).

**Table 1:** The driving force of electrostatic potential disequilibrium Δ*D*_r_ for reaction 1: RE-X + HSO_3_^−^ → RE-X–SO_3_H

	RE-A	RE-B	RE-C	RE-D
*D*_R_ (kcal mol^−1^)	37.16	31.07	26.80	27.33
*D*_P_ (kcal mol^−1^)	9.59	10.16	10.42	17.38
Δ*D*_r_ (kcal mol^−1^)	−27.57	−20.92	−16.38	−9.95

**Table 2:** The driving force of electrostatic potential disequilibrium Δ*D*_r_ for reaction 2: RE-X-SO_3_H + HCHO → RE-X + HCHO–HSO_3_^−^

	RE-A	RE-B	RE-C	RE-D
*D*_R_ (kcal mol^−1^)	19.97	22.66	23.16	26.10
*D*_P_ (kcal mol^−1^)	13.23	12.46	11.87	11.66
Δ*D*_r_ (kcal mol^−1^)	−6.74	−10.20	−11.29	−14.44

We further calculated the reaction Gibbs free energy changes Δ*G*_r_ for the two homologous reaction models at different computational levels (Tables S5 and S6) and performed linear fitting against Δ*D*_r_ (Fig. S24). It was found that as the computational accuracy increases, the coefficient of determination *R*^2^ of the linear fit becomes higher, while the mean absolute error (MAE), root mean square error (RMSE), and mean squared error (MSE) all decrease. That is, the linear correlation between Δ*D*_r_ and Δ*G*_r_ becomes stronger, and the prediction error becomes smaller, indicating that the accuracy of predicting Δ*G*_r_ from Δ*D*_r_*via* this linear relationship improves. This confirms the core hypothesis: the evolution from reactants to products is accompanied by a change in the overall ESP distribution of the system from unbalanced (high driving force) to balanced (low driving force), wherein the degree of ESP disequilibrium determines the kinetic reactivity, while the degree of ESP balance governs the thermodynamic stability.

In addition, we introduce two probes from different frameworks, RF-1 and RF-2. The consistent results obtained from theoretical calculations and experimental verifications indicate that the correlation between the electrostatic potential disequilibrium driving force Δ*D*_r_ and reaction performance is applicable not only to the original four probes with highly similar structures but also to the expanded chemical structures (Fig. S25 and Tables S7–S9).

In summary, the theoretical calculation results confirm that the electronic equilibrium strategy (EES) of the RE probes can provide core theoretical guidance for the design of reversible SO_2_-FA fluorescent probes by regulating the reaction kinetics and thermodynamic processes.

### Theoretical analysis of optical physical properties

**Fig. 2:**
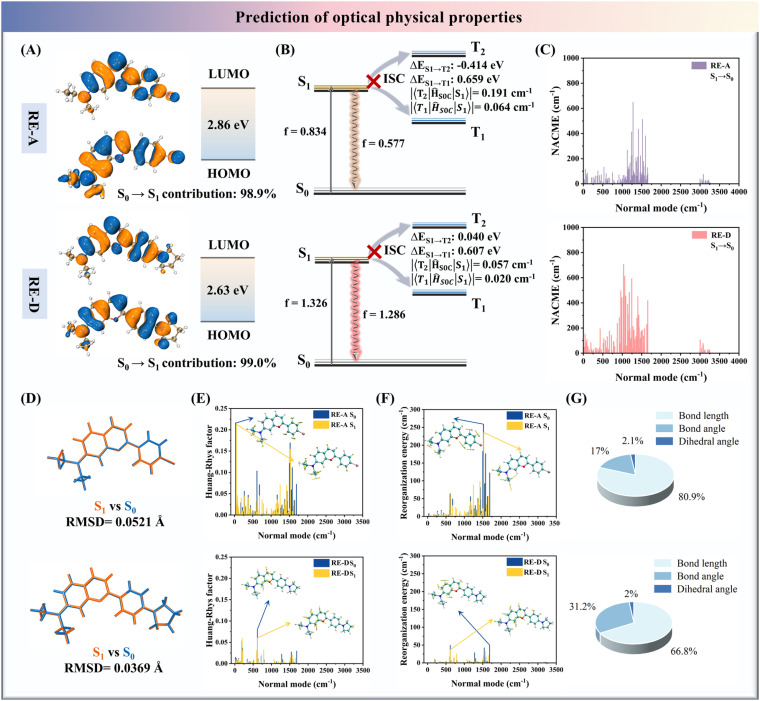
Prediction of the photophysical properties of RE-A and RE-D. (A) The LUMO and HOMO distributions. (B) Photophysical processes of the S_1_ state. (C) NACME of each normal mode for S_1_ → S_0_. (D) Geometric comparison and root mean square deviation (RMSD) between S_0_ and S_1_. (E) Huang–Rhys factor. (F) Reorganization energy (representative normal modes are shown as illustrations). (G) Contributions of bond length, bond angle and dihedral angle to the total reorganization energy.

Based on the potential value of both RE-A and RE-D in the SO_2_-FA fluorescent response, we evaluated their radiative transition processes (*k*_r_) and non-radiative transition processes (*k*_nr_) using the Molecular Materials Property Prediction Package (MOMAP 2024A).^57–62^ As shown in Fig. 2A and Table S10, compared with probe RE-A, RE-D has a lower HOMO–LUMO energy gap, which corresponds to a red shift in the spectrum. Since most molecules follow Kasha's rule in solution, we studied the photophysical processes of the S_1_ state of the RE platform (Fig. 2B). The oscillator strengths (*f*) of excitation and emission of RE-D are significantly greater than those of RE-A. We further calculated that the *k*_r_ values of RE-A and RE-D are 8.76 × 10^7^ s^−1^ and 2.10 × 10^8^ s^−1^, respectively, indicating that the fluorescence imaging potential of RE-D is stronger. Meanwhile, to systematically evaluate the intersystem crossing (ISC) ability of the probes, we performed quantitative calculations on the energy gap between S_*n*_–T_*m*_ and the spin–orbit coupling (SOC) matrix elements, and the results are shown in Fig. 2B and S26. The data indicate that the probability of ISC occurrence in both RE-A and RE-D is at a low level. This feature confirms that the RE series of probes have a weak ability to generate reactive oxygen species (ROS) through the ISC process, thereby confirming that they have low phototoxicity, which provides key theoretical support for the safe application of such probes in biological systems. Moreover, through comparative analysis of data on the non-adiabatic coupling (NAC) between the S_1_ and S_0_ states of the RE platform (Fig. 2C), root mean square deviation (RMSD) (Fig. 2D), Huang–Rhys (HR) factor (Fig. 2E and S27), and total reorganization energy (Fig. 2F and G), it is clear that probes RE-A and RE-D have similar non-radiative transitions, with *k*_nr_ values of 6.46 × 10^8^ s^−1^ and 1.50 × 10^6^ s^−1^, respectively. However, probe RE-A exhibits a greater non-radiative transition capacity than RE-D. Based on the analysis of radiative and non-radiative transition processes, RE-D exhibits superior potential for fluorescence imaging, which is consistent with the results of relative fluorescence quantum yields measured in 1,2-dichloroethane (RE-A: 0.029; RE-D: 0.24) (Table S11).

### Spectral response toward SO_2_ and FA

To validate the capability of the RE probes to recognize SO_2_ and FA, the optical properties of both probes in response to NaHSO_3_ and FA were investigated in PBS buffer (10 mM, pH 7.4, containing 5% acetonitrile (CH_3_CN), v/v). As shown in Fig. S28A and 3A, probe RE-A (10 µM) exhibited an absorption maximum at 525 nm and an emission maximum at 642 nm (red fluorescence). This result confirms that RE-A possesses a significant Stokes shift (∼117 nm), which can effectively avoid crosstalk between the excitation and emission spectra and thereby eliminate false-positive signals. Furthermore, the fluorescence was progressively quenched by up to 31-fold upon titration with NaHSO_3_ (Fig. S28B). The fluorescence intensity exhibited a good linear relationship with the NaHSO_3_ concentration within a range of 0−50 µM, and the detection limit was calculated to be about 1.7 × 10^−6^ M (Fig. S28C). This indicates that probe RE-A exhibits good fluorescence response to NaHSO_3_. Additionally, RE-B, RE-C, and RE-D displayed maximum absorption peaks at 526 nm, 573 nm, and 601 nm, respectively (Fig. S28D–F). This indicates that a red shift in wavelength occurs when the terminal group of the RE probes is modified from an electron-withdrawing group (EWG) to an electron-donating group (EDG).

**Fig. 3:**
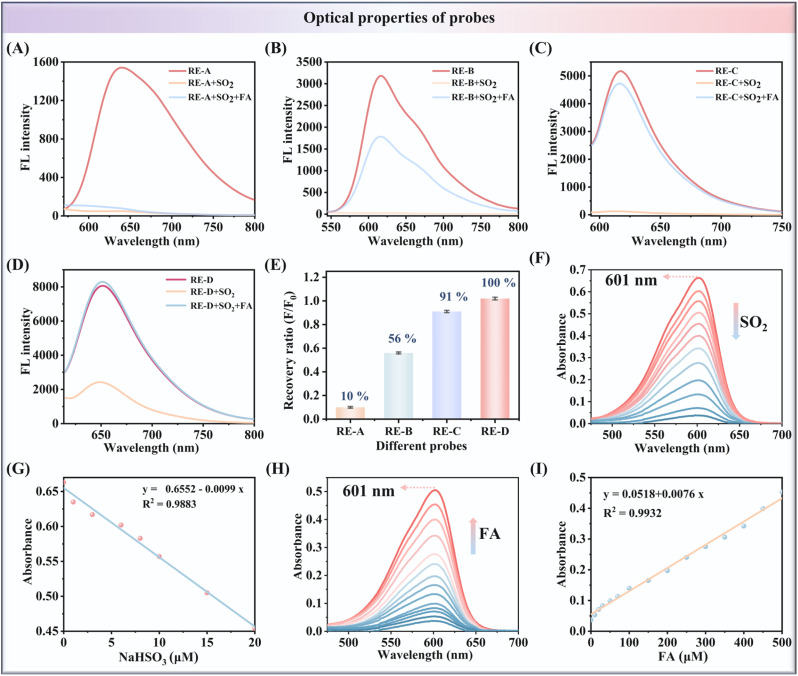
(A) Fluorescence spectra of RE-A (10 µM) before and after response with NaHSO_3_ and FA; (B) fluorescence spectra of RE-B (10 µM) before and after response with NaHSO_3_ and FA; (C) fluorescence spectra of RE-C (10 µM) before and after response with NaHSO_3_ and FA; (D) fluorescence spectra of RE-D (10 µM) before and after response with NaHSO_3_ and FA; (E) Fluorescence recovery ratio (*F*/*F*_0_) of different RE probes; (F) absorption spectra of the probe RE-D (10 µM) upon reaction with different concentrations of NaHSO_3_ (0–500 µM); (G) linear relationship between the absorbance of the probe RE-D (10 µM) and NaHSO_3_ concentration (0–20 µM); (H) absorption spectra of the probe RE-D (10 µM) after reaction with NaHSO_3_ (500 µM), followed by the addition of different concentrations of FA (0–500 µM); (I) linear relationship between the absorbance of RE-D-SO_3_H and FA concentration (0–500 µM).

To evaluate the reversibility of these probes with respect to FA, RE probes were first incubated with NaHSO_3_ followed by the addition of FA at varying concentrations. The fluorescence recovery rates of the RE probes reached 10%, 56%, 91%, and 100%, respectively (Fig. 3A–E). The observed progressive enhancement in the probe's reversibility is attributed to the electronic distribution shifting toward a more equilibrated state, in agreement with the quantum chemical calculations. To further validate the observed optical changes and elucidate the mechanism, high-resolution mass spectrometry was performed on RE-A and RE-D, representing the minimal and maximal degrees of reversibility, respectively. As shown in Fig. S29, treatment of RE-A with NaHSO_3_ yielded a major mass spectrometric signal at *m*/*z* 436.0222, which is identical to that of RE-A-SO_3_H ([M–H]^−^: 436.0223). Analogously, upon treatment of RE-D with NaHSO_3_, a distinct peak at *m*/*z* 427.1701 was observed, which is consistent with RE-D-SO_3_H ([M–H]^−^: 427.1697) (Fig. S30). Interestingly, no new peak was generated ([M–H]^−^) after treating RE-A with NaHSO_3_ and FA (Fig. S31). These results demonstrate that although RE-A exhibits a sensitive response to SO_2_, it is not a reversible fluorescent probe for both SO_2_ and FA. Conversely, upon treatment of RE-D with NaHSO_3_ and FA, a new peak at *m*/*z* 347.2124 ([RE-D + HSO_3_^−^ + FA]^+^) (Fig. S32) emerges, corresponding to the calculated molecular weight of RE-D ([RE-D]^+^: 347.2118). The data indicate that RE-D serves as an excellent reversible fluorescent probe for SO_2_ and FA. In summary, these results validate the rationale of our proposed electronic-equilibrium strategy. By modulating the electronic equilibrium state of the probes, the degree of reversibility of the fluorescent probes for SO_2_ and FA can be tuned, achieving a transition from nearly irreversible to moderately reversible and finally to fully reversible behavior. This research establishes a pioneering design strategy for the rational development of such reversible fluorescent probes.

Given that probe RE-D exhibits a reversible response to SO_2_ and FA, we subsequently performed a visible absorption titration experiment on RE-D. The visible absorption of RE-D gradually decreased with increasing concentrations of NaHSO_3_ (Fig. 3F). Within a concentration range of 0–20 µM, the absorption of RE-D exhibited a linear correlation with SO_2_ concentrations and the detection limit was 1.26 × 10^−6^ M (Fig. 3G). Moreover, we examined the absorption spectral properties of RE-D (10 µM) pre-treated with SO_2_ upon exposure to varying concentrations of FA. Notably, upon addition of FA, the characteristic absorption of RE-D-SO_3_H at 601 nm gradually increased (Fig. 3H) and exhibited a good linear relationship over a wide range (0–500 µM) with a limit of detection (LOD) of 1.6 × 10^−5^ M (Fig. 3I). Based on the above observations, these results demonstrate that RE-D is suitable for the reversible detection of SO_2_ and FA *in vitro*.

Additionally, within 60 minutes, RE-D (with and without the addition of SO_2_ and FA) exhibited high chemical stability (Fig. S33)—a property that constitutes one of the basic requirements for the application of the probe for *in vivo* and *in vitro* experiments. Notably, a practical reaction-based fluorescent probe must exhibit a selective response to its intended target in complex biological systems. To address this criterion, the fluorescence response of RE-A and RE-D to a series of potential interferents was systematically evaluated. The results revealed that no significant alteration in fluorescence intensity was observed in the presence of competing biologically relevant species. (Fig. S34 and S35). Importantly, we examined the reversibility of RE-D-SO_3_H (generated by treating 10 µM RE-D with 500 µM NaHSO_3_) in the presence of other potential competing analytes. Fig. S36 indicates that formaldehyde (FA) was the optimal candidate for probe RE-Ds’ fluorescence recovery; in contrast, the other evaluated analytes exhibited minimal fluorescence responses. These data indicate that the adduct RE-D-SO_3_H possesses superior selectivity toward FA over other competing species. Moreover, it was observed that across the physiological pH range, the efficacy of probe RE-A for SO_2_ recognition (Fig. S37) and the reversible detection performance of probe RE-D for SO_2_ and FA (Fig. S38) were largely unaffected. Collectively, these optical characteristics demonstrate that RE-A and RE-D have the potential for further application in biological imaging and the evaluation of practical samples.

### The response of RE-D*in vitro*

**Fig. 4:**
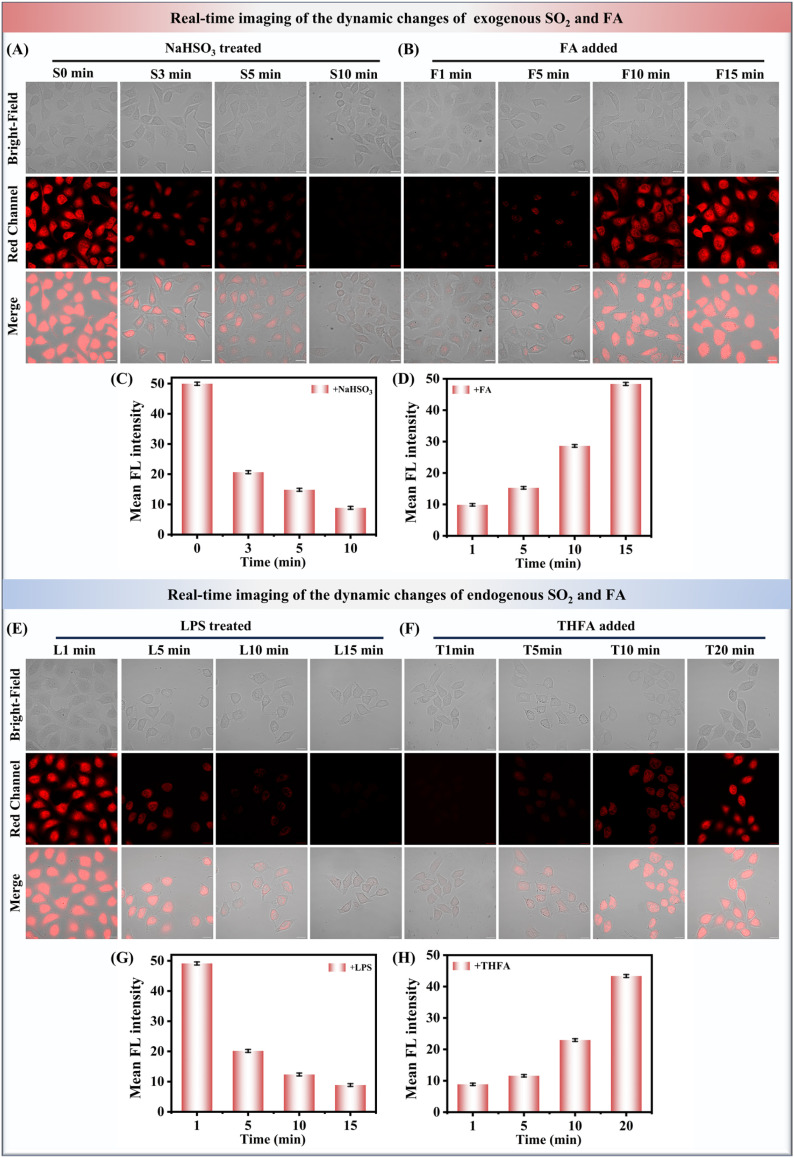
(A) HepG2 cells were subjected to incubation with 10 µM RE-D for 10 min and then to treatment with 100 µM NaHSO_3_, with analyses conducted at 0, 3, 5, and 10 min. (B) HepG2 cells were subjected to incubation with 10 µM RE-D and 100 µM NaHSO_3_ for 10 min, followed by treatment with 200 µM FA and analyzed at 1, 5, 10, and 15 min. (C) Mean FL intensity of imaging in cells with exogenous SO_2_. (D) Mean FL intensity of imaging in cells with exogenous FA. (E) HepG2 cells were pretreated with 1 µg mL^−1^ LPS for 6 h, then incubated with 10 µM RE-D for 10 min, and analyzed at 1, 5, 10 and 15 min. (F) HepG2 cells were pretreated with 1 µg mL^−1^ LPS for 6 h followed by the addition of 10 µM RE-D for 30 min, then incubated with 200 µM THFA for 30 min, and analyzed at 1, 5, 10 and 20 min. (G) Mean FL intensity of imaging in cells with endogenous SO_2_. (H) Mean FL intensity of imaging in cells with endogenous FA. *λ*_ex_ = 561 nm; *λ*_em_ = 570–670 nm. Error bars represent standard deviation (±S.D), *n* = 6. Scale bar: 20 µm.

Given that an ideal bioimaging probe must be minimally invasive, the cytocompatibility of RE-D was assessed. MTT assays indicated low cytotoxicity (Fig. S39), affirming its safety for biological studies. Having established the *in vitro* characteristics and reversible response of RE-D toward SO_2_ and FA, we aimed to explore their potential interplay within the complex environment of living cells using this probe. Therefore, the real-time interplay between exogenous SO_2_ and FA in living cells was investigated by leveraging the molecular imaging capabilities of probe RE-D. As shown in Fig. 4A and C, HepG2 cells incubated with RE-D for 10 minutes exhibited intense red fluorescence. However, subsequent treatment with 100 µM NaHSO_3_ led to a time-dependent decrease in this fluorescence, consistent with SO_2_ disrupting the benzopyrylium π-conjugation to form a cellular RE-D-SO_3_H adduct. In addition, following the addition of FA (200 µM) to the pre-stained cells, the red fluorescence intensity was nearly fully restored (Fig. 4B and D). This recovery implies that FA effectively captures the liberated bisulfite group, thereby driving the dissociation of the RE-D-SO_3_H adduct and regenerating the free probe RE-D within the cellular environment. Taken together, these results verify the utility of probe RE-D in real-time observation of exogenous SO_2_ and FA dynamics in live cellular environments.

Based on the success of RE-D in monitoring exogenous SO_2_ and FA interactions in live cells, we next aimed to track their endogenous dynamics *in situ*. For this purpose, two chemical stimulants were employed. The first was lipopolysaccharide (LPS),^63–65^ a bacterial endotoxin reported to activate multiple signaling pathways in macrophages and induce the production of cellular pro-inflammatory factors. This process is a key contributor to intracellular SO_2_ production. The second was tetrahydrofolate (THFA), a key component that participates in the one-carbon metabolic cycle and thereby facilitates the production of endogenous FA.^66^ Control experiments were designed to validate the detection of endogenous SO_2_. As demonstrated in Fig. 4E and G, the HepG2 cells exhibited intense red fluorescence after incubation with 10 µM RE-D for 10 min. However, pretreatment of cells with LPS (1 µg mL^−1^, 6 h) led to the quenching of this fluorescence in the absence of any added exogenous SO_2_. To establish a negative control, FA was added to inhibit bisulfite. Consistent with expectations, LPS-pretreated cells incubated with RE-D exhibited no notable fluorescence reduction after FA treatment (Fig. S40), demonstrating that the initial quenching was indeed caused by LPS-induced endogenous SO_2_. Therefore, probe RE-D is an effective tool for the monitoring of endogenous SO_2_ in live-cell systems. To evaluate the capability of RE-D for monitoring endogenous FA, HepG2 cells were subjected to pretreatment with 1 µg mL^−1^ LPS for 6 hours followed by the addition of 10 µM RE-D for 30 minutes, and then incubated with 200 µM THFA for 30 minutes. As shown in Fig. 4F and H, the appearance of intense red fluorescence signaled endogenous FA, attesting to the probe's ability to image it *in situ*. Therefore, the results from these experiments verify the efficacy of RE-D for visualizing endogenous FA in live cells. Taken together, these results confirm that probe RE-D enables the real-time visualization of endogenous SO_2_ and FA dynamics in live cells.

### Reversible response to endogenous/exogenous SO_2_ and FA *in vivo*

**Fig. 5:**
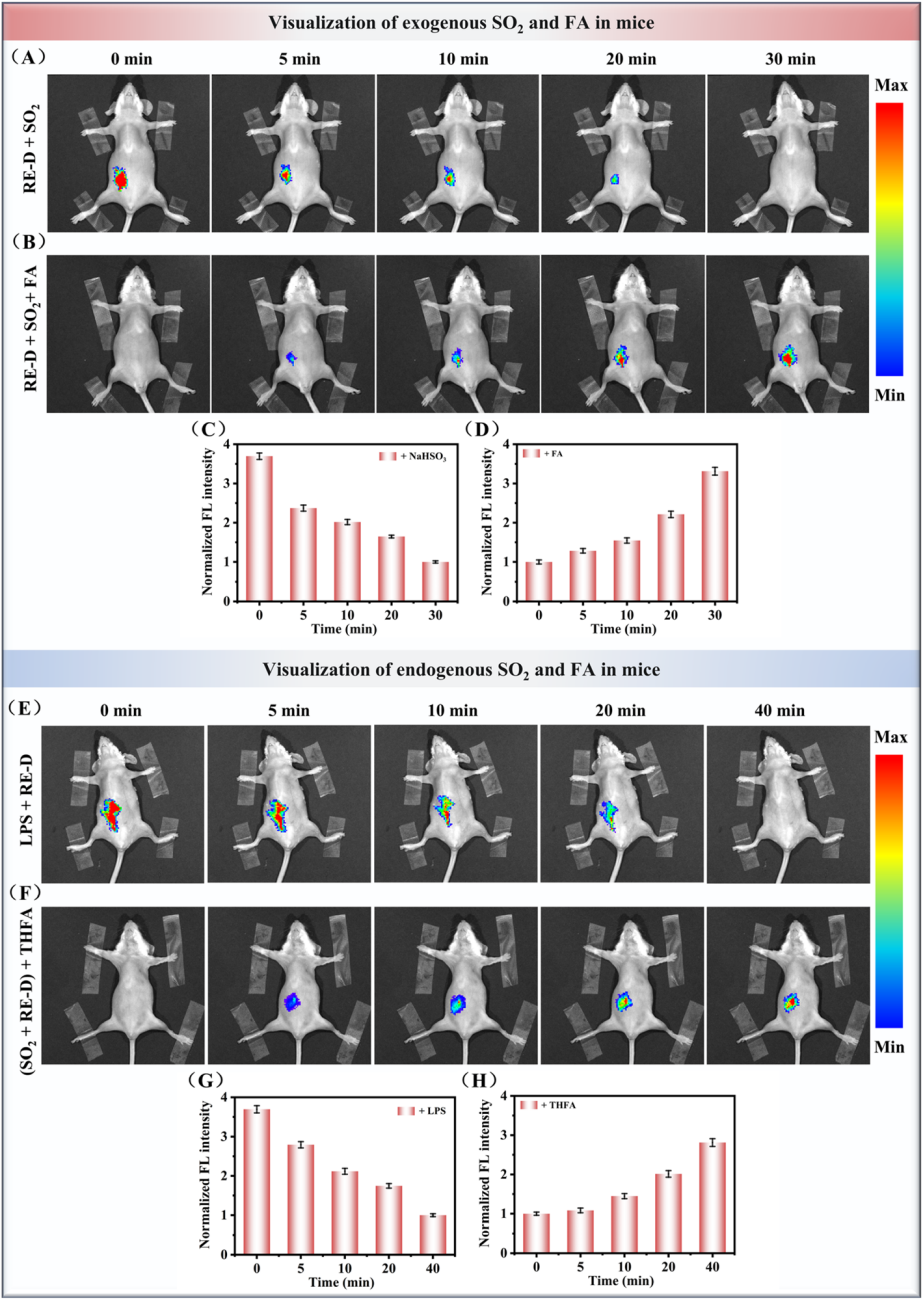
(A) Mice were incubated with 100 µL of RE-D (50 µM), then treated with 100 µL of NaHSO_3_ (500 µM) and analyzed at 0, 5, 10, 20 and 30 min. (B) The mice were incubated with 100 µL of RE-D (50 µM) and 100 µL of NaHSO_3_ (500 µM), then treated with 100 µL of FA (1 mM) and analyzed at 0, 5, 10, 20 and 30 min. (C) Normalized FL intensity of imaging in mice with exogenous SO_2_. (D) Normalized FL intensity of imaging in mice with exogenous FA. (E) The mice were pretreated with 100 µL of LPS (2 mg mL^−1^) for 24 h, then incubated with 100 µL of RE-D (50 µM) and analyzed at 0, 5, 10, 20 and 40 min. (F) The mice were incubated with 100 µL of RE-D (50 µM) and 100 µL of NaHSO_3_ (500 µM), then treated with 100 µL of THFA (1 mM) for 30 min and analyzed at 0, 5, 10, 20 and 40 min. (G) Normalized FL intensity of imaging in mice with endogenous SO_2_. (H) Normalized FL intensity of imaging in mice with endogenous FA. *λ*_ex_ = 600 nm; *λ*_em_ = 670 nm. Error bars represent standard deviation (±S.D), *n* = 6.

Long-wavelength emission offers advantages including minimal photodamage, deep tissue penetration, and reduced autofluorescence interference.^67^ Leveraging the long emission wavelength (653 nm) of RE-D, we envisioned applying this unique chemical tool to reversibly track the real-time interplay between exogenous SO_2_ and FA in live mice. Accordingly, the mice injected with RE-D exhibited strong red fluorescence (Fig. 5A). However, a time-dependent decline in red fluorescence was observed in mice following injection with NaHSO_3_ and RE-D (Fig. 5A and C). In contrast, strong red fluorescence was detected in mice following the sequential administration of NaHSO_3_, RE-D, and finally FA (Fig. 5B and D). Taken together, these data demonstrate that probe RE-D is capable of reversibly detecting SO_2_ and FA in mice.

To explore whether the probe RE-D was able to reversibly track endogenous SO_2_ and FA in mice, the real-time dynamics of their interaction was investigated. Mice treated with RE-D for 30 min exhibited strong red fluorescence. However, when the mice were pretreated with 100 µL of LPS for 24 h and then incubated with 100 µL of RE-D, the red fluorescence gradually faded even in the absence of exogenous SO_2_ addition (Fig. 5E and G). These results establish that the fluorescence signals in RE-D and LPS-treated mice are attributable to endogenous SO_2_ production, validating the utility of RE-D for visualizing endogenous SO_2_ in live mice. Subsequently, to evaluate the capability of RE-D for imaging endogenous FA, the mice were first injected with 100 µL of NaHSO_3_ and 100 µL of RE-D, followed by treatment with 100 µL of THFA for 30 minutes. Imaging was performed at 0, 5, 10, 20, and 40 minutes. As illustrated in Fig. 5F and H, the gradual emergence of strong red fluorescence suggested the presence of endogenous FA. The imaging data confirmed the ability of probe RE-D to visualize endogenous FA in live mice. Collectively, these data establish RE-D as a suitable tool for the real-time visualization of endogenous SO_2_ and FA crosstalk in live mice.

### Detection using real samples

**Fig. 6:**
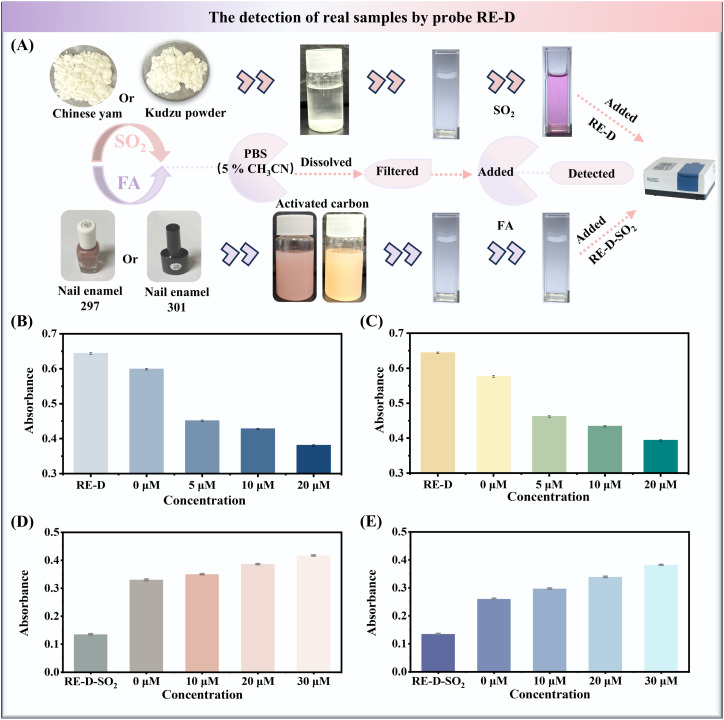
(A) Preparation of traditional Chinese medicinal material and nail enamel samples. Absorbance of filtrates from different Chinese medicinal materials with varying SO_2_ concentrations after addition of probe RE-D (10 µM): (B) Chinese yam filtrate; (C) kudzu powder filtrate. Absorbance of filtrates from different nail enamels with varying FA concentrations after addition of RE-D-SO_3_H (10 µM RE-D + 500 µM NaHSO_3_): (D) nail enamel 297; (E) nail enamel 301.

It is well known that sulfur-fumigation can generate sulfur dioxide for sterilization and insecticidal treatment.^68^ However, studies have shown that the residual sulfur dioxide in traditional Chinese medicines treated using this method may cause respiratory symptoms such as coughing, chest tightness and throat irritation after long-term exposure.^69^ Therefore, the sulfur dioxide content in traditional Chinese medicines should be strictly controlled. According to GB 2760 (2011), the residual limit of sulfite in medicinal materials and their decoction tablets should not exceed 400 mg kg^−1^.^70^ Given the excellent sensing performance of RE-D for sulfite detection, we attempted to use RE-D to determine sulfite concentrations in actual traditional Chinese medicine samples (Fig. 6A). To verify the response ability of probe RE-D to SO_2_ in real medicinal material matrices, we selected Chinese yam and kudzu powder as representative samples and measured the absorbance changes of their filtrates after adding different concentrations of SO_2_ (0, 5, 10, and 20 µM). As shown in Fig. 6B and C, with increasing SO_2_ concentration, the absorbance at 601 nm of both medicinal material filtrates gradually decreased, and the response patterns between the two were essentially consistent. The absorbance change trends of *Poria cocos* and *Angelicae dahuricae* filtrates were consistent with those of yam and kudzu powder; the relevant spectra are provided in the SI (Fig. S41). These results indicate that probe RE-D can produce a concentration-dependent signal response to SO_2_ in different medicinal material matrices, providing a basis for its application in real sample detection.

The sulfite contents of the four medicinal materials (Chinese yam, kudzu powder, *Poria cocos*, and *Angelica dahurica*) were determined by the standard addition method. The calibration curves obtained by the standard addition method in each sample matrix, as well as the detailed data of spiked recoveries at different concentrations, are presented in the SI (Fig. S42 and Table S12). The spiked recoveries of sulfite in the four medicinal materials ranged from 93.15% to 104.92%, with relative standard deviations of 1.21% to 5.11%, confirming that probe RE-D can accurately quantify bisulfite (HSO_3_^−^) in actual traditional Chinese medicine samples. The sulfite contents in the four medicinal materials measured by this method were highly consistent with those determined by the method described in Chapter 2331 of the *Chinese Pharmacopoeia* (2020 Edition, Volume IV) (Table S14), further demonstrating that probe RE-D can accurately determine sulfite content in real samples.

FA is a colorless gas with a strong pungent odor and has been widely used in wood processing and textile production and as an antiseptic.^19^ However, skin contact with FA may induce adverse lesions such as allergic dermatitis, pigmentation, and skin necrosis.^21^ Notably, the International Agency for Research on Cancer (IARC) classifies FA as a Group 1 carcinogen,^20^ while the World Health Organization (WHO) identifies it as the third major indoor pollutant.^71^ According to the Safety and Technical Standards for Cosmetics (2015 Edition), cosmetics with FA content exceeding 0.05% must be labeled accordingly; spray products shall not contain detectable FA; and the maximum allowable FA content (calculated as free formaldehyde) in other products is 0.2%.

Given that the adduct RE-D-SO_3_H exhibits good linear detection performance for formaldehyde over a wide concentration range, we attempted to use RE-D-SO_3_H to determine formaldehyde concentrations in four nail polish samples. Taking two representative nail polishes as examples, after adding RE-D-SO_3_H solution to nail polish sample solutions spiked with different concentrations of formaldehyde (0, 10, 20, and 30 µM), an increase in absorbance was observed (Fig. 6D and E); the corresponding spectra of the other two nail polishes are shown in the SI (Fig. S43). The free formaldehyde content in the four nail polish samples was quantitatively analyzed by the standard addition method. The calibration curves obtained by the standard addition method in each sample matrix and the spiked recovery data at different concentrations are provided in the SI (Fig. S44 and Table S13). The spiked recoveries of formaldehyde in the four nail polishes ranged from 87.11% to 102.96%, with relative standard deviations of 1.16% to 5.28%, indicating that this method also has good accuracy and precision in cosmetic matrices. The limits of quantification for formaldehyde in the four nail polish matrices ranged from 15.11 to 32.58 mg kg^−1^, all meeting the detection requirements of the *Safety and Technical Standards for Cosmetics* (Table S14). The determined formaldehyde contents in the four nail polish samples were 34.60, 69.55, 77.58, and 164.05 mg kg^−1^, respectively, all below the regulatory limit of 0.2%. The relative errors between the formaldehyde content results determined by this method and those obtained by the GB/T 23993-2009 standard method were within an acceptable range (Table S15), further verifying the applicability and reliability of this method in cosmetic matrices. In summary, the above results confirm that RE-D-SO_3_H can effectively detect free formaldehyde in actual cosmetic samples, and all tested commercially available samples comply with the current regulatory limit requirements.

### Anti-counterfeiting applications

**Fig. 7:**
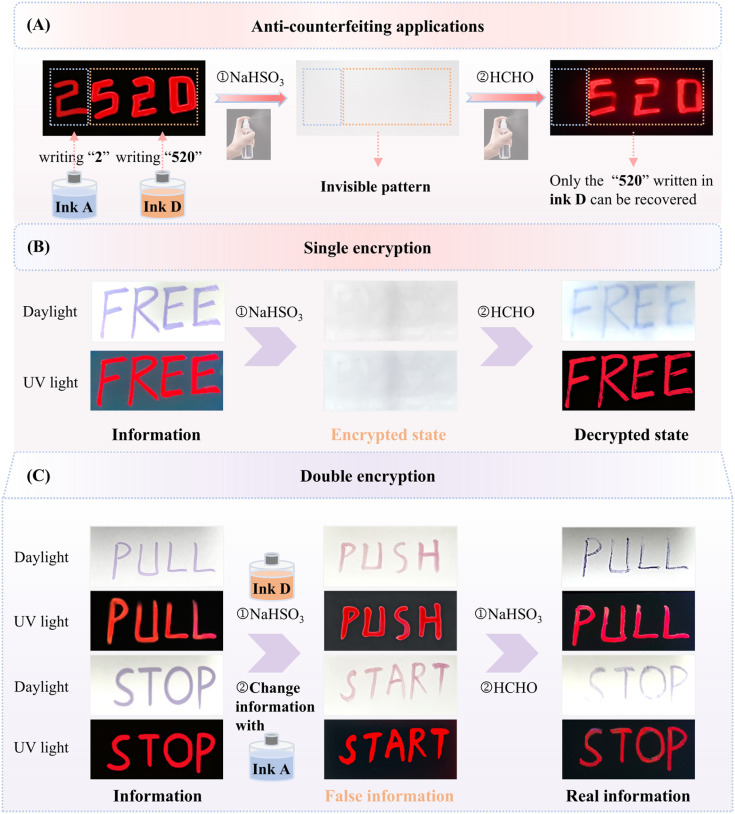
(A) Anti-counterfeiting process using probes RE-A and RE-D. (B) Single-Plex anti-counterfeiting image of probe RE-D. (C) Dual anti-counterfeiting image for the comprehensive application of probes RE-A and RE-D. After the information to be encrypted is treated with NaHSO_3_ solution, the wrong information is added. The correct information can only be displayed after being treated with chemical reagents NaHSO_3_ solution and FA solution in sequence. The displayed encrypted information also exhibits fluorescence.

Since probe RE-A displays excellent irreversible color change and probe RE-D exhibits outstanding reversible color change in response to SO_2_ and FA, their respective advantages can be integrated to formulate Ink A and Ink D for high-end anti-counterfeiting applications (Fig. 7A). As illustrated in Fig. 7B, after writing the letters “FREE” on a thin-layer chromatography (TLC) plate with Ink D, it emitted bright red fluorescence under 365 nm ultraviolet (UV) light. Upon spraying with a NaHSO_3_ solution, however, the letters became invisible under both daylight and 365 nm UV light. Notably, subsequent treatment by spraying with a FA solution restores the letters, along with their red fluorescence. This confirms that Ink D is viable as an encrypted ink for anti-counterfeiting applications.

Furthermore, as shown in Fig. 7C, the information to be encrypted (“STOP” and “PULL”) was written on a TLC plate using Ink D. Spraying with a NaHSO_3_ solution caused the target encrypted information to disappear. Subsequently, Ink A was used to rewrite false information (“START” and “PUSH”) for obfuscation. An additional spray of NaHSO_3_ solution eliminated all information—both the encrypted and false content. Only after further spraying with an FA solution did the correct encrypted information become visible. These results demonstrate that integrating RE-A and RE-D enables multi-layer encryption and high-grade anti-counterfeiting functions, which hold significant potential for chemical and optical anti-counterfeiting applications.

## Conclusions

In conclusion, to address the core issue of the lack of clear theoretical guidance in the design of reversible fluorescent probes for SO_2_ and FA, we proposed an electronic equilibrium strategy (EES). To deepen this concept, we further introduced the “reaction electrostatic potential disequilibrium driving force (ESPD) Δ*D*_r_” descriptor to quantify the reaction driving force arising from electrostatic potential disequilibrium. From both kinetic and thermodynamic perspectives, this strategy enables tuning the probe reversibility through modulation of the electronic equilibrium state (adjusting the Δ*D*_r_ value). Guided by this strategy, four novel RE probes were designed and synthesized. Quantum chemical calculations and spectroscopic results revealed that as the electronic distribution of the RE probes progressively shifts toward equilibrium, the fluorescence recovery rates of RE-A, RE-B, RE-C, and RE-D achieve 10%, 56%, 91%, and 100%, respectively. In other words, it facilitates a transition from nearly irreversible to moderately reversible and finally to fully reversible behavior. This research introduces a pioneering strategy to guide the development of reversible fluorescent probes targeting SO_2_ and FA. Moreover, due to the reversible nature of the probe RE-D, it was not only used for imaging analysis of endogenous and exogenous SO_2_ and FA in cell and mouse models, but was also used for the determination of the actual content of SO_2_ in Chinese yam and kudzu root and FA in nail polish. In addition, encryption ink fabricated based on the irreversible nature of RE-A and the reversible characteristics of RE-D enabled the controllable “writing-erasing-reproduction” of encrypted information and fluorescence verification, resulting in a stable and durable multi-layered anti-counterfeiting and information encryption platform, providing an innovative solution for the development of a new generation of high-security functional materials. In summary, through a systematic exploration of “design strategy – mechanism verification – multi-scenario applications”, this research promotes the development of the directional design of reversible fluorescent probes for SO_2_ and FA. It provides new tools and technical support for fields such as biomedical imaging, food and drug detection and information security, holding significant scientific value and promising application prospects.

## Ethical statement

All animal procedures were performed in accordance with the Guidelines for Care and Use of Laboratory Animals of Guangxi University and approved by the Animal Care & Welfare Committee of Guangxi University (protocol number: Gxu-2024-120, China).

## Author contributions

Jiangfeng Li: methodology, investigation, software, formal analysis, writing – review and editing. Jingrui Yang: investigation. Yu Wu: writing – original draft, investigation. Jianuo Liu: investigation. Hongze He: investigation. Tony D. James: writing – review and editing. Weiying Lin: resources, writing – review and editing, supervision, funding acquisition.

## Conflicts of interest

There are no conflicts to declare.

## Supplementary Material

SC-OLF-D6SC03350D-s001

## Data Availability

The data supporting this article have been included as part of the supplementary information (SI). Supplementary information: detailed experimental procedures, synthetic protocols, characterisation data (NMR and HRMS), photophysical spectra, real-sample testing, cell imaging, and quantum chemical calculations. See DOI: https://doi.org/10.1039/d6sc03350d.
