# Supplementary material for: An electronic equilibrium strategy to drive the design of reversible fluorescent probes for sulfur dioxide and formaldehyde
Source: Chem Sci. 2026 Jul 15. Online ahead of print. doi: 10.1039/d6sc03350d (PMC13370742; doi:10.1039/d6sc03350d)
Supplement: SC-OLF-D6SC03350D-s001 [file SC-OLF-D6SC03350D-s001.pdf]

Supplementary Information (SI) for Chemical Science.
This journal is © The Royal Society of Chemistry 2026

Supplementary Information

An electronic equilibrium strategy to drive the design of reversible fluorescent probes for sulfur dioxide and formaldehyde

Jiangfeng Li[a], Jingrui Yang[a], Yu Wu[a], Jianuo Liu[a], Hongze He[a], Tony D. James*[bc] and Weiying Lin*[a]

[a] *Guangxi Key Laboratory of Special Biomedicine, School of Medicine, Institute of Optical Materials and Chemical Biology, Guangxi Key Laboratory of Petrochemical Resource Processing and Process Intensification Technology, Guangxi Key Laboratory of Electrochemical Energy Materials, School of Chemistry and Chemical Engineering, Guangxi University, Nanning, Guangxi 530004, P. R. China.*

[b] *Department of Chemistry, University of Bath, Bath BA2 7AY, UK*

[c] *School of Chemistry and Chemical Engineering, Henan Normal University, Xinxiang 453007, P. R. China*

E-mail: t.d.james@bath.ac.uk (T.D. James); weiyinglin2013@163.com (W. Lin).

**Corresponding Author.*

E-mail: t.d.james@bath.ac.uk (T.D. James); weiyinglin2013@163.com (W. Lin)

Table of Contents

Experimental Section S5

1. Experimental instruments and reagents S5
2. Quantum chemical calculation details. S5
3. Synthesis and characterization of **RE-A**. S6
4. Synthesis and characterization of **RE-B**. S7
5. Synthesis and characterization of **RE-C**. S7
6. Synthesis and characterization of **RE-D**. S8
7. Synthesis and characterization of **RF-1** and **RF-2** S9
8. Measurement of optical spectroscopic properties S10
9. Detection limit of **RE-A** for SO_2. .... S10
10. Culture and preparation of the HepG2 cells S10
11. Cytotoxicity assay. S11

12. FL imaging of probe **RE-D** in live mice. S11
13. Preparation and determination of real samples. S12
14. Matrix effect assessment. S12
15. Limit of quantification. S13
16. Preparation of encrypted ink S13

Supplementary table **S14**

Table S1. .... S14
Table S2. .... S14
Table S3. .... S15
Table S4. .... S15
Table S5. .... S16
Table S6. .... S16
Table S7. .... S17
Table S8. .... S17
Table S9. .... S17
Table S10. .... S18
Table S11. .... S18
Table S12. .... S18
Table S13. .... S19
Table S14. .... S19
Table S15. .... S20

Supplementary figures **S20**

Figure S1. Analysis of electrostatic potential and reversibility of probes **CP** and **NP**. S20
Figure S2. The ^{1}H NMR of **RE-A** in DMSO-d6. .... S21
Figure S3. The ^{1}H NMR of **RE-B** in DMSO-d6. .... S21
Figure S4. The ^{1}H NMR of **RE-C** in DMSO-d6. .... S22
Figure S5. The ^{1}H NMR of **RE-D** in DMSO-d6. .... S22
Figure S6. The ^{13}C NMR of **RE-A** in DMSO-d6. .... S23
Figure S7. The ^{13}C NMR of **RE-B** in DMSO-d6. .... S23
Figure S8. The ^{13}C NMR of **RE-C** in DMSO-d6. .... S24
Figure S9. The ^{13}C NMR of **RE-D** in DMSO-d6. .... S24
Figure S10. HRMS spectrum of **RE-A**. S25
Figure S11. HRMS spectrum of **RE-B**. S25
Figure S12. HRMS spectrum of **RE-C**. S26

Figure S13. HRMS spectrum of **RE-D**. S26
Figure S14. .... S27
Figure S15. .... S27
Figure S16. .... S28
Figure S17. .... S28
Figure S18. .... S28
Figure S19. .... S29
Figure S20. .... S30
Figure S21. .... S30
Figure S22. .... S31
Figure S23. .... S31
Figure S24. .... S32
Figure S25. .... S33
Figure S26. .... S33
Figure S27. .... S34
Figure S28. .... S35
Figure S29. .... S35
Figure S30. .... S36
Figure S31. .... S36
Figure S32. .... S37
Figure S33. .... S37
Figure S34. .... S38
Figure S35. .... S38
Figure S36. .... S39
Figure S37. .... S39
Figure S38. .... S40
Figure S39. .... S40
Figure S40. .... S41
Figure S41. .... S41
Figure S42. .... S42
Figure S43. .... S42
Figure S44. .... S43

References S44

Experimental Section

1. Experimental instruments and reagents

Common reagents or materials were obtained from commercial suppliers without further purification except as otherwise noted. UV-vis absorption spectra were obtained on a Shimadzu UV-2700 spectrophotometer (Japan), and fluorescence spectra were measured on a HITACHI F4700 fluorescence spectrophotometer (Japan). The fluorescence imaging of cells were performed with Olympus FV3000 confocal microscope (Japan). The NIR fluorescence imaging of the mice was performed with a Small Animal In Vivo Imaging System (IVIS Lumina Series III). ^{1}H and ^{13}C NMR spectra were measured on a Bruker Avance III HD 600 digital NMR spectrometer (Germany), using tetramethylsilane (TMS) as internal reference. High resolution mass spectrometric (HRMS) analyses were measured on Waters UPLC G2-XS Qtof (USA). TLC analysis was carried out on silica gel plates and column chromatography was conducted over silica gel (mesh 200-300), both of which were purchased from the Qingdao Ocean Chemicals. All aqueous solutions were prepared with ultrapure water obtained from a Milli-Q water purification system (18.2 MΩ cm).

2. Quantum chemical calculation details.

The theoretical calculations were performed via the Gaussian 16 suite of programs. The DFT calculations at the B3LYP[1]/def2-SVP[2,3] level of theory, and further processed employing the Multiwfn 3.8 (dev)[4–10] and VMD 1.9.3[11] programs simultaneously. Radiative ($S_1 \rightarrow S_0$) and nonradiative ($S_1 \rightarrow S_0$) decay rate (k_r, k_{nr}), Huang-Rhys factor and reorganization were calculated using MOMAP 2024A[12–17] software. The quantitative verification scheme for the electronic equilibrium strategy employs density functional theory (DFT). Geometric optimization is performed in Gaussian 16 software, single-point energy calculations are performed in ORCA 6.0.1 software, and thermodynamic quantity calculations are performed in Shermo 2.6 software. First, at the B3LYP functional, D3BJ dispersion correction, and Def2SVP basis set levels, the IEFPCM solvent model is used to consider solvent effects, and the system's geometry is optimized and vibrational frequency analysis is performed, confirming the absence of imaginary frequencies and obtaining a stable minimum configuration. Subsequently, Multiwfn 3.8(dev) software is used to quantitatively analyze the molecular surface electrostatic potential (ESP)

and perform conceptual density functional theory (CDFT) analysis on the optimized structure to obtain the various indices required for the calculation of two key new descriptors and other relevant indices. Given the limitations of the B3LYP functional in thermodynamic quantity calculations, and the fact that the IEFPCM model does not explicitly define the contribution of the nonpolar portion to thermodynamic quantities, high-precision single-point energy calculations were performed using the wB97M-V functional and the Def2-TZVP basis set level to obtain the system's free energy in solution. Simultaneously, single-point energy calculations suitable for dissolution free energy calculations were performed using the M062X functional, D3ZERO dispersion correction, and the 6-31G(d) basis set level. Finally, the system's free energy in solution was obtained by adding the high-precision gas-phase free energy to the dissolution free energy, and then the Gibbs free energy change (ΔG) of the reaction was calculated[18–23].

3. Synthesis and characterization of RE-A.

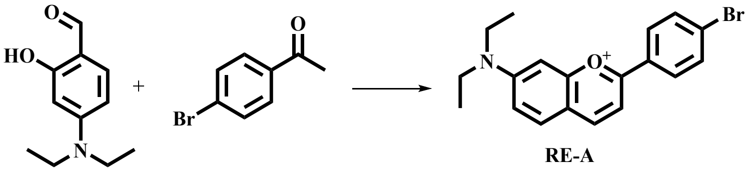

Scheme S1. Synthetic route of probe **RE-A**.

Synthesis of Probe **RE-A**. 4-(diethylamino) salicylaldehyde (193 mg, 1 mmol) was dissolved in 15 mL of concentrated sulfuric acid, and 4-bromoacetophenone (199 mg, 1 mmol) was gradually added during stirring. Then the temperature was raised to 85 °C and stirred for 6 h. After the reaction, the reaction solution is poured into 50 mL of ice water to quench, and about 0.5 mL of perchloric acid was added to precipitate the product as a black solid, which was filtered to provide crude **RE-A**. The crude residue was then purified using a silica gel column (DCM: MeOH = 70:1) to obtain **RE-A** as a gray-green solid product (193 mg, 54 % yield). The synthetic procedure is shown in **Scheme S1**. ^{1}H NMR (600 MHz, DMSO-d6) δ 8.81 (d, J = 7.9 Hz, 1H), 8.28 – 8.24 (m, 2H), 8.14 (d, J = 7.9 Hz, 1H), 8.03 (d, J = 9.5 Hz, 1H), 7.92 – 7.87 (m, 2H), 7.57 (dd, J = 9.5, 2.4 Hz, 1H), 7.40 (d, J = 2.3 Hz, 1H), 3.75 (s, 4H), 1.27 (t, J = 7.0 Hz, 6H). ^{13}C NMR (151 MHz, DMSO-d6) δ 164.53, 159.81, 157.16, 149.30, 133.17, 129.86, 129.40, 128.61, 120.55, 119.73, 109.80, 96.43, 46.41, 13.49, 12.50. HRMS (ESI): m/z calculated for [**RE-A**]$^{+}$

$C_{19}H_{19}BrNO^{+}$: 356.0645 , found: 356.0652.

4. Synthesis and characterization of RE-B.

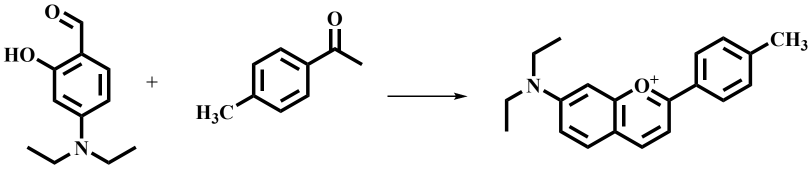

Scheme S2. Synthetic route for probe **RE-B**

Synthesis of Probe **RE-B**. 4-(diethylamino) salicylaldehyde (193 mg, 1 mmol) was dissolved in 15 mL of concentrated sulfuric acid, and 4-methylacetophenone (134 mg, 1 mmol) was gradually added during stirring. Then the temperature was raised to 85 °C and stirred for 6 h. After the reaction, the reaction solution is poured into 50 mL of ice water to quench, and about 0.5 mL of perchloric acid was added to precipitate the product as a black solid, which was filtered to provide crude **RE-B**. The residue was then purified using a silica gel column (DCM: MeOH = 50:1) to obtain **RE-B** as a gray-green solid product (196 mg, 67 % yield). The synthetic procedure is shown in **Scheme S2**. ^{1}H NMR (500 MHz, DMSO-d6) δ 8.80 (d, J = 8.0 Hz, 1H), 8.25 (d, J = 8.4 Hz, 2H), 8.09 (d, J = 8.0 Hz, 1H), 8.01 (d, J = 9.4 Hz, 1H), 7.55 – 7.47 (m, 3H), 7.38 (d, J = 2.4 Hz, 1H), 3.73 (q, J = 7.1 Hz, 4H), 2.45 (s, 3H), 1.26 (t, J = 7.0 Hz, 6H). ^{13}C NMR (126 MHz, DMSO-d6) δ 165.71, 159.18, 156.33, 149.06, 145.22, 132.49, 130.28, 127.79, 126.88, 119.23, 118.67, 108.81, 95.89, 45.67, 21.35. HRMS (ESI): m/z calculated for [**RE-B**]$^{+}$. $C_{20}H_{22}NO^{+}$: 292.1696, found: 292.1705.

5. Synthesis and characterization of RE-C.

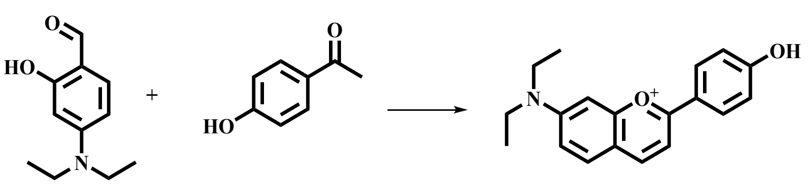

Scheme S3. Synthetic route of probe **RE-C**

Synthesis of Probe **RE-C**. 4-(diethylamino) salicylaldehyde (193 mg, 1 mmol) was dissolved in 15 mL of concentrated sulfuric acid, and 4-hydroxyacetophenone (136 mg, 1 mmol) was gradually added during stirring. Then the temperature was raised to 85 °C and stirred for 6 h.

After the reaction, the reaction solution was poured into 50 mL of ice water to quench, and about 0.5 mL of perchloric acid was added to precipitate the product as a black solid, which was filtered to prove crude **RE-C**. The residue was then purified using a silica gel column (DCM: MeOH = 50:1) to obtain **RE-C** as a gray-green solid product (150 mg, 51 % yield). The synthetic procedure is shown in **Scheme S3**. ^{1}H NMR (500 MHz, DMSO) δ 11.04 (s, 1H), 8.73 (d, J = 8.2 Hz, 1H), 8.30 – 8.24 (m, 2H), 7.96 (dd, J = 8.8, 7.8 Hz, 2H), 7.44 (dd, J = 9.4, 2.4 Hz, 1H), 7.31 (d, J = 2.3 Hz, 1H), 7.06 – 7.00 (m, 2H), 3.69 (q, J = 7.1 Hz, 4H), 1.24 (t, J = 7.1 Hz, 6H). ^{13}C NMR (126 MHz, DMSO) δ 166.75, 163.97, 158.73, 155.73, 148.96, 132.18, 130.88, 120.19, 117.72, 117.69, 116.80, 108.12, 95.85, 45.45, 12.43. HRMS (ESI): m/z calculated for [**RE-C**]$^{+}$. $C_{20}H_{22}NO^{+}$: 294.1489, found: 294.1498.

6. Synthesis and characterization of RE-D.

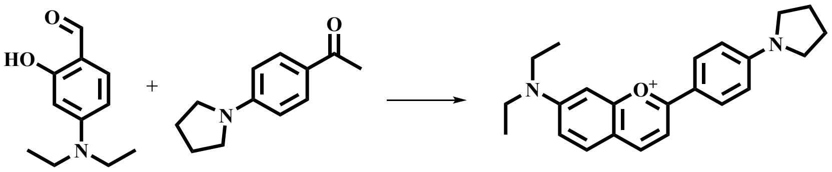

RE-D

Scheme S4. Synthetic route of probe **RE-D**

Synthesis of Probe **RE-D**. 4-(diethylamino) salicylaldehyde (193.25 mg, 1 mmol) was dissolved in 15 mL of concentrated sulfuric acid, and 1-(4-(pyrrolidine-1-yl) phenyl) acetonone (189.25 mg, 1 mmol) was gradually added during stirring. Then the temperature was raised to 85 °C and stirred for 6 h. After the reaction, the reaction solution was poured into 50 mL of ice water to quench, and about 0.5 mL of perchloric acid is added to precipitate the crude product as a black solid, which was filtered to provide crude **RE-D**. The residue was then purified using a silica gel column (DCM: MeOH = 50:1) to obtain **RE-D** as a gray-green solid product (205 mg, 59 % yield). The synthetic procedure is shown in **Scheme S4**. ^{1}H NMR (500 MHz, DMSO-d6) δ 8.47 (d, J = 8.6 Hz, 1H), 8.21 – 8.15 (m, 2H), 7.82 (t, J = 8.8 Hz, 2H), 7.24 (dd, J = 9.2, 2.4 Hz, 1H), 7.17 (d, J = 2.4 Hz, 1H), 6.78 – 6.71 (m, 2H), 3.62 (q, J = 7.0 Hz, 4H), 3.50 – 3.43 (m, 4H), 2.05 – 1.98 (m, 4H), 1.22 (t, J = 7.0 Hz, 6H). ^{13}C NMR (126 MHz, DMSO-d6) δ 167.95, 158.14, 154.85, 152.72, 147.33, 131.86, 131.55, 115.96, 115.68, 115.10, 113.51, 108.54, 96.46, 48.41, 45.48, 25.26, 12.90. HRMS (ESI): m/z calculated for [**RE-D**]$^{+}$. $C_{23}H_{27}N_{2}O^{+}$: 347.2118, found: 347.2125.

7. Synthesis and characterization of RF-1 and RF-2.

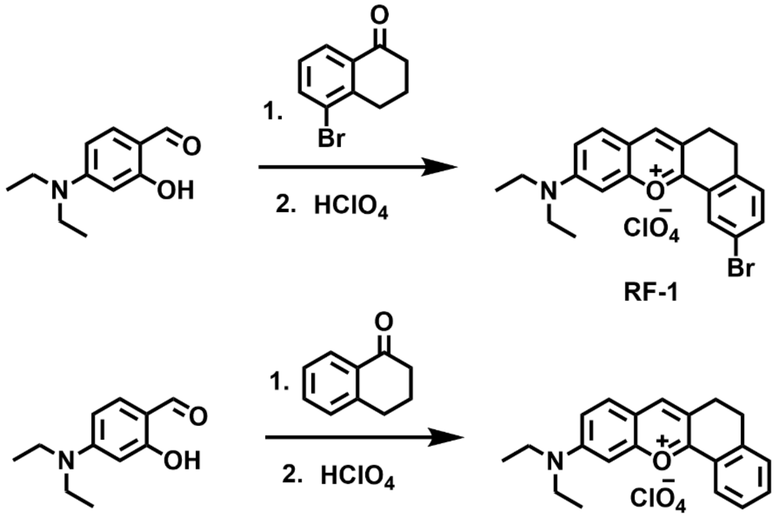

Scheme S5. Synthetic route of probe **RF-1** and **RF-2**

Synthesis of Probe **RF-1**: 4-(diethylamino) salicylaldehyde (1.93 g, 10 mmol) was suspended in 15 mL of concentrated sulfuric acid, followed by the slow addition of 5-bromo-1-tetralone (2.25 g, 10 mmol). The mixture was stirred at 100 °C for 6 hours until the reaction was complete. After cooling, the reaction mixture was slowly added dropwise into 100 mL of ice water; 1 mL of perchloric acid was then added, resulting in the precipitation of a black solid. The solid was collected by filtration to yield the crude product **RF-1**. The crude product was purified via silica gel column chromatography (DCM: MeOH = 50: 1) to afford pure **RF-1** as a gray-green solid (2.57 g, 67% yield). ^{1}H NMR (500 MHz, DMSO-d6) δ 8.74 (s, 1H), 8.38 (d, J = 2.1 Hz, 1H), 7.99 (d, J = 9.5 Hz, 1H), 7.82 (dd, J = 8.1, 2.1 Hz, 1H), 7.56 (dd, J = 9.5, 2.5 Hz, 1H), 7.47 (d, J = 1.7 Hz, 1H), 7.46 (d, J = 8.2 Hz, 1H), 3.75 (q, J = 7.0 Hz, 4H), 3.06 (s, 4H), 1.28 (t, J = 7.1 Hz, 6H). 13C NMR (126 MHz, DMSO) δ 160.19, 159.06, 156.57, 149.15, 140.85, 136.56, 132.69, 131.51, 128.86, 127.78, 121.91, 121.04, 120.47, 119.66, 96.36, 46.19, 26.09, 24.61, 13.12. HRMS (ESI): m/z calculated for [**RF-1**]$^+$: $C_{21}H_{21}BrNO^+$: 382.0802 found: 382.0811

Synthesis of Probe **RF-2**: 4-(diethylamino) salicylaldehyde (1.93 g, 10 mmol) was suspended in 15 mL of concentrated sulfuric acid, followed by the slow addition of 1-tetralone (1.46 g, 10 mmol). The mixture was stirred at 100 °C for 6 hours until the reaction was complete. After cooling, the reaction mixture was slowly added dropwise into 100 mL of ice-cold water, followed by the addition of 1 mL of perchloric acid; a black solid precipitated, which was collected by

filtration to yield the crude product **RF-2**. The crude product was purified via silica gel column chromatography (DCM:MeOH = 50:1) to afford pure **RF-2** as a gray-green solid (2.43 g, 80 % yield). ^{1}H NMR (500 MHz, DMSO-d6) δ 8.70 (s, 1H), 8.22 (d, J = 9.1 Hz, 1H), 7.96 (d, J = 9.5 Hz, 1H), 7.64 (td, J = 7.5, 1.3 Hz, 1H), 7.51 (ddd, J = 18.4, 13.4, 7.6 Hz, 3H), 7.33 (d, J = 2.4 Hz, 1H), 3.71 (q, J = 7.1 Hz, 4H), 3.07 (q, J = 3.6 Hz, 4H), 1.26 (t, J = 7.1 Hz, 6H). 13C NMR (126 MHz, DMSO) δ 162.27, 158.98, 156.21, 149.28, 142.03, 134.66, 132.56, 129.43, 128.27, 126.72, 125.92, 121.75, 119.73, 119.13, 96.08, 46.08, 26.56, 24.88, 12.95. HRMS (ESI): m/z calculated for [**RF-2**]$^+$: $C_{21}H_{22}NO^+$:304.1696 found:304.1704

8. Measurement of optical spectroscopic properties.

Unless otherwise noted, all the measurements were made according to the following procedure. The concentration of the probes stock solution was 1.0 mM in CH_3CN, and the analyte stock solutions were prepared in the deionized water at the appropriate concentration. The probe was added to a 10 mL color comparison tube. Probe was diluted to 10 μM in 10 mM PBS buffer (5% CH_3CN). The spectroscopic experiments were conducted using a 1 cm standard quartz cell at room temperature. The fluorescence intensity of probe **RE-A** was measured at **RE-A**: λ_{ex} = 545 nm, λ_{em} = 642 nm; **RE-B**: λ_{ex} = 545 nm, λ_{em} = 616 nm; **RE-C**: λ_{ex} = 594 nm, λ_{em} = 618 nm; **RE-D**: λ_{ex} = 615 nm, λ_{em} = 653 nm. The excitation slit widths were 10 nm, and the emission slit widths were 10 nm.

9. Detection limit of RE-A for SO_2

According to the absorption titration curve of **RE-A** in the presence of different concentrations of HSO_3^-, the detection limit can be calculated by the following equation (1):

$$\text{Detection limit} = 3\sigma/k \qquad (1)$$

Here, σ represents the standard deviation of the blank measurements, and k represents the slope of the curve of absorption intensity as a function of HSO_3^- concentrations. The absorption spectrum of the blank sample was measured eleven times to calculate the standard deviation.

10. Culture and preparation of the HepG2 cells

The HepG2 cells were cultured in DMEM (Dulbecco's modified Eagle's medium) supplemented with 10% FBS (fetal bovine serum) in an atmosphere of 5% CO_2 and 95% air at

37 °C. Before the experiments, seed the HepG2 cells in 35-mm glass-bottomed dishes at a density of 2×10^5 cells per dish in 2 mL of culture medium and incubate them inside an incubator containing 5% CO_2 and 95% air at 37 °C. Incubate the cells for 24 h. Cells will attach to the glass surface during this time.

11. Cytotoxicity assay.

With this research, the cytotoxicity of **RE-D** in living HepG2 cells was investigated by MTT assay. HepG2 cells were seeded at a density of 50000 cells/mL in a 96-well micro-assay culture plate and growth for 24 h in a 5 % CO_2/95 % air incubator. The cell Dulbecco's Modified Eagle Medium (DMEM) culture medium of each well was then replaced with the fresh medium containing increasing concentrations of **RE-D**, i.e., 0, 10, 20, 30, 40, and 50 μM. The wells with the cell culture medium only were employed as the blank. After incubation in a 5 % CO_2/95% air incubator for 24 h, cell culture medium was removed and the cells were washed three times with PBS. Then, 100 μL of 0.5 mg/mL MTT solution in PBS was added to each well, and the cells were incubated for another 4 h. The excess MTT solution was then carefully removed from each well, and the formed formazan was dissolved in 100 μL of DMSO. The optical density of each well was measured at a wavelength of 490 nm using a microplate reader (Bio-Tek, USA). The results from the five individual experiments were averaged. The following formula was used to calculate the viability of cell growth: Viability = (mean of absorbance value of treatment group– blank) / (mean absorbance value of control – blank) × 100%. All of the measurements were performed five times and the values are presented as the mean ± SD.

12. FL imaging of probe RE-D in live mice

The mice (4-5 weeks) were purchased from the Experimental Animal Center of the Guangxi Medical University (Nanning, China). All animal experiments were reviewed and approved by the Animal Care and Experiment Committee of Guangxi University (protocol number: Gxu-2024-120).

Before in vivo imaging, hair was removed by an electric shaver and then, the mice were anesthetized by a 4 % chloral hydrate aqueous solution. Fluorescence imaging was done at Guangxi University. (Small Animal Imaging System, IVIS Lumina III and USA). The excitation

wavelength for all fluorescence images was 600 nm and the emission wavelength was 670 nm.

13. **Preparation and determination of real samples.**

All Chinese medicinal materials (yam, kudzu root, poria cocos, and angelica dahurica) are first pulverized and passed through a 65-mesh stainless steel sieve, then sealed and stored away from light. Accurately weigh 1.5 g of powder and place it in a 50 mL stoppered centrifuge tube. Add 10 mL of PBS buffer (10 mM, pH 7.4) containing 5% acetonitrile, vortex to mix, and then sonicate in a 25 ℃ constant temperature water bath for 2 hours at a sonication power of 300 W and a frequency of 40 kHz. After the extract cools to room temperature, dilute it several times with the above PBS-acetonitrile buffer, and then filter it through a 0.22 μm mixed cellulose ester microporous membrane. During filtration, strictly discard approximately 1–2 mL of the initial filtrate, collecting only the subsequent filtrate for subsequent detection, thereby avoiding interference caused by the adsorption of the target analyte by the filter membrane. For nail polish samples, the same procedure was followed: accurately weigh an appropriate amount of viscous nail polish, disperse it in an equal volume of extraction solvent, sonicate for 2 hours, and then filter before analysis. All samples were prepared in parallel (n = 6).

14. Matrix effect assessment

Matrix effect assessment was conducted using yam as a representative matrix. A series of sulfite standard solutions with varying concentrations were prepared using yam blank matrix extract as the solvent. After adding probe **RE-D**, the absorbance difference ΔA was measured, and a matrix-matched standard curve was plotted. Simultaneously, sulfite standard solutions with the same concentration gradient were prepared using pure PBS-acetonitrile buffer as the solvent, and a pure solvent standard curve was plotted using the same method. The matrix effect factor ME % was calculated using the following formula:

$$\text{ME}\ \% = \frac{k_{\text{matrix}}}{k_{\text{solvent}}} \times 100\%$$

k_{matrix}= slope of the matrix-matched calibration curve

k_{solvent}= slope of the neat solvent calibration curve

The matrix effect of formaldehyde detection in nail polish matrix was evaluated using the same method.

15. Limit of Quantification

The limit of quantitation (LOQ) was determined using a yam blank matrix as a representative sample. Ten spiked samples were prepared independently from the yam blank matrix extract at a low concentration level close to the expected LOQ. An equal amount of probe **RE-D** was added to each sample. After mixing, the samples were allowed to stand until the signal stabilized, and the absorbance difference (A) was measured. The standard deviation (SD) of the ten response values was calculated, and the LOQ in the solution was calculated using the following formula:

$$\mathrm{LOQ}_{\mathrm{solution}} = \frac{10 \times \mathrm{SD}}{a}$$

Where 'a' represents the slope of the calibration curve using the yam blank matrix standard addition method. The limit of quantitation in the resulting solution is converted to the limit of quantitation (mg/kg) in the medicinal material based on the dilution factor during pretreatment.

16. Preparation of encrypted ink.

Mix 1 mL of ethanol, 1 mL of dichloromethane, and 1 mL of glycerol separately, and then add 100 μL of **RE-A** solution (10 mM) to prepare Ink A. Similarly, mix 1 mL of ethanol, 1 mL of dichloromethane, and 1 mL of glycerol separately, and then add 100 μL of **RE-D** solution (10 mM) to prepare Ink D.

Supplementary table

Table S1. Basic parameters of hole-electron analysis.

Compound	Transition	D[a] (Å)	Sr[b] (a.u.)	t[c] (Å)	Δσ[d] (Å)	HDI[e]	EDI[f]	Type[g]
**RE-A**	$S_0 \rightarrow S_1$	1.898	0.66751	-0.671	-0.573	8.69	8.30	HLCT
**RE-B**	$S_0 \rightarrow S_1$	1.951	0.66453	-0.664	-0.423	8.71	8.44	HLCT
**RE-C**	$S_0 \rightarrow S_1$	1.570	0.65449	-1.054	-0.703	8.20	8.47	LE
**RE-D**	$S_0 \rightarrow S_1$	1.281	0.63774	-1.254	-1.140	7.50	8.19	LE

[a] distance between centroid of hole and electron.
[b] overlap function between hole and electron distribution.
[c] separation degree of hole and electron in CT direction.
[d] distribution breadth difference index of electrons and holes.
[e] hole delocalization index.
[f] electron delocalization index.
[g] HLCT=hybrid local and charge transfer, LE=local excitation.

Table S2. Percentage contribution of each fragment to hole and electron.

Compound	Transition	Fragment	Hole[a] (%)	Electron[b] (%)	Overlap[c] (%)	Diff[d] (%)
**RE-A**	$S_0 \rightarrow S_1$	1	31.56	5.14	12.74	-26.41
		2	52.89	74.40	62.73	21.51
		3	12.11	19.04	15.19	6.93
		4	3.44	1.42	2.21	-2.03
**RE-B**	$S_0 \rightarrow S_1$	1	31.73	5.03	12.64	-26.70
		2	53.10	75.59	63.35	22.49
		3	14.21	18.56	16.24	4.34
		4	0.96	0.82	0.88	-0.14
**RE-C**	$S_0 \rightarrow S_1$	1	28.93	4.99	12.01	-23.94
		2	49.08	75.30	60.79	26.22
		3	18.36	17.68	18.02	-0.67
		4	3.64	2.03	2.72	-1.61
**RE-D**	$S_0 \rightarrow S_1$	1	17.03	4.64	8.89	-12.39
		2	35.35	71.29	50.20	35.94

	3	28.29	18.07	22.61	-10.22
	4	18.58	5.66	10.26	-12.92

[a] percentage contribution of each fragment to the hole.

[b] percentage contribution of each fragment to electron.

[c] percentage overlap of hole and electron in each fragment.

[d] difference in percentage contribution of hole and electron from each fragment.

Table S3. Net amount (e) of electron transfer between fragments.

Compound	Transition	1→2	1→3	1→4	2→3	2→4	3→4	CT[a] (%)	LE[b] (%)
**RE-A**	$S_0 \rightarrow S_1$	0.208	0.054	0.003	0.010	-0.018	-0.005	56.67	43.33
**RE-B**	$S_0 \rightarrow S_1$	0.213	0.052	0.002	-0.009	-0.002	-0.001	55.62	44.38
**RE-C**	$S_0 \rightarrow S_1$	0.193	0.042	0.004	-0.051	-0.012	-0.003	58.28	41.72
**RE-D**	$S_0 \rightarrow S_1$	0.105	0.018	0.001	-0.138	-0.112	-0.018	66.77	32.16

[a] intrinsic charge transfer percentage, CT=charge transfer.

[b] intrinsic local excitation percentage, LE=local excitation

Table S4. Calculation values of each parameter for the electron imbalance degree D in Reaction 1 and Reaction 2.

	**RE-A**	**RE-B**	**RE-C**	**RE-D**	**RE-A-SO_3H**	**RE-B-SO_3H**	**RE-C-SO_3H**	**RE-D-SO_3H**	HSO_3^-	HCHO	HCHO-HSO_3^-
σ_{tot} (kcal/mol)	15.318	10.762	12.003	7.580	15.951	15.830	16.707	16.819	17.755	12.175	24.029
V_{max} (kcal/mol)	95.221	91.706	109.891	84.101	59.654	48.085	60.350	52.870	-65.020	27.836	-61.499
V_{min} (kcal/mol)	30.358	45.771	24.139	45.252	-49.433	-48.986	-48.748	-56.358	-150.826	-34.478	-138.717
ΔV (kcal/mol)	64.863	45.935	85.751	38.848	109.087	97.071	109.098	109.228	85.806	62.314	77.218
$2\sigma_{tot}/\Delta V$	0.472	0.469	0.280	0.390	0.292	0.326	0.306	0.308	0.414	0.391	0.622
$2\sigma_{tot}^2/\Delta V$ (kcal/mol)	7.235	5.043	3.360	2.958	4.665	5.163	5.117	5.180	7.347	4.757	14.955
S+/S- (C)	3.310	3.542	4.044	4.773	1.815	2.089	2.256	3.087	-	1.767	4.159
S-/S+ (C)	0.302	0.282	0.247	0.210	0.551	0.479	0.443	0.324	-	0.566	0.240
S+/S- (S)	-	-	-	-	1.504	1.489	1.594	3.031	0.556	-	0.498
S-/S+ (S)	-	-	-	-	0.665	0.672	0.628	0.330	1.798	-	2.008
D[a] (kcal/mol)	23.945	17.862	13.589	14.118	9.586	10.157	10.423	17.379	13.212	-	-
D[b] (kcal/mol)	2.186	1.424	0.831	0.620	11.565	14.254	14.753	17.697	-	8.405	11.041

[a] **Reaction 1: RE-X** + HSO_3^- → **RE-X-SO_3H**

[b] **Reaction 2: RE-X-SO_3H** + HCHO → **RE-X** + HCHO-HSO_3^-

Table S5. ΔG_r calculated at three computational levels for Reaction 1: **RE-X** + HSO_3^- → **RE-X-SO₃H**.

	level	RE-A	RE-B	RE-C	RE-D
G_R (kcal/mol)	M062X[a]	-2550050.867	-959738.036	-982295.673	-1067671.827
G_P (kcal/mol)		-2550059.084	-959744.544	-982301.802	-1067671.580
ΔG_r (kcal/mol)		-8.217	-6.508	-6.129	0.247
G_R (kcal/mol)	ωB97M-V[b]	-2549969.726	-959766.319	-982331.725	-1067699.964
G_P (kcal/mol)		-2549980.091	-959774.913	-982339.928	-1067702.329
ΔG_r (kcal/mol)		-10.364	-8.594	-8.202	-2.365
G_R (kcal/mol)	DLPNO-CCSD(T)[c]	-2547698.117	-958278.604	-980815.213	-1066028.100
G_P (kcal/mol)		-2547709.948	-958288.235	-980824.771	-1066031.902
ΔG_r (kcal/mol)		-11.831	-9.631	-9.558	-3.802

[a] High-precision gas-phase single-point energy calculations were performed using Gaussian 16 at the M062X level with D3 dispersion correction and the Def2-TZVP basis set.

[b] High-precision gas-phase single-point energy calculations were performed using ORCA 6.0.1 at the ωB97M-V level with the Def2-TZVP basis set, Def2/J auxiliary basis set, and the RIJCOSX approximation.

[c] High-precision gas-phase single-point energy calculations were performed using ORCA 6.0.1 at the DLPNO-CCSD(T) level with normalPNO settings, the def2-TZVPP basis set, Def2/J and def2-TZVPP/C auxiliary basis sets, RIJCOSX approximation, and tightSCF convergence criteria.

Table S6. ΔG_r calculated at three computational levels for Reaction 2: **RE-X-SO₃H** + HCHO → **RE-X** + HCHO-HSO_3^-.

	level	RE-A	RE-B	RE-C	RE-D
G_R (kcal/mol)	M062X[a]	-2621904.640	-1031590.101	-1054147.359	-1139517.136
G_P (kcal/mol)		-2621911.147	-1031598.316	-1054155.953	-1139532.107
ΔG_r (kcal/mol)		-6.506	-8.215	-8.594	-14.970
G_R (kcal/mol)	ωB97M-V[b]	-2621831.117	-1031625.939	-1054190.954	-1139553.356
G_P (kcal/mol)		-2621834.676	-1031631.269	-1054196.675	-1139564.914
ΔG_r (kcal/mol)		-3.559	-5.329	-5.721	-11.558
G_R (kcal/mol)	DLPNO-CCSD(T)[c]	-2619453.849	-1030032.135	-1052568.671	-1137775.803
G_P (kcal/mol)		-2619454.889	-1030035.376	-1052571.984	-1137784.872
ΔG_r (kcal/mol)		-1.040	-3.240	-3.313	-9.069

[a] High-precision gas-phase single-point energy calculations were performed using Gaussian 16 at the M062X level with D3 dispersion correction and the Def2-TZVP basis set.

[b] High-precision gas-phase single-point energy calculations were performed using ORCA 6.0.1 at the ωB97M-V level with the Def2-TZVP basis set, Def2/J auxiliary basis set, and the RIJCOSX approximation.

[c] High-precision gas-phase single-point energy calculations were performed using ORCA 6.0.1 at the DLPNO-CCSD(T) level with normalPNO settings, the def2-TZVPP basis set, Def2/J and def2-TZVPP/C auxiliary basis sets, RIJCOSX approximation, and tightSCF convergence criteria.

Table R1 Calculation values of each parameter for the electron imbalance degree D in Reaction 1 and Reaction 2

Table S7. Calculation values of each parameter for the electron imbalance degree D in Reaction 1 and Reaction 2

	**RF-1**	**RF-2**	**RF-1-SO$_3$H**	**RF-2-SO$_3$H**	HSO_3^-	HCHO	HCHO-HSO_3^-
σ_{tot} (kcal/mol)	13.993	9.610	15.371	15.314	17.755	12.175	24.029
V_{max} (kcal/mol)	94.636	92.076	61.357	52.352	-65.020	27.836	-61.499
V_{min} (kcal/mol)	29.604	44.273	-49.892	-48.017	-150.826	-34.478	-138.717
ΔV (kcal/mol)	65.032	47.803	111.249	100.370	85.806	62.314	77.218
$2\sigma_{tot}/\Delta V$ (kcal/mol)	0.430	0.402	0.276	0.305	0.414	0.391	0.622
$2\sigma_{tot}^2/\Delta V$ (kcal/mol)	6.022	3.864	4.248	4.673	7.347	4.757	14.955
S+/S- (C)	4.024	4.204	2.226	2.433	-	1.767	4.159
S-/S+ (C)	0.249	0.238	0.449	0.411	-	0.566	0.240
S+/S- (S)	-	-	1.643	1.812	0.556	-	0.498
S-/S+ (S)	-	-	0.609	0.552	1.798	-	2.008
D^a (kcal/mol)	24.234	16.243	8.887	10.389	13.212	-	-
D^b (kcal/mol)	1.496	0.919	12.039	13.947	-	8.405	11.041

a Rreaction 1: **RF-X** + HSO_3^- → **RF-X-SO$_3$H**

b Rreaction 2: **RF-X-SO$_3$H** + HCHO → **RF-X** + HCHO-HSO_3^-

Table S8. The ΔD_r of Reaction 1: **RF-X** + HSO_3^- → **RF-X-SO$_3$H**

	**RF-1**	**RF-2**
D_R (kcal/mol)	37.446	29.455
D_P (kcal/mol)	8.887	10.389
ΔD_r (kcal/mol)	-28.558	-19.065

Table S9. The ΔD_r of Reaction 2: **RF-X-SO$_3$H** + HCHO → **RF-X** + HCHO-HSO_3^-

	**RF-1**	**RF-2**
D_R (kcal/mol)	20.444	22.353
D_P (kcal/mol)	12.537	11.960
ΔD_r (kcal/mol)	-7.907	-10.392

-

Table S10. The orbital transition type, transition orbital contribution and π component of **RE** probes under the transition ($S_0 \rightarrow S_1$).

Compound	Transition	Orbital transition	Contribution	π composition (HOMO)	π composition (LUMO)	Types
**RE-A**	$S_0 \rightarrow S_1$	H→L	98.9%	88.865%	90.610%	π→π*
**RE-D**	$S_0 \rightarrow S_1$	H→L	99.0%	93.055%	89.975%	π→π*

Table S11. Optical properties of dyes **RE** in DCE at 25 ℃.

**Dye**	**λ_{abs}[nm]**	**ε (×10^4 M^{-1} cm^{-1})**	**Φ [%]**
**RE-A**	525	2.22	2.9
**RE-D**	601	6.44	24.0

Table S12. The detection results of **RE-D** for HSO_3^- in traditional Chinese medicinal materials.

**Medicinal materials**	**Added HSO_3^- (μM)**	**Found HSO_3^- (μM)[a]**	**Recovery (%)**	**RSD (%)**
Chinese yam	0	0.27	—	5.11
	0.5	0.76	93.30	3.02
	0.8	1.03	93.15	2.85
	1	1.05	100.03	1.72
Kudzu powder	0	0.58	—	5.62
	0.5	1.10	104.92	2.14
	0.8	1.41	104.00	1.21
	1	1.52	94.47	1.82
Poria cocos	0	0.34	—	2.88
	0.5	0.82	96.53	2.39
	0.8	1.14	99.39	2.74
	1	1.29	95.49	2.63
Angelica Dahurica	0	0.89	—	1.99
	0.5	1.40	102.91	2.81
	0.8	1.68	98.68	3.47
	1	1.84	94.66	3.12

Table S13. The detection results of **RE-D-SO_3H** for HCHO in nail enamels.

Nail enamels	Added HCHO (μM)	Found HCHO (μM)[a]	Recovery (%)	RSD (%)
Nail enamel 297	0	122.37	—	2.06
	20	142.96	102.96	3.08
	40	161.48	97.78	2.20
	50	165.93	87.11	1.16
Nail enamel 301	0	63.00	—	4.13
	20	82.68	98.41	2.17
	40	102.93	99.82	2.12
	50	112.68	99.37	2.33
Nail enamel 299	0	62.08	—	3.19
	20	79.58	87.50	2.41
	40	100.42	95.83	4.26
	50	110.00	95.83	3.10
Nail enamel 300	0	67.68	—	5.28
	20	86.85	95.79	3.09
	40	104.74	92.63	2.42
	50	118.95	102.53	2.33

Table S14. Limits of quantification (LOQ), measured values by the proposed method, measured values by the pharmacopoeia method, and relative errors for sulfur dioxide in four Chinese medicinal materials

Medicinal materials	LOQ (mg/kg)	The method of RE-D (mg/kg)	National standard method (mg/kg)[a]	Relative error to national standard method (%)
Chinese yam	2.56	4.61	4.98	8.03
Kudzu powder	2.57	9.90	10.17	2.65
Poria cocos	1.08	5.80	6.01	3.49
Angelica Dahurica	1.71	15.19	16.31	6.87

[a] Chinese Medicine Pharmacopoeia 2020 Edition Four General Rules 2331, China Food and Drug Administration (CFDA).

Table S15. Limits of quantification (LOQ), measured values by the proposed method, measured values by the standard method, and relative errors for formaldehyde in four nail polish samples

Nail enamels	LOQ (mg/kg)	The method of RE-D-HSO_3 (mg/kg)	National standard method (mg/kg)[a]	Relative error to national standard method (%)
Nail polish 297	15.11	34.60	35.84	3.46
Nail polish 301	11.53	69.55	72.92	4.62
Nail polish 299	18.00	77.58	80,13	3.18
Nail polish 300	32.58	164.05	180.01	8.87

Supplementary figures

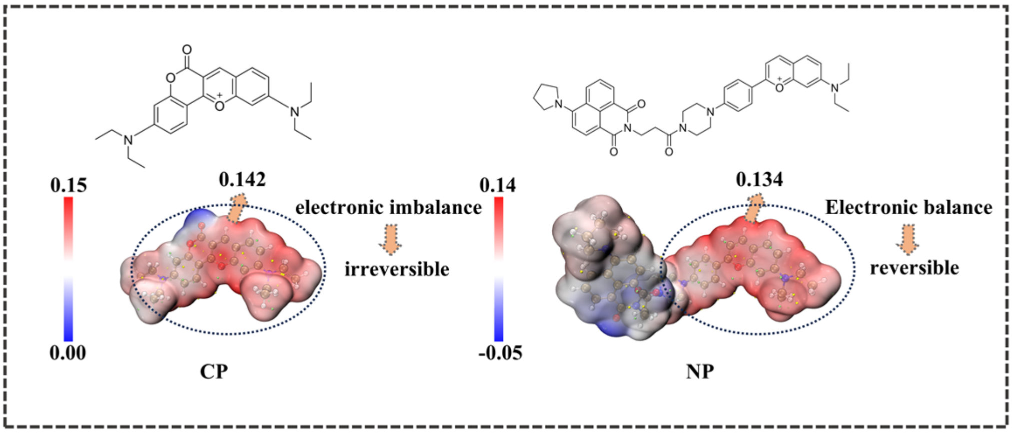

Figure S1. Analysis of electrostatic potential and reversibility of probes **CP** and **NP**.

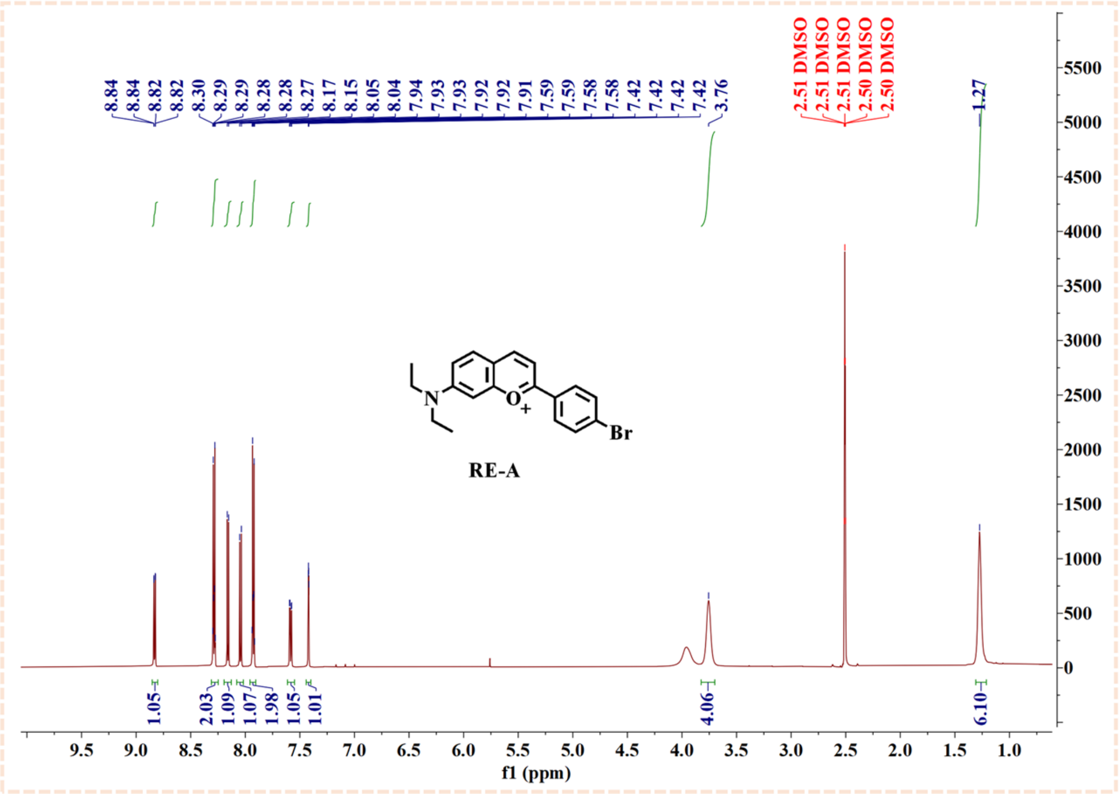

Figure S2. The ^{1}H NMR of **RE-A** in DMSO-d_6.

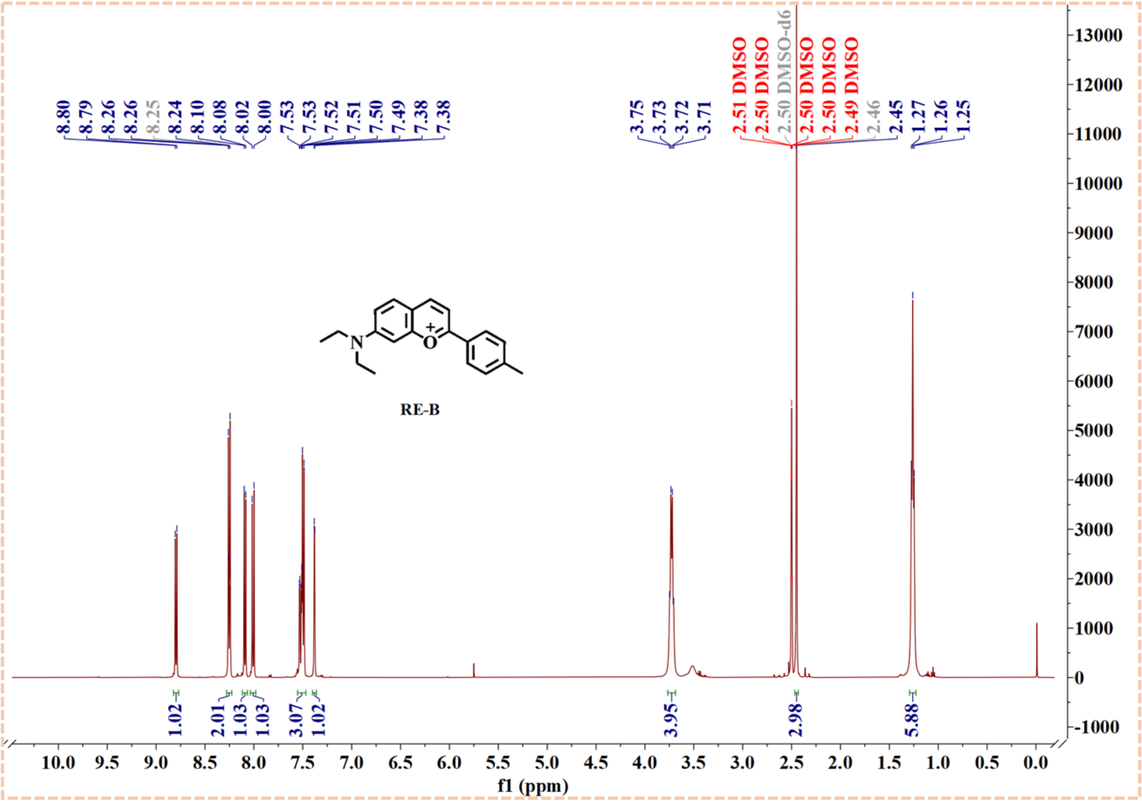

Figure S3. The ^{1}H NMR of **RE-B** in DMSO-d_6**.**

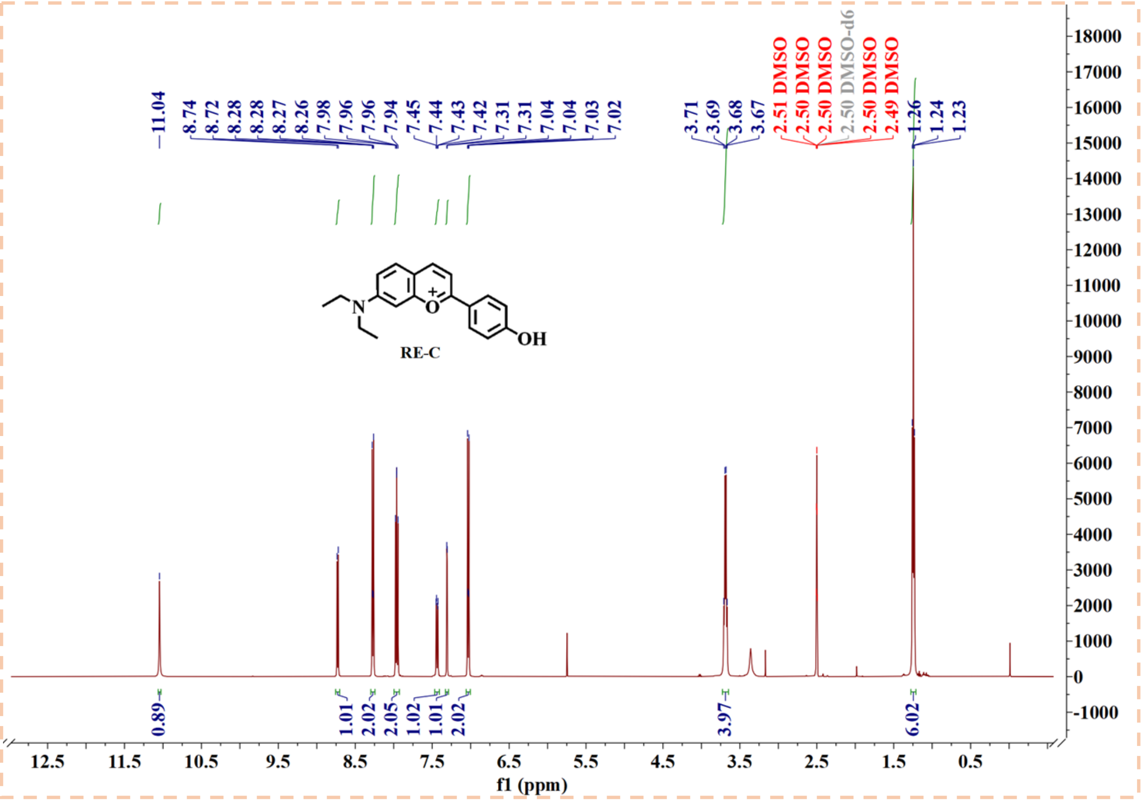

Figure S4. The ^{1}H NMR of **RE-C** in DMSO-d_6.

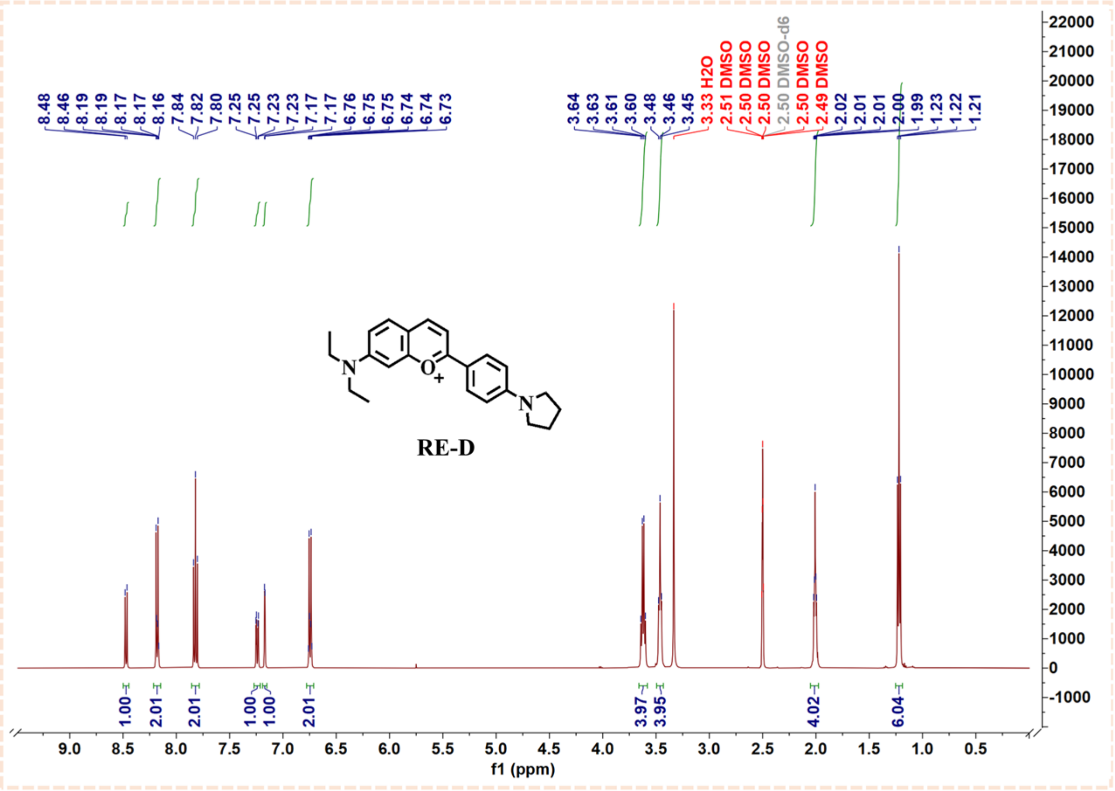

Figure S5. The ^{1}H NMR of **RE-D** in DMSO-d_6.

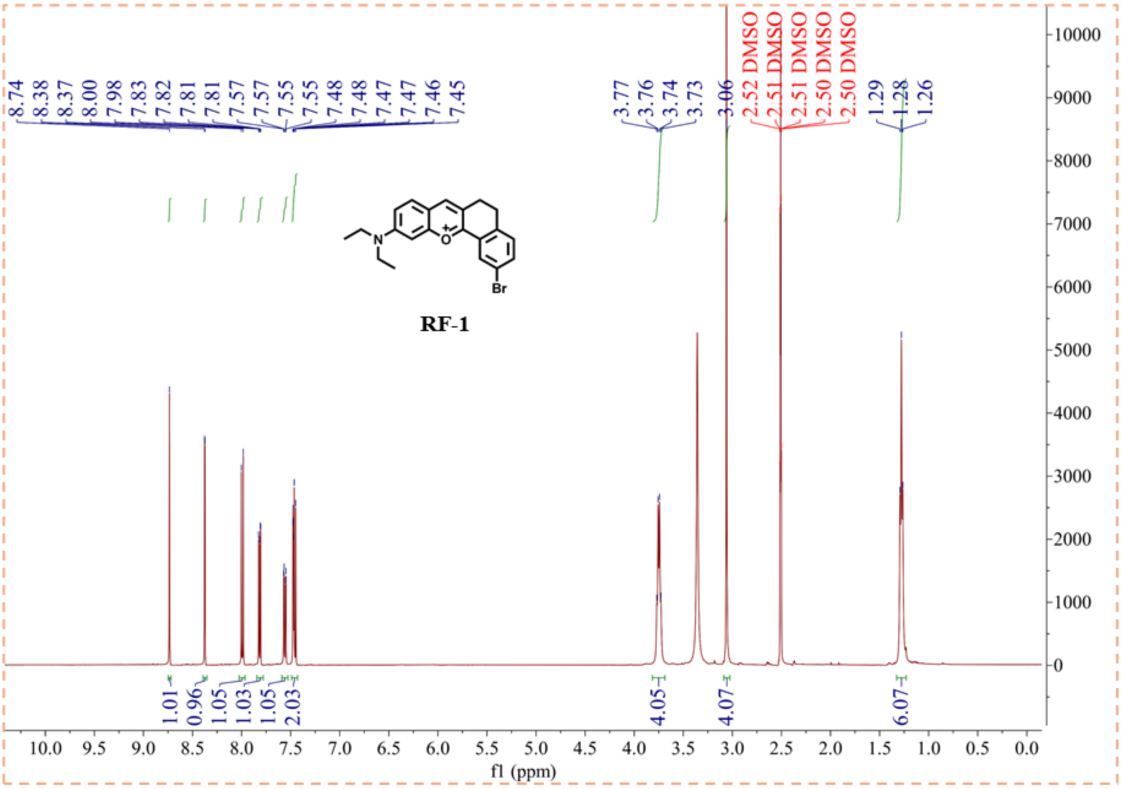

Figure S6. The ^{1}H NMR of **RF-1** in DMSO-d_6.

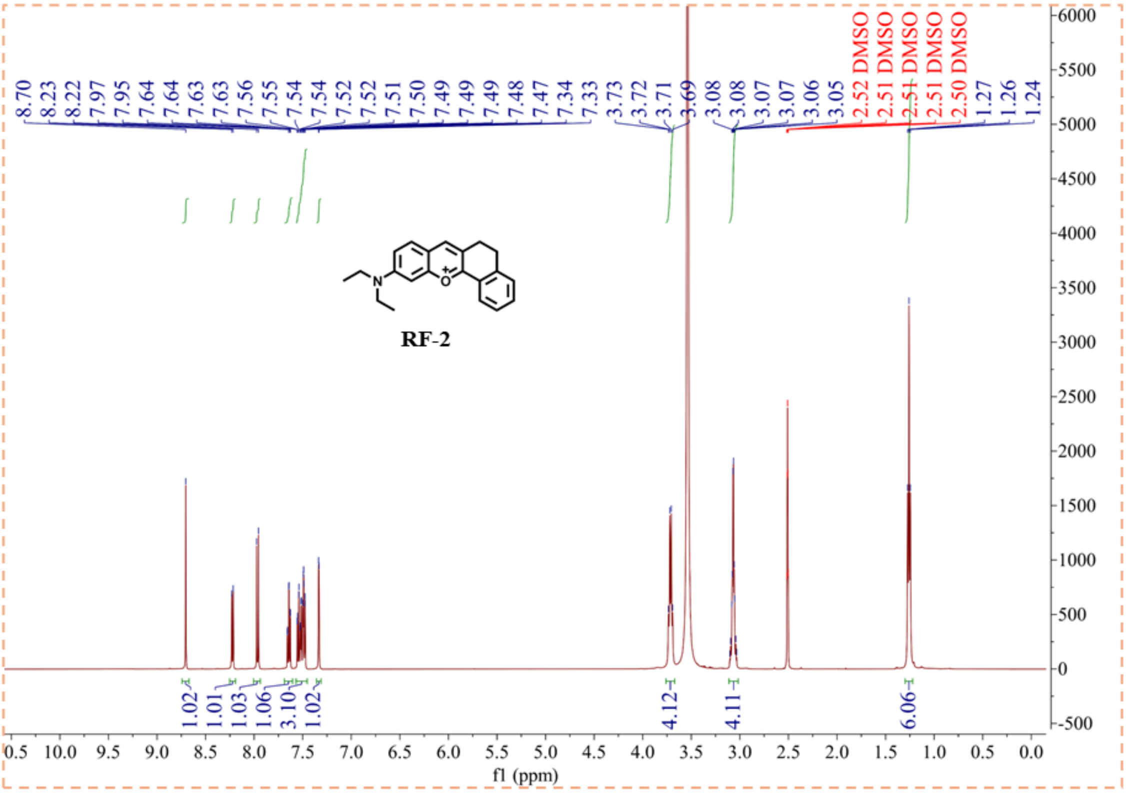

Figure S7. The ^{1}H NMR of **RF-2** in DMSO-d6.

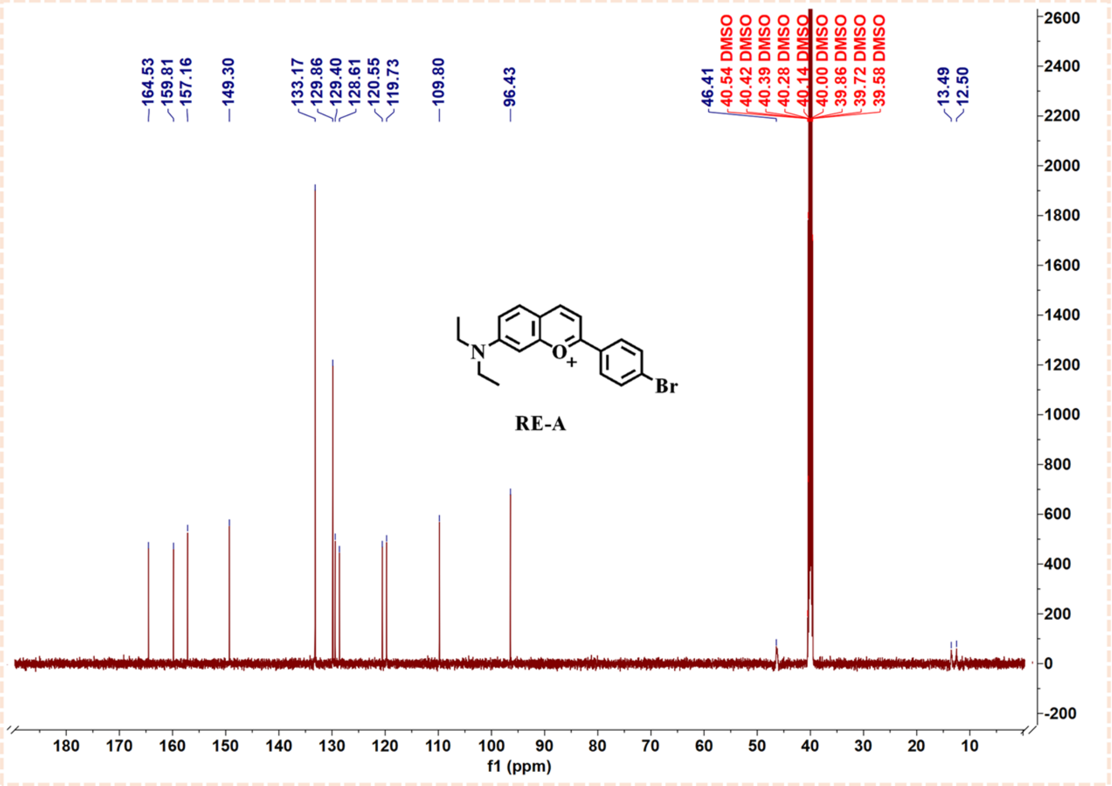

Figure S8. The ^{13}C NMR of **RE-A** in DMSO-d_6.

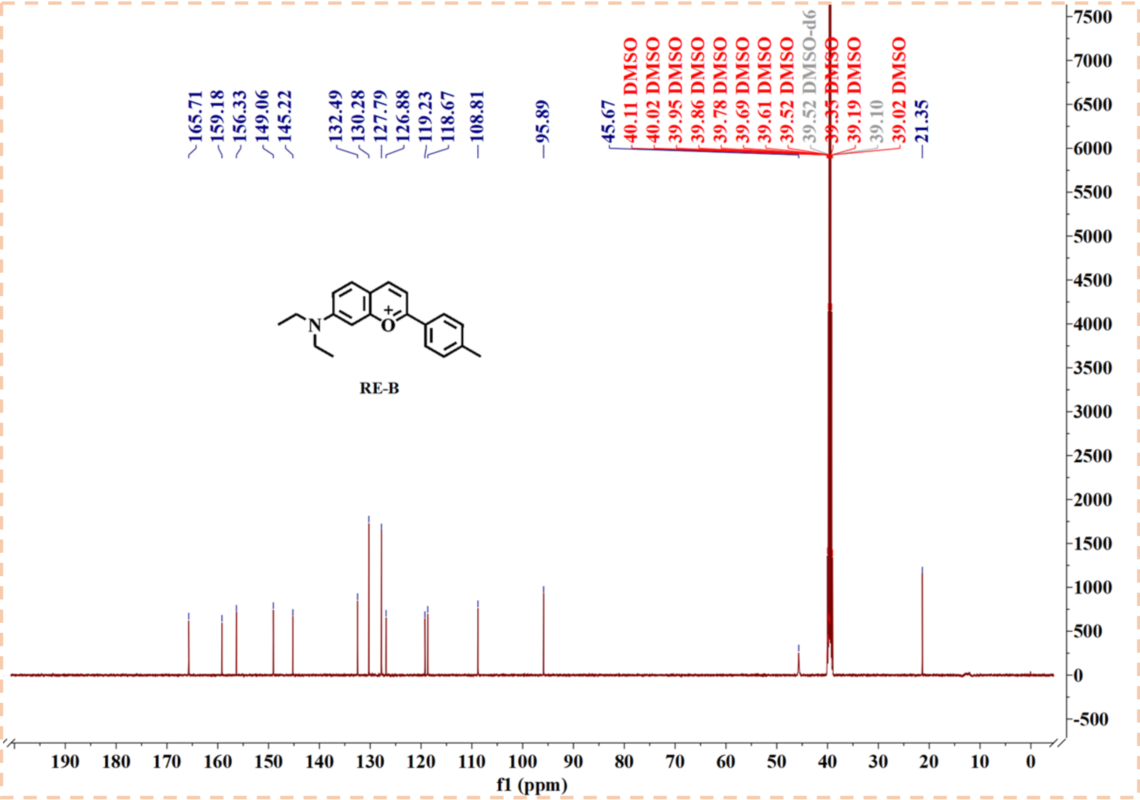

Figure S9 The ^{13}C NMR of **RE-B** in DMSO-d_6.

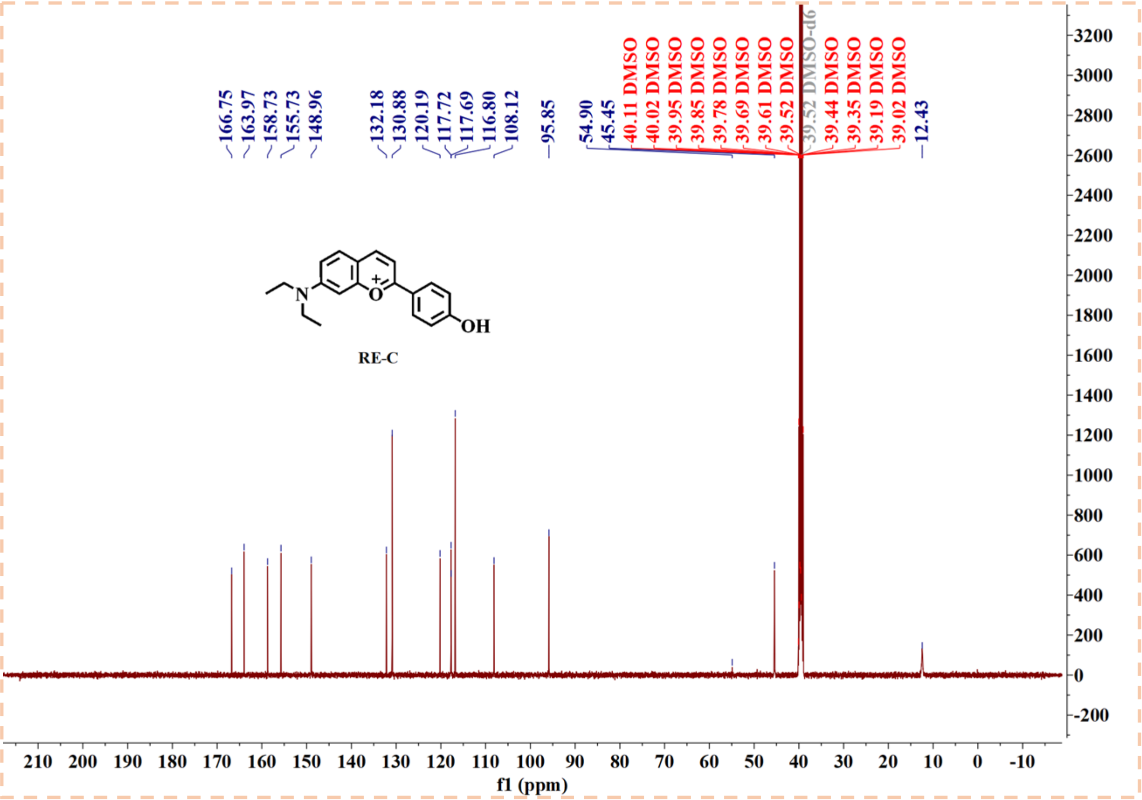

Figure S10. The ^{13}C NMR of **RE-C** in DMSO-d_6.

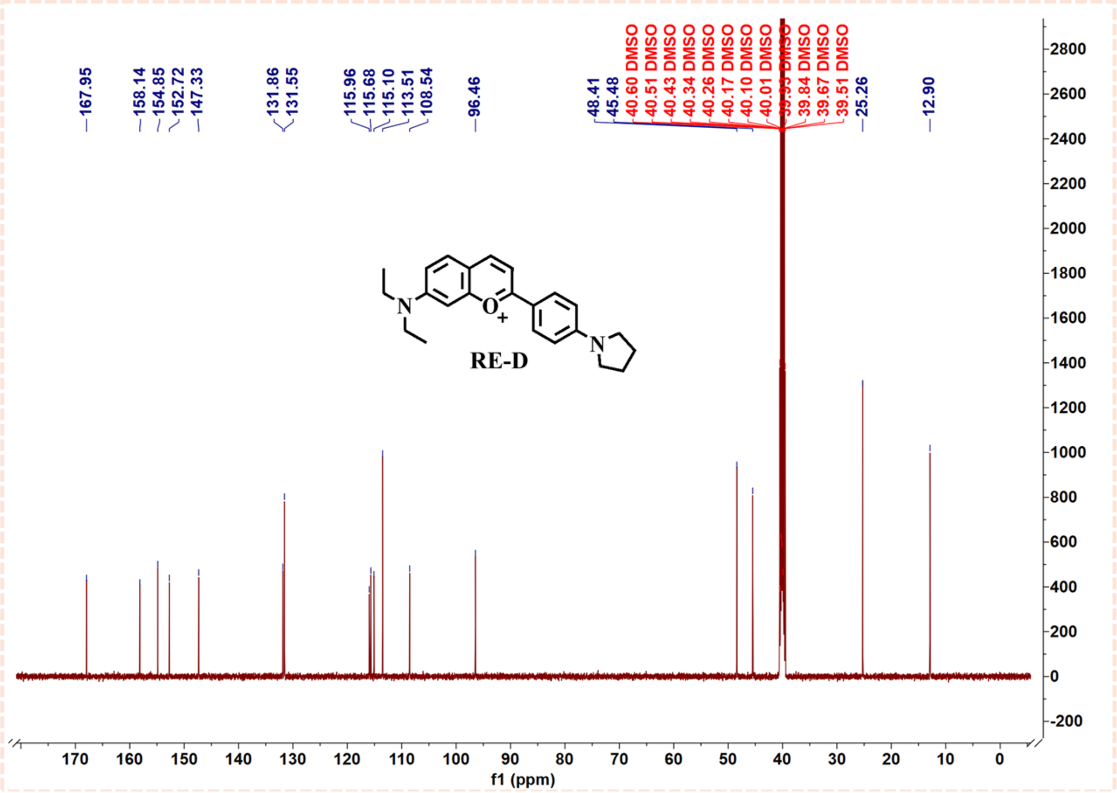

Figure S11. The ^{13}C NMR of **RE-D** in DMSO-d_6.

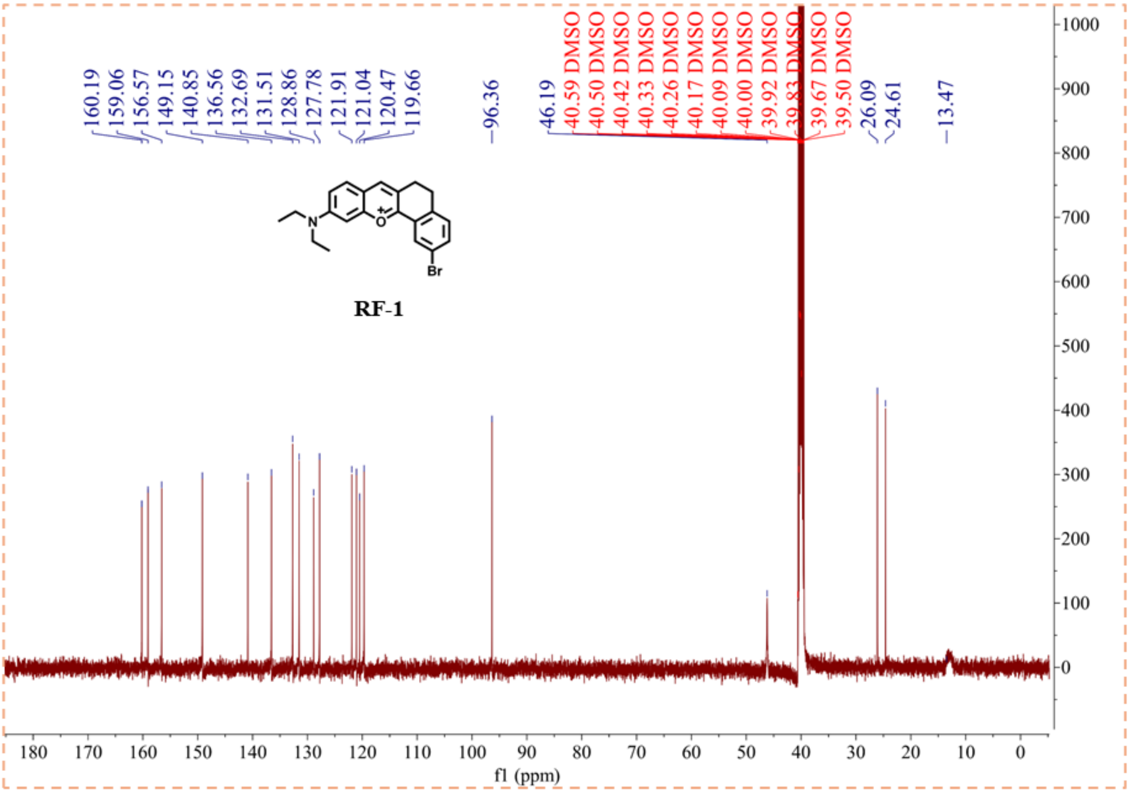

Figure S12. The ^{13}C NMR of **RF-1** in DMSO-d_6.

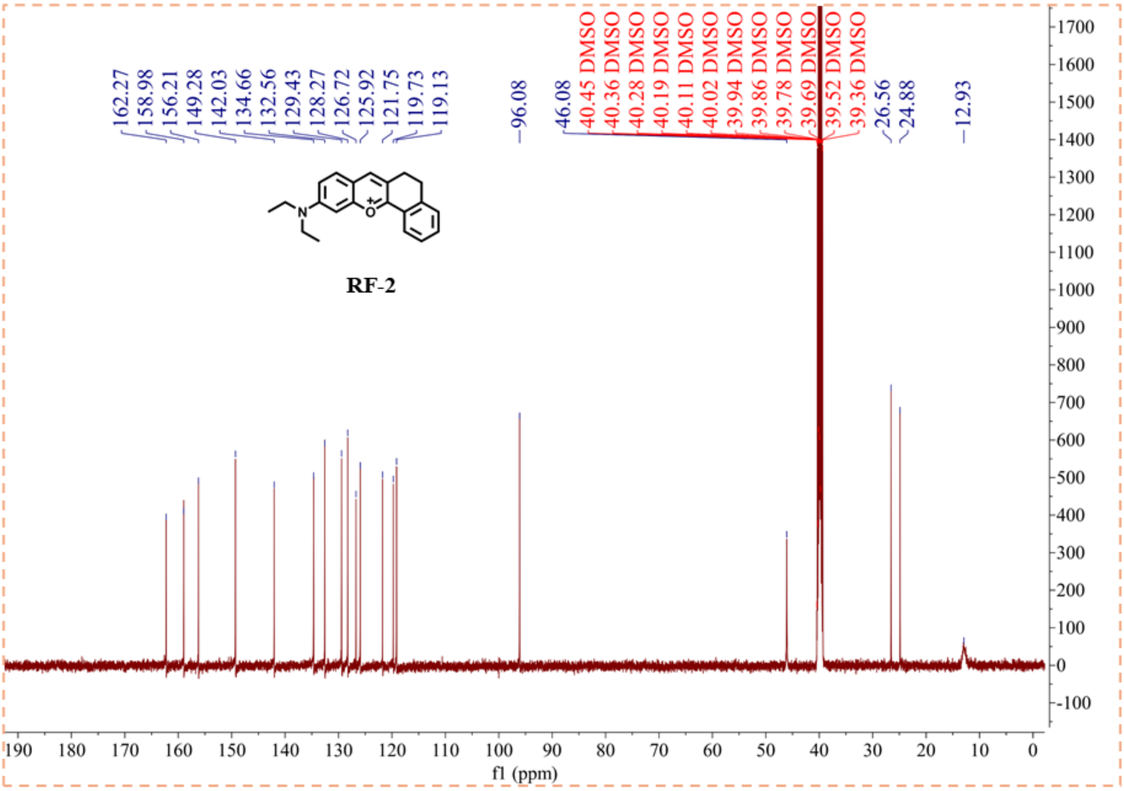

Figure S13. The ^{13}C NMR of **RF-2** in DMSO-d6

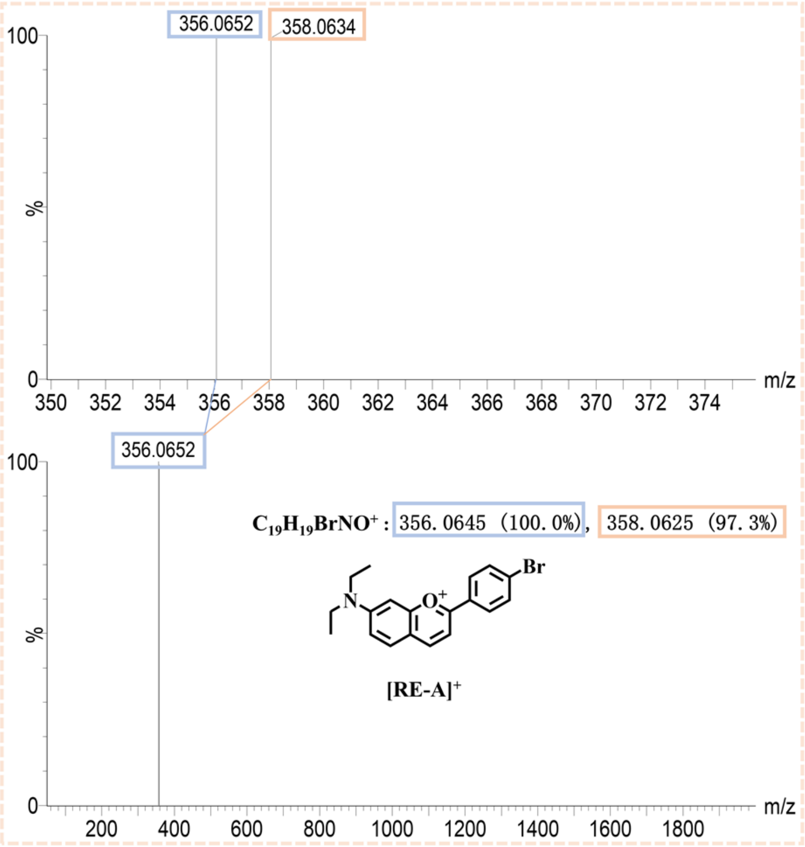

Figure S14. HRMS spectrum of **RE-A**.

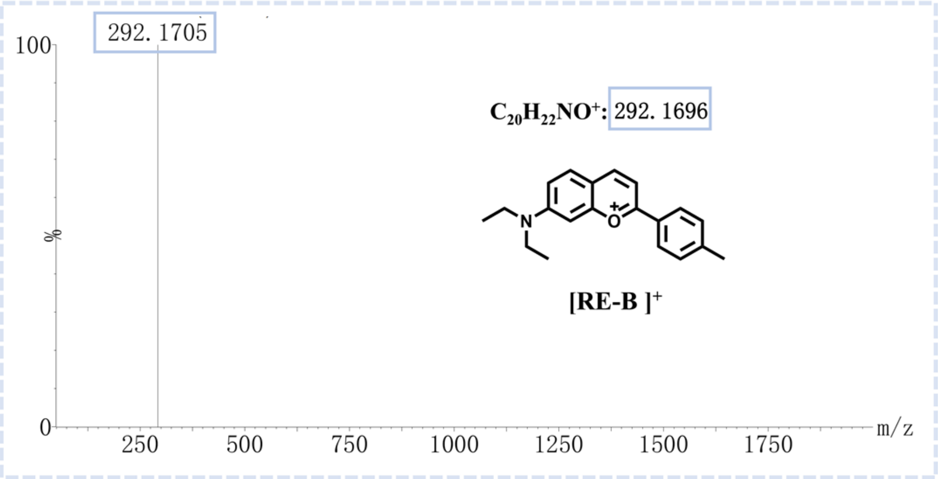

Figure S15. HRMS spectrum of **RE-B**.

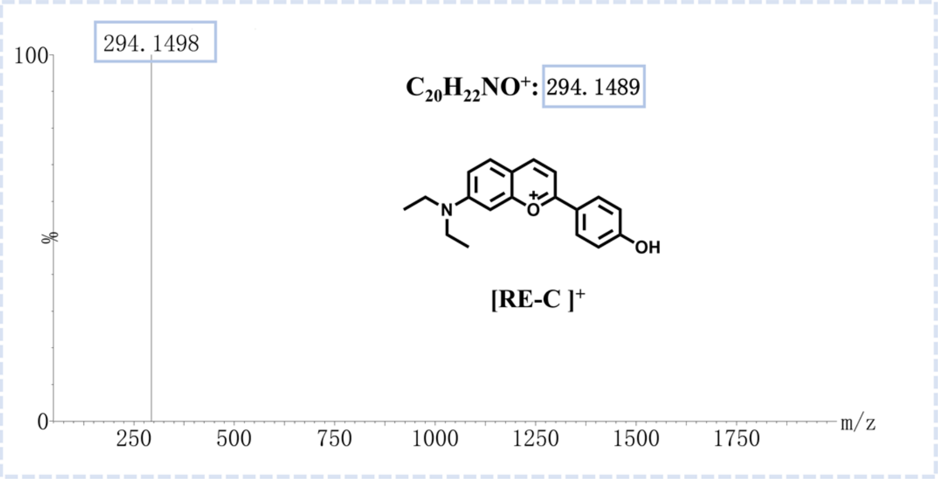

Figure S16. HRMS spectrum of **RE-C**.

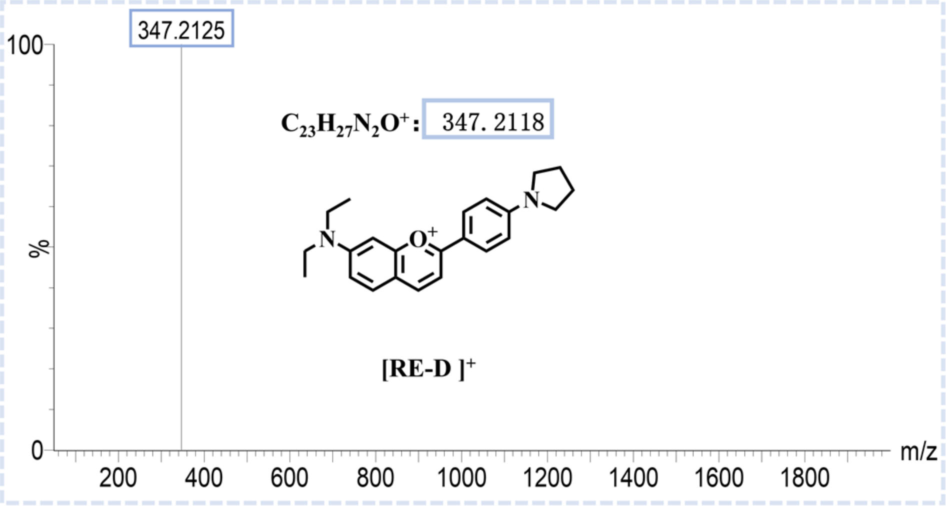

Figure S17. HRMS spectrum of **RE-D**.

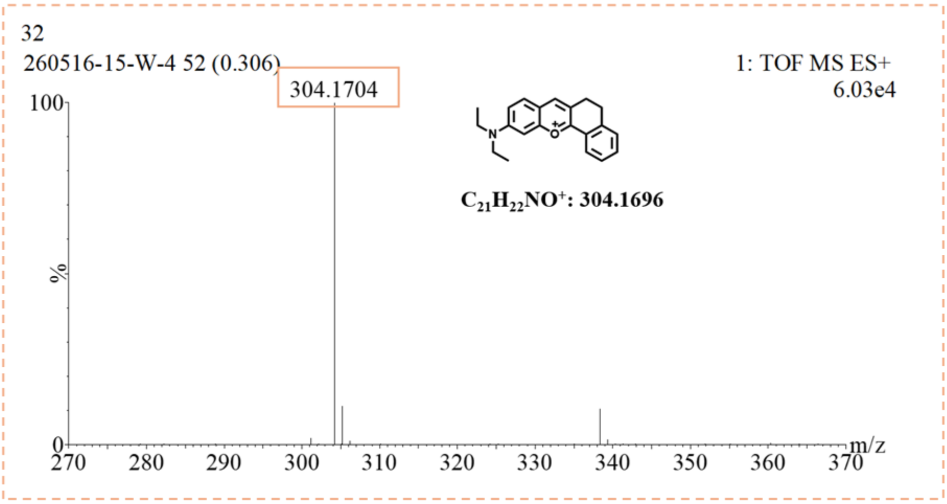

Figure S18. HRMS spectrum of **RF-1**.

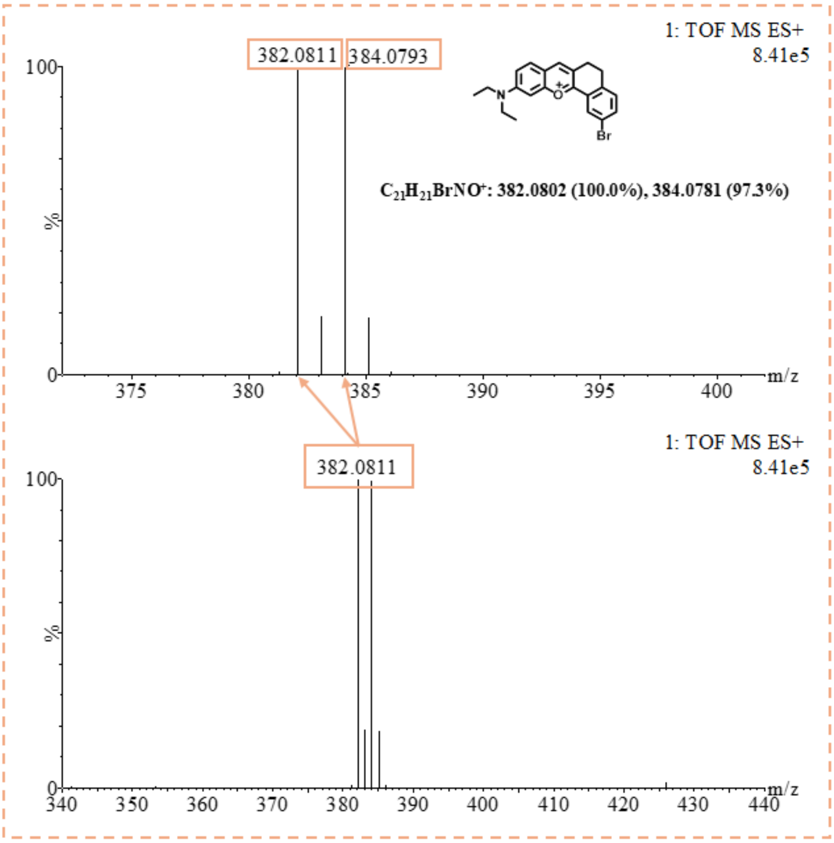

Figure S19. HRMS spectrum of **RF-2.**

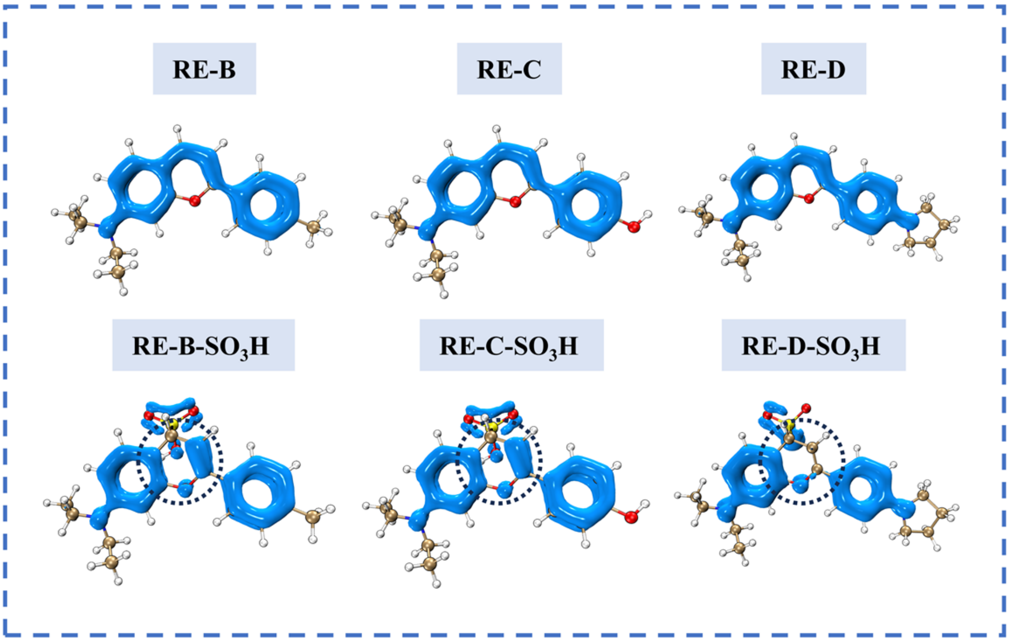

Figure S20. LOL-π isosurface map of **RE** probes.

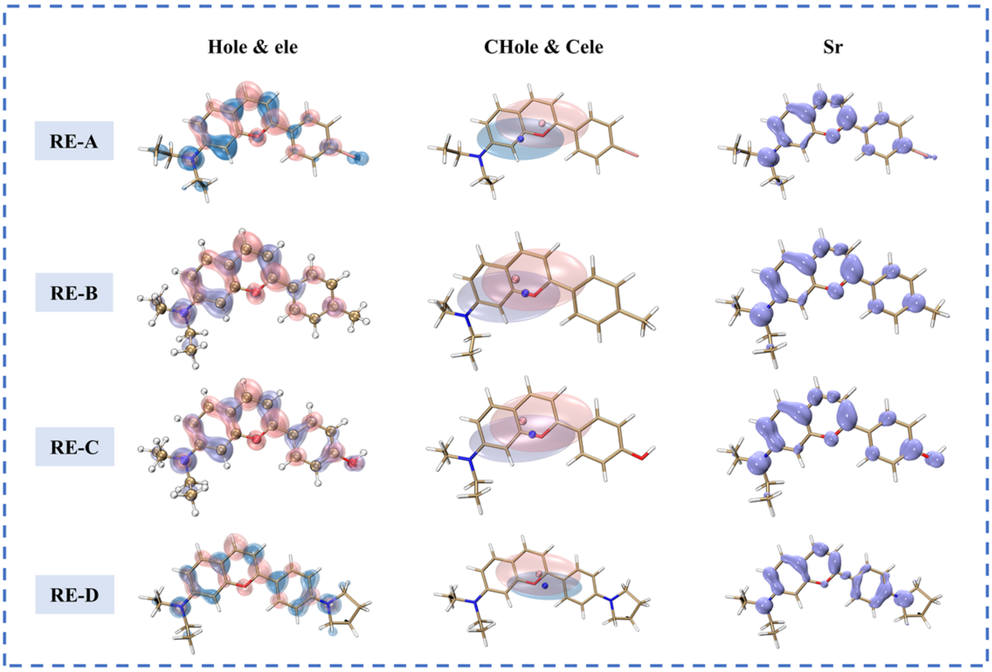

Figure S21. Hole-electron analysis of **RE** probes. (A) hole-electron distribution, (B) hole-electron centroid, (C) hole-electron Sr function. (hole: blue, electron: pink)

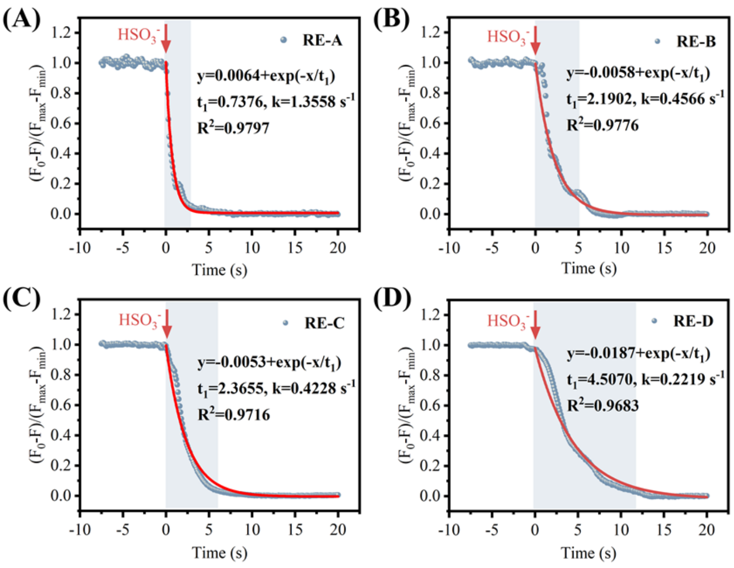

Figure S22. Fluorescence intensity-time curve monitoring of the reaction between **RE-X** (10 μM) and $NaHSO_3$ (500 μM). (A) **RE-A**; (B) **RE-B**; (C) **RE-C**; (D) **RE-D**.

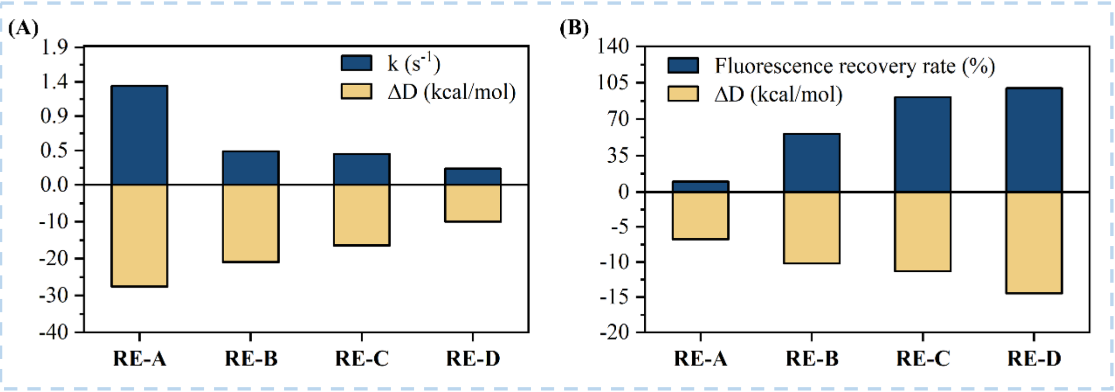

Figure S23. The relationship between the driving force of electrostatic potential imbalance (ΔD) and the kinetic reaction rate and thermodynamic equilibrium of the reversible SO_2-FA reaction. (A) The relationship between ΔD and the reaction rate constant k for the reaction of **RE-X** with sodium bisulfite. (B) The relationship between ΔD and the extent of fluorescence recovery for the reaction of **RE-X-SO_3H** with FA

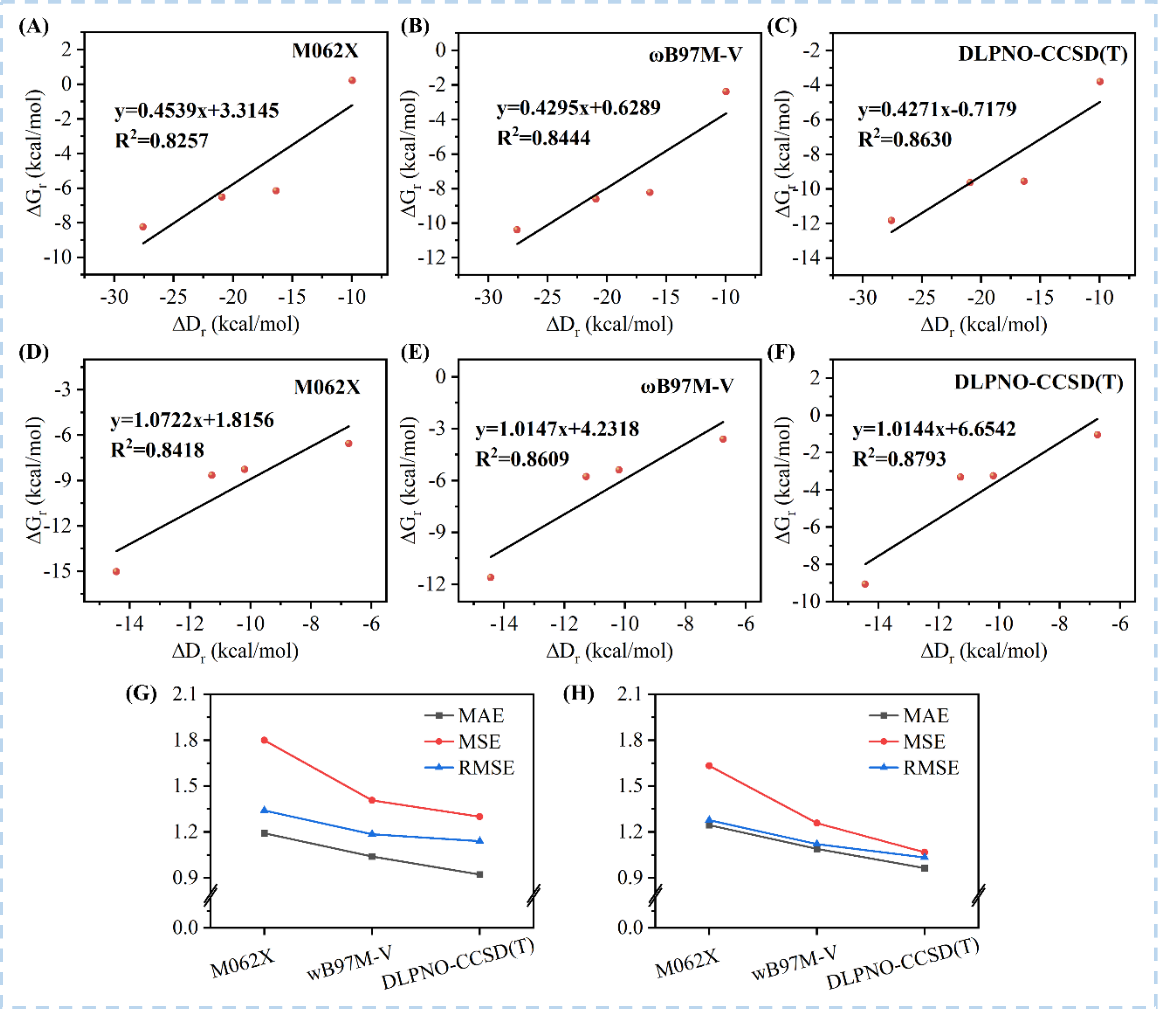

Figure S24. Linear fitting of D versus G for Reaction 1 at different computational levels: (A) M06-2X; (B) ωB97M-V; (C) DLPNO-CCSD(T); Linear fitting of D versus G for Reaction 2 at different computational levels: (D) M06-2X; (E) ωB97M-V; (F) DLPNO-CCSD(T); (G) and (H) Values of correlation metrics (MAE, MSE, and RMSE) for D versus G in Reaction 1 (G) and Reaction 2 (H) at different computational levels

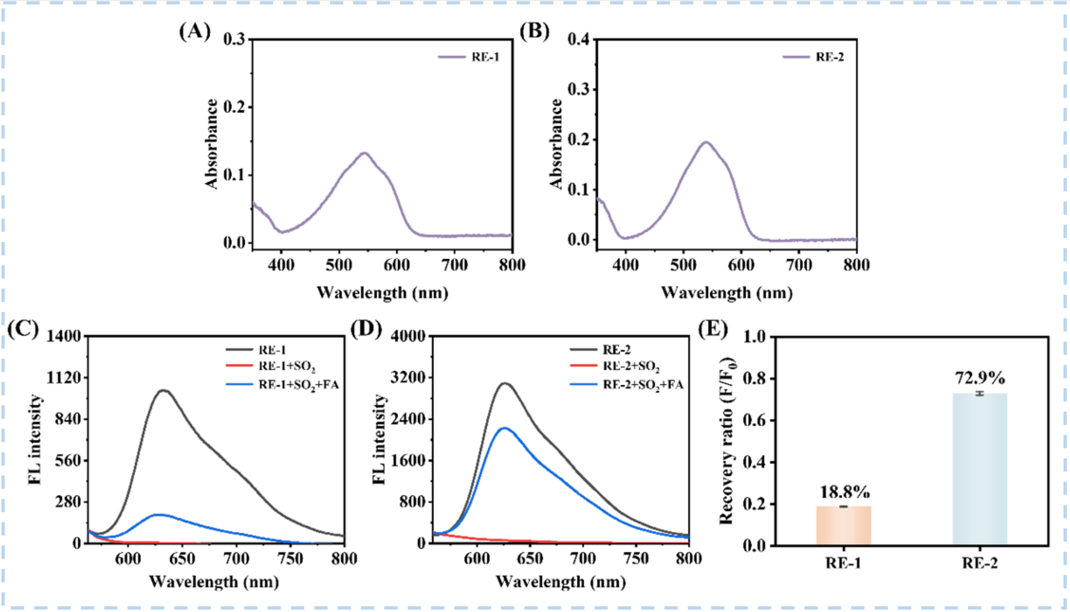

Figure S25. (A) Absorption spectra of the probe **RF-1** (10 μM); (B) Absorption spectra of the probe **RF-2** (10 μM); (C)Fluorescence spectra of **RF-1** (10 μM) before and after response with $NaHSO_3$ and FA (D) Fluorescence spectra of **RF-2** (10 μM) before and after response with $NaHSO_3$ and FA; (E)Fluorescence recovery ratio (F/F_0) of different **RF** probes

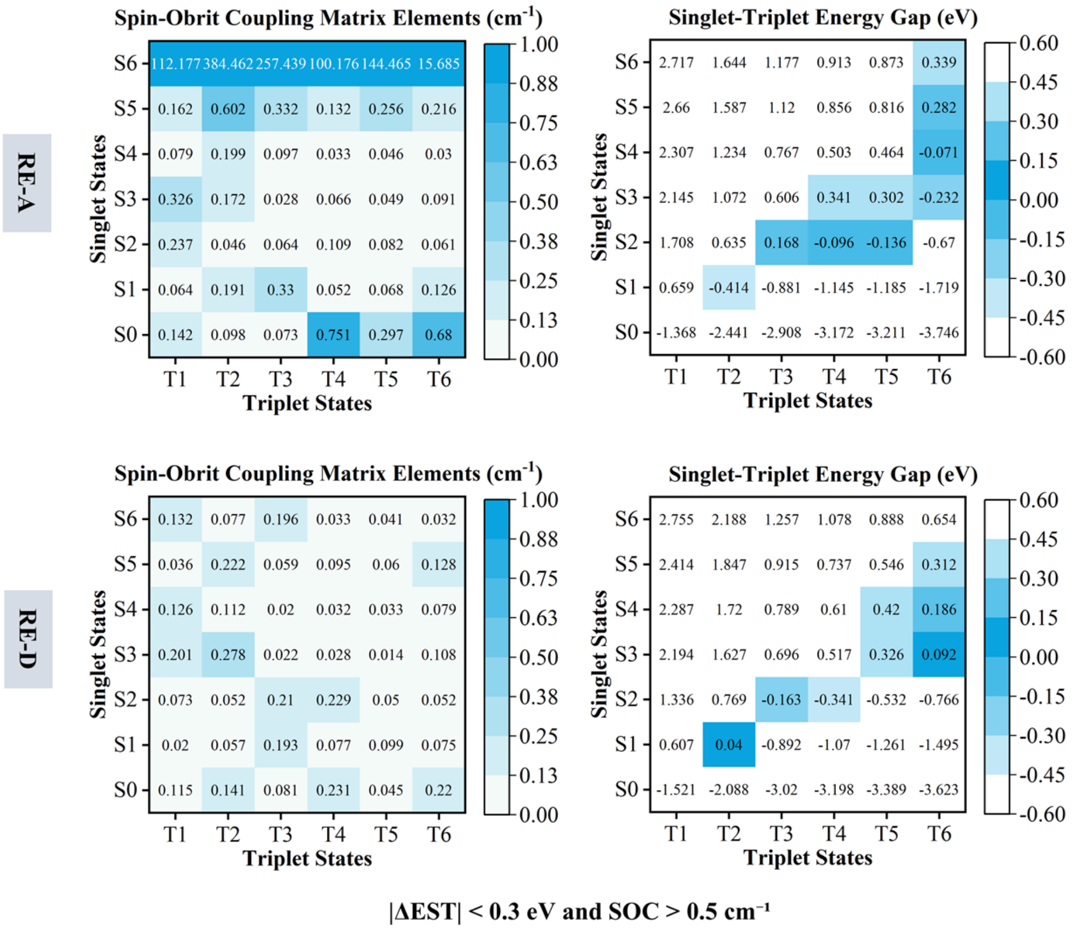

Figure S26. Heat map of spin-orbit coupling matrix element values between S_n-T_n states and singlet-triplet energy gap matrix for probes **RE-A** and **RE-D**.

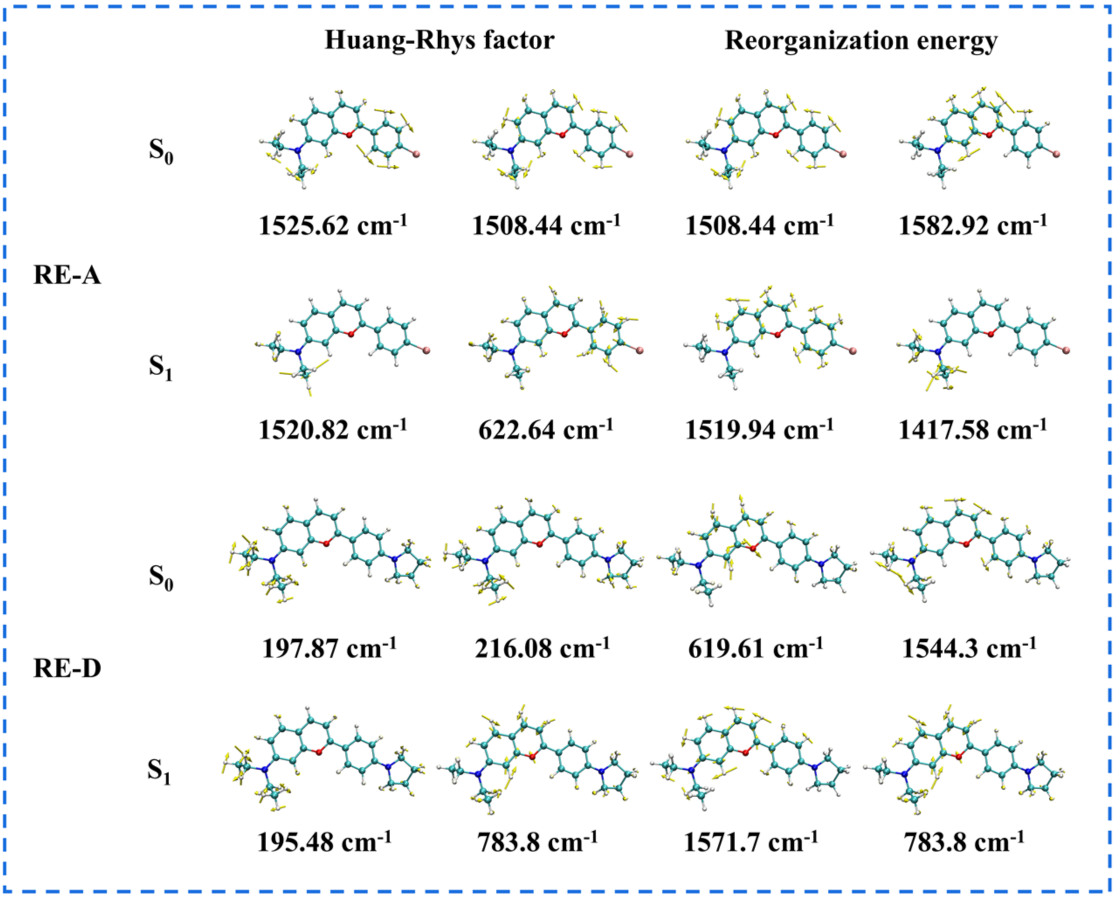

Figure S27. The representative normal modes of **RE-A** and **RE-D** in S_0 and S_1 states and their Huang-Rhys factors and reorganization energies.

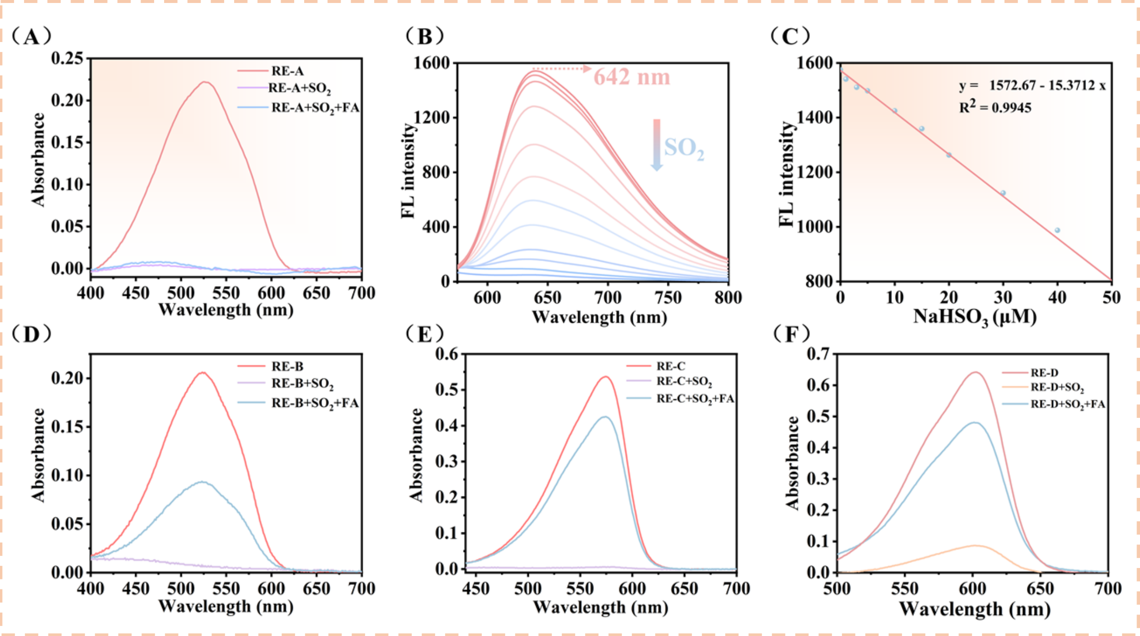

Figure S28. (A) Absorption spectra of probe **RE-A** (10 μM) before and after response to $NaHSO_3$ and FA. (B) Fluorescence spectra of the probe **RE-A** (10 μM) in response to different concentrations of $NaHSO_3$ (500 μM). (C) Linear relationship between the fluorescence intensity of the probe **RE-A** (10 μM) and $NaHSO_3$ concentration (0 - 50 μM). (D) Absorption spectra of probe **RE-B** (10 μM) before and after response to $NaHSO_3$ and FA. (E) Absorption spectra of probe **RE-C** (10 μM) before and after response to $NaHSO_3$ and FA. (F) Absorption spectra of probe **RE-D** (10 μM) before and after response to $NaHSO_3$ and FA.

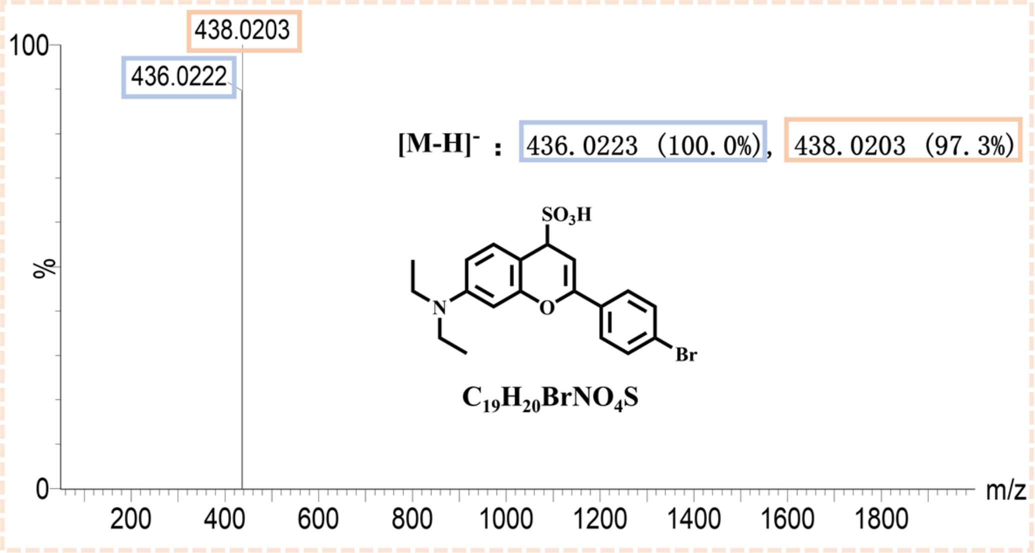

Figure S29. HRMS spectrum of the reaction of **RE-A** with $NaHSO_3$.

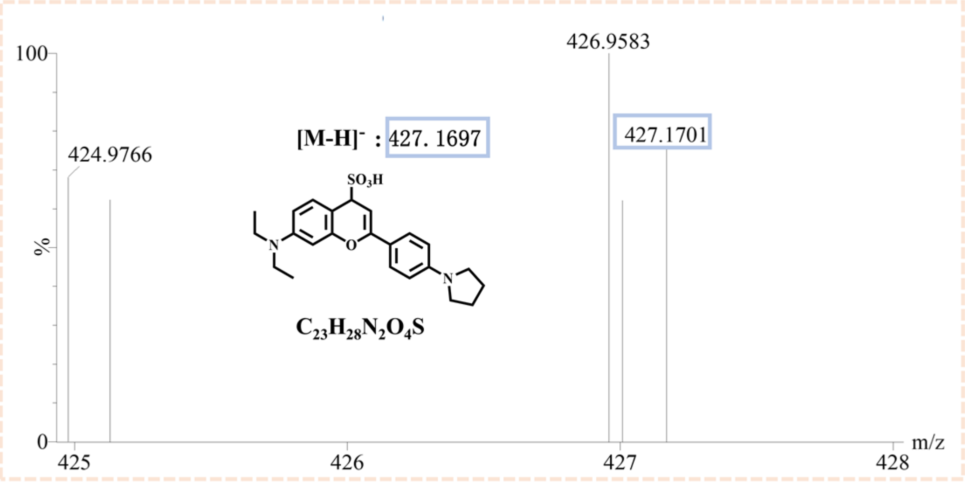

Figure S30. HRMS spectrum of the reaction of **RE-D** with $NaHSO_3$.

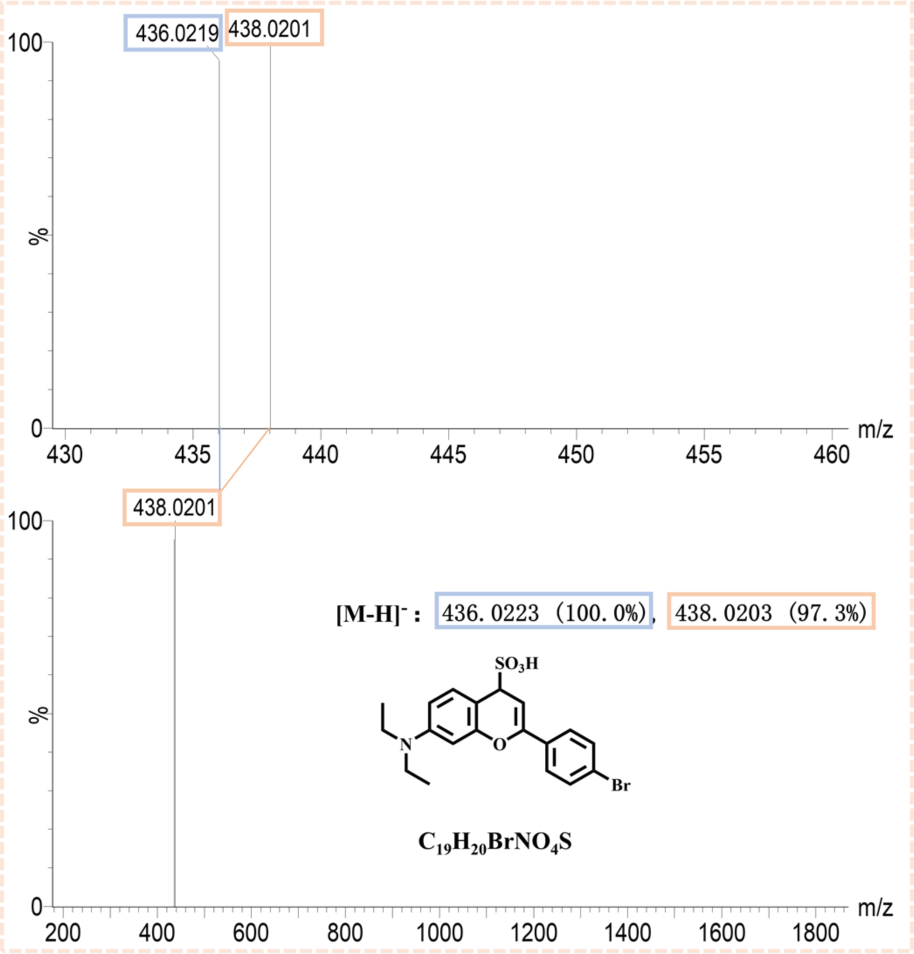

Figure S31. HRMS spectrum of the mixture of **RE-A** with $NaHSO_3$ upon addition of FA.

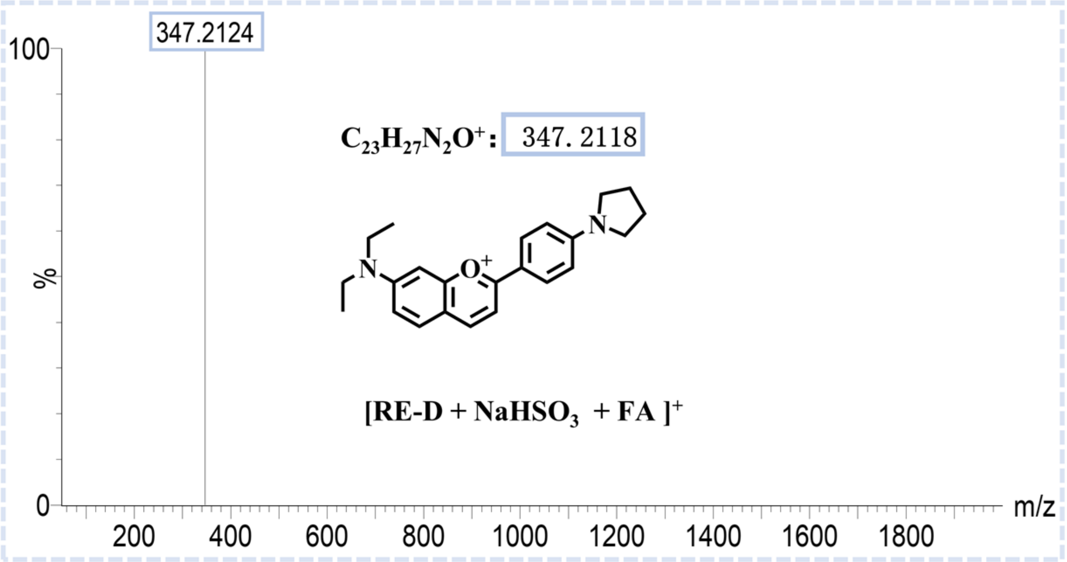

Figure S32. HRMS spectrum of the mixture of **RE-D** with $NaHSO_3$ upon addition of FA.

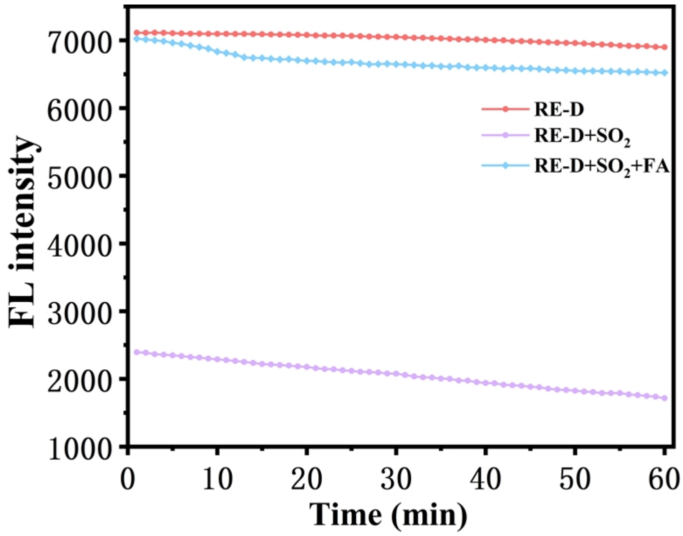

Figure S33. Photostability of **RE-D** (10 μM) toward $NaHSO_3$ (500 μM) and FA (1 mM) in PBS buffer (10 mM, 5 % CH_3CN, pH=7.4).

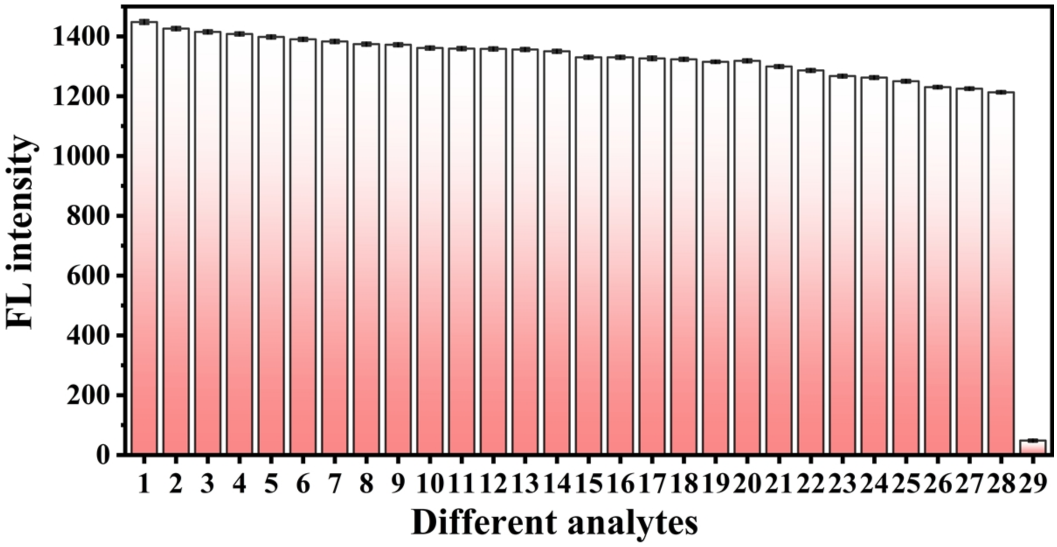

Figure S34. FL intensity of **RE-A** (10 μM) after addition of various analytes in PBS buffer (10 mM, 5 % CH_3CN, pH=7.4). 1, only the probe **RE-A** (10 μM); 2, Hcy; 3, NaHS; 4, NaClO; 5, Cys ; 6, GSH; 7, H_2O_2; 8, tert-butyl hydroperoxide (TBHP) ; 9, di-t-butyl peroxide (DTBP) ; 10, NaF; 11, KI ; 12, $CuCl_2$; 13, KSCN ; 14, $MgCl_2$; 15, NaBr ; 16, NaSCN ; 17, $NaHCO_3$; 18, $NaNO_2$; 19, L-Ascorbic acid ; 20, $NaNO_3$; 21, $Na_2S_2O_3$; 22, Na_2SO_4 ; 23, $ZnCl_2$; 24, NaOAc ; 25, $Fe_2(SO_4)_3$; 26, $FeSO_4$; 27, ONOO-; 28, H_2S_2; 29, $NaHSO_3$. Among them: the concentrations of 2-29 are 500 μM, Error bars represent mean values ± SD. (n = 6)

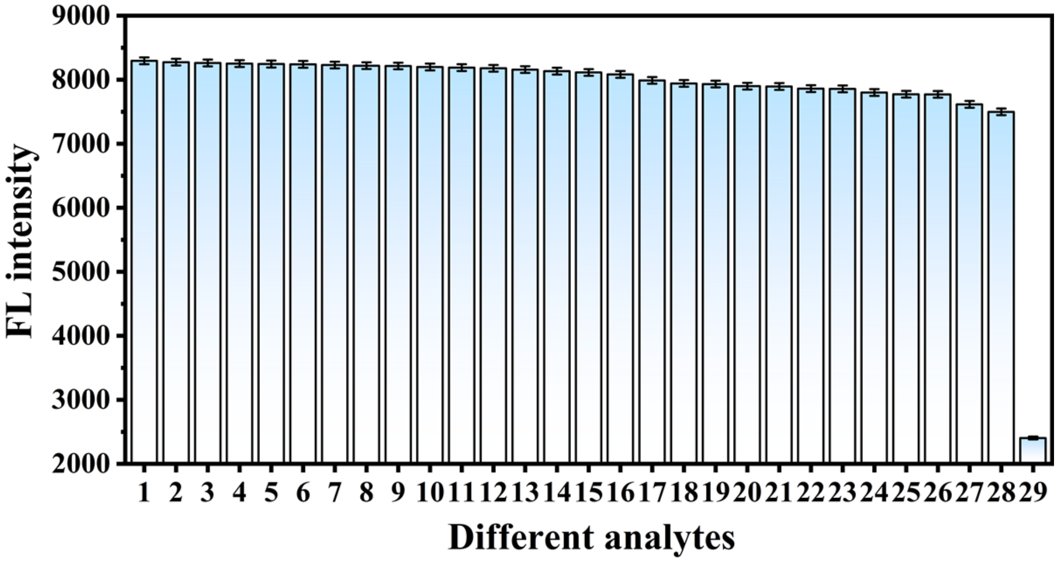

Figure S35. FL intensity of **RE-D** (10 μM) after addition of various analytes in PBS buffer (10 mM, 5 % CH_3CN, pH=7.4). 1, only the probe **RE-D** (10 μM); 2, Hcy; 3, NaHS; 4, NaClO; 5, NaSCN ; 6, Cys ; 7, GSH; 8, H_2O_2; 9, L-Ascorbic acid; 10, tert-butyl hydroperoxide (TBHP) ; 11, di-t-butyl peroxide (DTBP) ; 12, NaF; 13, KI ; 14, $CuCl_2$; 15, KSCN ; 16, $MgCl_2$; 17, NaBr ; 18, $NaHCO_3$; 19, $NaNO_2$; 20, $NaNO_3$; 21, $Na_2S_2O_3$; 22, Na_2SO_4 ; 23, $ZnCl_2$; 24, NaOAc ; 25, $Fe_2(SO_4)_3$; 26, $FeSO_4$; 27. $ONOO^-$; 28. H_2S_2; 29. $NaHSO_3$. Among them: the concentrations of 2-29 are 500 μM. Error bars represent mean values ± SD. (n = 6).

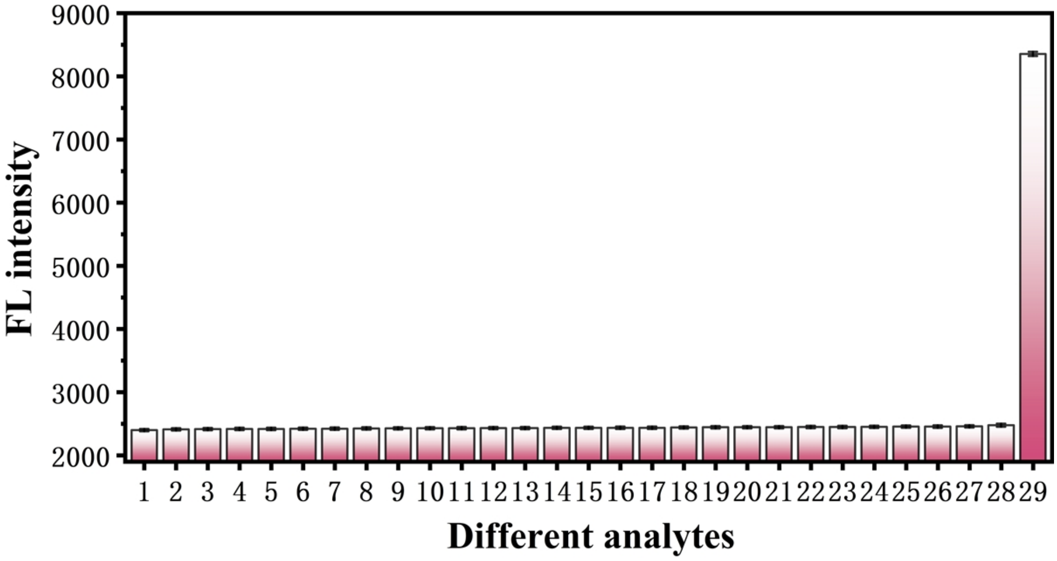

Figure S36. FL intensity of **RE-D** (10 μM) in the presence of 500 μM $NaHSO_3$ upon addition of various relevant analytes in PBS buffer (10 mM, 5 % CH_3CN, pH=7.4). 1, $NaHSO_3$; 2, $Na_2S_2O_3$; 3, NaClO; 4, $BaCl_2$; 5, $NaHCO_3$; 6, Na_2S ; 7, KF; 8, Na_2HPO_4 ; 9, 4-nitrobenzaldehyde ; 10, DTBP ; 11, $NaNO_2$; 12, CH_3CHO ; 13, Sodium pyruvate ; 14, KSCN ; 15, NH_2NH_2; 16, NaOAc ; 17, GSH ; 18, Na_2SO_4 ; 19, Citrate; 20, TBHP; 21, KI; 22, NaBr; 23, $AgNO_3$; 24, Cys; 25, H_2O_2; 26, Acetone ; 27, CHOCHO; 28, Benzaldehyde; 29, FA. Among them: the concentrations of 2-29 are 500 μM. Error bars represent mean values ± SD. (n = 6).

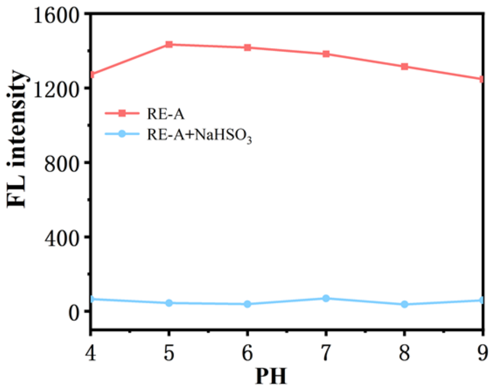

Figure S37. FL intensity changes of **RE-A** (10 μM) at different pH values in the absence or presence of $NaHSO_3$ (500 μM).

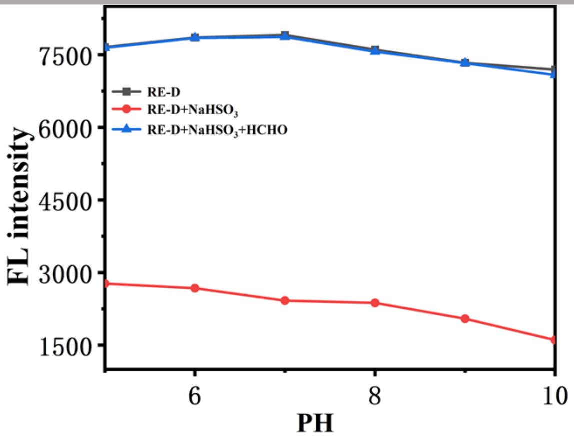

Figure S38. FL intensity changes of **RE-D** (10 μM) toward $NaHSO_3$ (500 μM) and FA (1 mM) at different pH values.

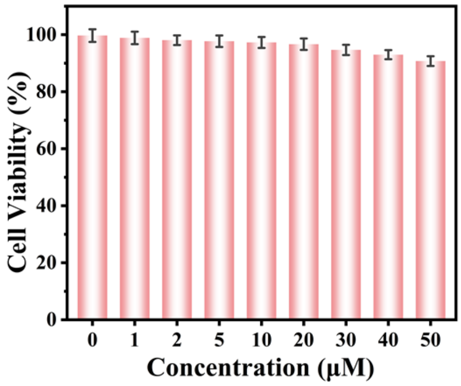

Figure S39. Viability of HepG2 cells treated with various concentrations (0 - 50 μM) of **RE-D** for 24 h. Error bars represent mean values ± SD. (n = 6).

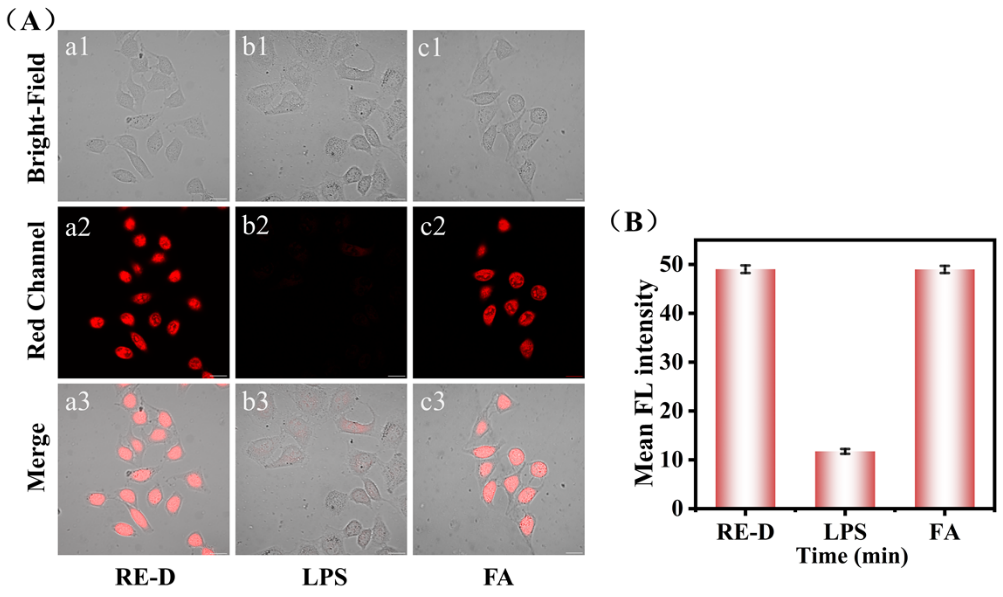

Figure S40. （A） Fluorescence images of **RE-D** for detecting endogenous SO_2 in living cells. (a1-a3) The HepG2 cells were treated with 10 μM **RE-D**. (b1-b3) HepG2 cells were pretreated with 1 μg/mL LPS for 6 h followed by 10 μM **RE-D**. (c1-c3) HepG2 cells were pretreated with 1 μg/mL LPS for 6 h followed by addition 10 μM **RE-D**, and then incubated with 200 μM FA. (B) Quantification of mean fluorescence intensity in HepG2 cells. λ_{ex} = 561 nm，λ_{em} = 570-670 nm. Error bars represent mean values ± SD (n=6). Scale bar: 20 μm.

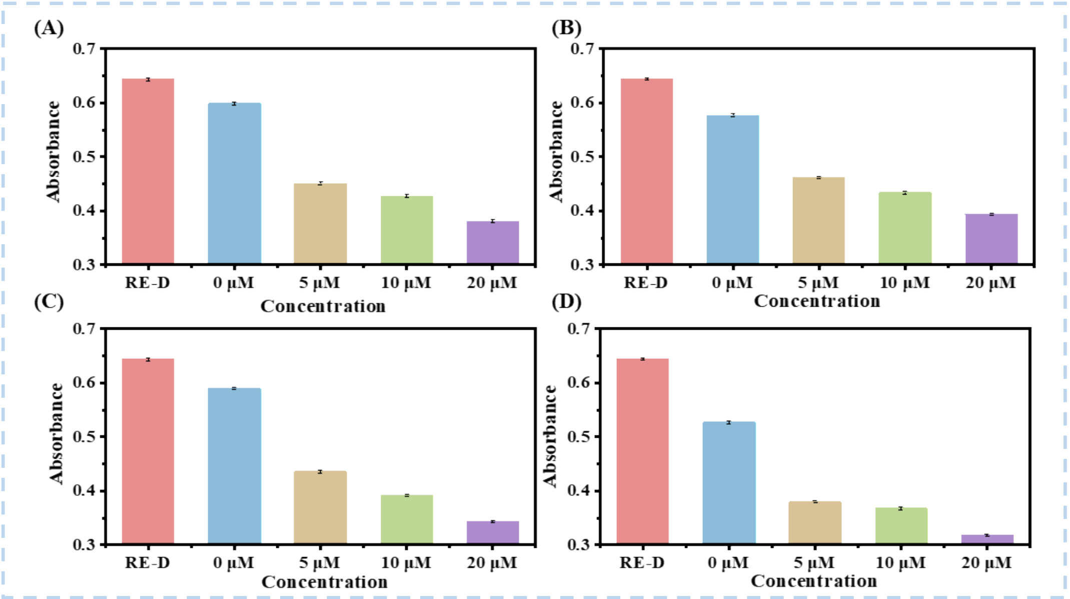

Figure S41. Absorbance of filtrates from four Chinese medicinal materials spiked with varying SO_2 concentrations (0, 5, 10, 20 μM) after addition of probe **RE-D** (10 μM): (A) Chinese yam; (B) Kudzu powder; (C) Poria cocos; (D) Angelica dahurica.

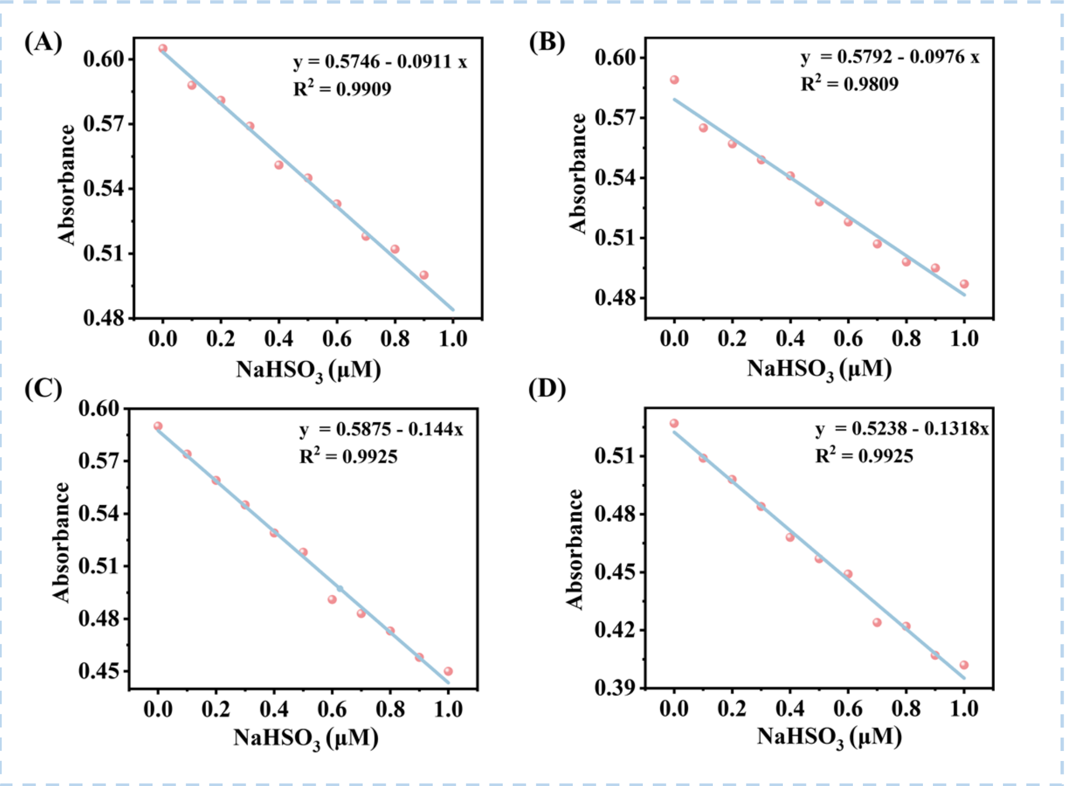

Figure S42. Standard addition calibration curves were constructed for SO_2 detection in Chinese yam (A), kudzu (B), Poria cocos (C), and Angelica dahurica (D) matrices using probe **RE-D** (10 μM) over the spiked concentration range of 0–1 μM.

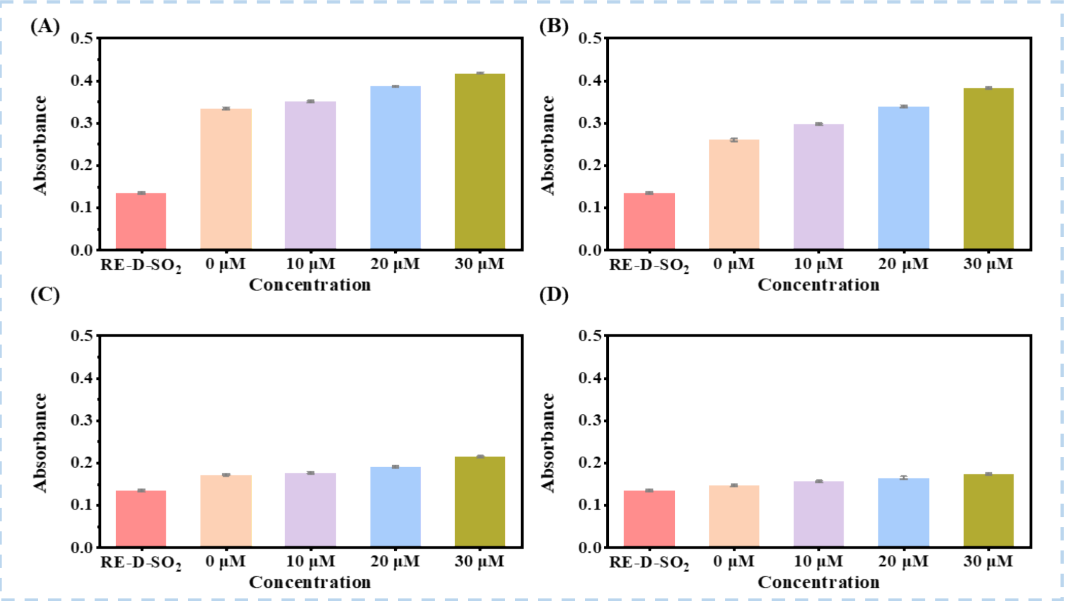

Figure S43. Absorbance of filtrates from four nail polish samples spiked with varying FA concentrations after addition of **RE-D-SO$_3$H** (10 μM **RE-D** + 500 μM $NaHSO_3$): (A) Nail polish 297; (B) Nail polish 301; (C) Nail polish 299; (D) Nail polish 300.

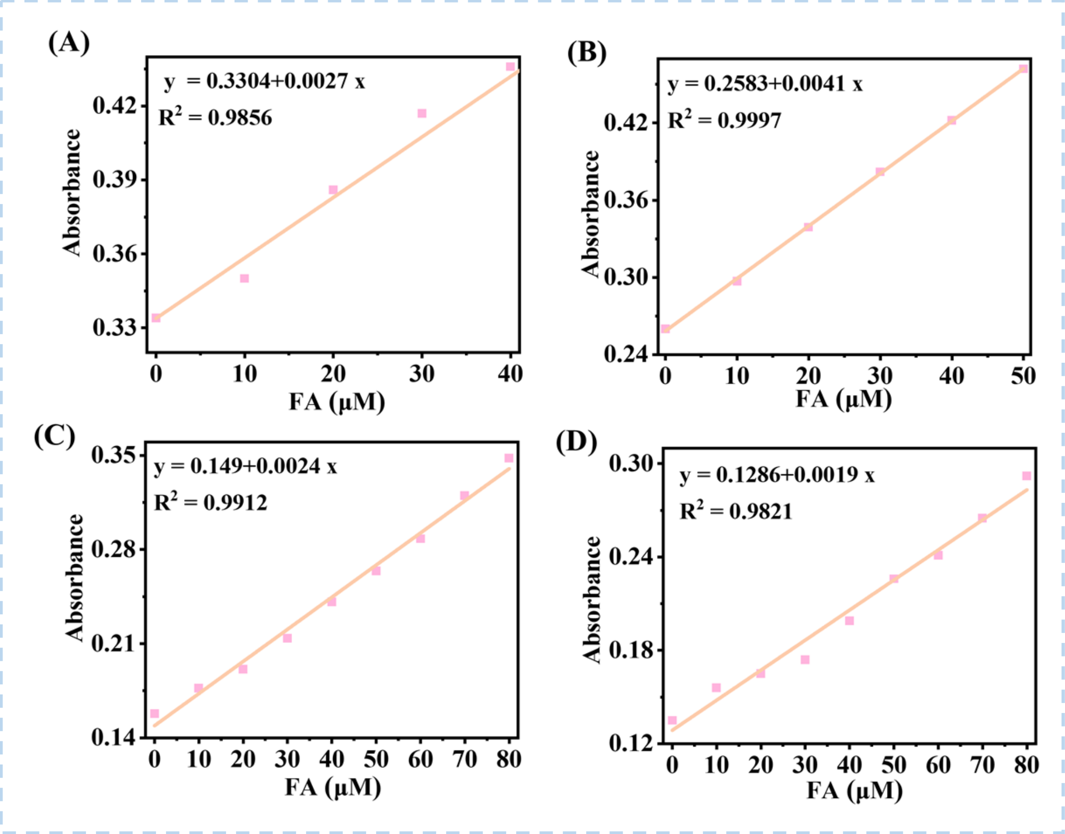

Figure S44. Standard addition calibration curves for FA detection in four nail polish matrices using **RE-D-SO_3H** (10 μM **RE-D** + 500 μM $NaHSO_3$): (A) nail polish 297, (B) nail polish 301, (C) nail polish 299, and (D) nail polish 300.

References:

1 A. D. Becke, *The Journal of Chemical Physics*, 1993, **98**, 5648–5652.

2 A. Schäfer, H. Horn and R. Ahlrichs, *The Journal of Chemical Physics*, 1992, **97**, 2571–2577.

3 A. Schäfer, C. Huber and R. Ahlrichs, *The Journal of Chemical Physics*, 1994, **100**, 5829–5835.

4 A comprehensive electron wavefunction analysis toolbox for chemists, Multiwfn | The Journal of Chemical Physics | AIP Publishing, https://doi.org/10.1063/5.0216272, (accessed May 15, 2025).

5 T. Lu and F. Chen, *J Comput Chem*, 2012, **33**, 580–592.

6 T. Lu and Q. Chen, *Theor Chem Acc*, 2020, **139**, 25.

7 Z. Liu, T. Lu and Q. Chen, *Carbon*, 2020, **165**, 461–467.

8 J. Zhang and T. Lu, *Phys. Chem. Chem. Phys.*, 2021, **23**, 20323–20328.

9 T. Lu, Section [3.21.8] of Multiwfn manual version [3.8(dev)] (accessed May 5, 2024) available at http://sobereva.com/multiwfn.

10 T. Lu, *J Mol Model*, 2021, **27**, 263.

11 W. Humphrey, A. Dalke and K. Schulten, *Journal of Molecular Graphics*, 1996, **14**, 33–38.

12 Z. Shuai and Q. Peng, *Physics Reports*, 2014, **537**, 123–156.

13 Y. Niu, W. Li, Q. Peng, H. Geng, Y. Yi, L. Wang, G. Nan, D. Wang and Z. Shuai, *Molecular Physics*, 2018, **116**, 1078–1090.

14 Z. Shuai and Q. Peng, *National Science Review*, 2017, **4**, 224–239.

15 Y. Niu, Q. Peng and Z. Shuai, *Sci. China Ser. B-Chem.*, 2008, **51**, 1153–1158.

16 Z. Shuai, *Chin. J. Chem.*, 2020, **38**, 1223–1232.

17 Q. Peng, Y. Yi, Z. Shuai and J. Shao, *J. Am. Chem. Soc.*, 2007, **129**, 9333–9339.

18 S. Grimme, J. Antony, S. Ehrlich and H. Krieg, *The Journal of Chemical Physics*, 2010, **132**, 154104.

19 Y. Guo, C. Riplinger, U. Becker, D. G. Liakos, Y. Minenkov, L. Cavallo and F. Neese, *The Journal of Chemical Physics*, 2018, **148**, 011101.

20 D. G. Liakos and F. Neese, *J. Chem. Theory Comput.*, 2015, **11**, 4054–4063.

21 Y. Zhao and D. G. Truhlar, *Theor Chem Account*, 2008, **120**, 215–241.

22 A. V. Marenich, C. J. Cramer and D. G. Truhlar, *J. Phys. Chem. B*, 2009, **113**, 6378–6396.

23 N. Mardirossian and M. Head-Gordon, *The Journal of Chemical Physics*, 2016, **144**, 214110.
